# Synthesis, Antifungal Activity, 3D-QSAR, and Molecular Docking Study of Anethole-Based Thiazolinone-Hydrazone Compounds

**DOI:** 10.3390/molecules31071078

**Published:** 2026-03-25

**Authors:** Yao Chen, Yu-Cheng Cui, You-Qiong Bi, Zhang-Li Guo, Xian-Li Ma, Wen-Gui Duan, Gui-Shan Lin

**Affiliations:** 1 School of Chemistry and Chemical Engineering, Guangxi University, Nanning 530004, China; cyygxu2026@163.com (Y.C.); cuiyc1109@126.com (Y.-C.C.); biyouqiong1021@163.com (Y.-Q.B.); guo08292026@163.com (Z.-L.G.); mxl78@glmc.edu.cn (X.-L.M.); 2 Guangxi Key Laboratory for Pharmaceutical Molecular Discovery and Druggability Optimization, Guilin Medical University, Guilin 541199, China

**Keywords:** anethole, thiazolinone-hydrazone, antifungal activity, 3D-QSAR, molecular docking, DFT calculations

## Abstract

In order to find green fungicides derived from natural products, 22 unreported anethole-based thiazolinone-hydrazone compounds were designed and synthesized, and their structures were characterized by FT-IR, ^1^H NMR, ^13^C NMR, and HRMS. At a concentration of 50 mg/L, the preliminary antifungal activity of the target compounds against eight plant pathogens was evaluated. The results showed that **5q** (R = *m*-OH C_6_H_4_) exhibited the best inhibitory activity against most of the tested plant pathogenic fungi, demonstrating that this compound had certain broad-spectrum antifungal activity. In addition, a reasonable and effective 3D-QSAR model (*r*^2^ = 0.994, *q*^2^ = 0.529) was established using the comparative molecular field analysis (CoMFA) method to study the relationship between the structures of the target compounds and their antifungal activity against *Physalospora piricola*. Meanwhile, the results of electrostatic potential calculation of the compounds indicated that the electronic effect caused by different substituents on the benzene ring might be one of the factors affecting antifungal activity. In addition, frontier molecular orbital calculations implied that the anethole moiety and the thiazolinone-hydrazone-benzene structure in the target compounds might play an important role in antifungal activity. The potential binding mode between the target compound **5q** (R = *m*-OH C_6_H_4_) and the homology-modeled succinic dehydrogenase was explored by molecular docking.

## 1. Introduction

As culprits of plant diseases, plant pathogenic fungi not only cause significant economic losses and post-harvest damage but also pose threats to plant safety [1]. To address these challenges, various types of fungicides were widely used in agriculture over the long term [2,3]. However, the prolonged application of commercial fungicides led to increased resistance in plant pathogenic fungi, which reduced the control effectiveness of plant disease and raised both the cost of pesticide use and the environmental risks associated with pesticide residues [4]. Therefore, developing efficient, green, and broad-spectrum new fungicides to overcome existing resistance was of great importance. In this context, the availability and abundance of natural products offered a promising pathway for the development of new antifungal agents [5,6,7].

Anethole, a natural organic compound featuring the characteristic C6–C3 scaffold of phenylpropanoids [8,9], is primarily obtained from the fruits and leaves of star anise with a high annual yield [10]. This highly aromatic and volatile essential oil finds wide application in the cosmetics and food industries. Moreover, it exhibits a broad spectrum of biological activities, including antitumor [11], cardiovascular protective [12], antidiabetic [13], insecticidal [14], antifungal [15,16], anti-inflammatory [17], and antioxidant effects [18]. Thiazolinones, a class of five-membered heterocycles containing nitrogen, sulfur, and a carbonyl moiety within the ring system, possess an internal amide bond. This structure confers a strong ability to form hydrogen bonds with diverse biological targets, leading to wide-ranging bioactivities such as anticonvulsant [19], insecticidal [20], antibacterial, and antitumor effects [21]. Hydrazones, formed by the condensation of carbonyl compounds with hydrazine and characterized by a carbon–nitrogen double bond, also hold significant importance in agriculture and medicine. They demonstrate notable biological activities, including antioxidant [22], antifungal [23], and antiepileptic properties [24]. Among contemporary commercial fungicides, succinate dehydrogenase inhibitors (SDHIs) represent the most rapidly growing and promising class [25]. It was found from the existing relevant reports and our prior research experience in the design of green active antifungal molecules [26,27,28,29,30] that groups such as thiazole rings and hydrazones play crucial pharmacodynamic roles in targeting succinate dehydrogenase (SDH). Therefore, we hope to make a preliminary investigation into the potential action mechanism of anethole-derived thiazolinone-hydrazone derivatives.

Based on the aforementioned rationale and our previous research on bioactive natural product derivatives [2,4,30,31,32], a series of unreported thiazolinone-hydrazone derivatives were designed and synthesized employing the principle of active functional group splicing. These compounds feature a natural anethole-derived scaffold, constructed by integrating the bioactive thiazolinone and hydrazone moieties into the anethole (*E*)-4-methoxycinnamaldehyde molecular framework, which itself is prepared by oxidation of the allylic methyl group of (*E*)-anethole. Their in vitro antifungal activities against eight plant pathogenic fungi were subsequently evaluated. Furthermore, the structure-activity relationships and potential binding modes were investigated through a 3D-QSAR model and molecular docking studies, respectively.

## 2. Results and Discussion

### 2.1. Synthesis and Characterization

**Scheme 1:**
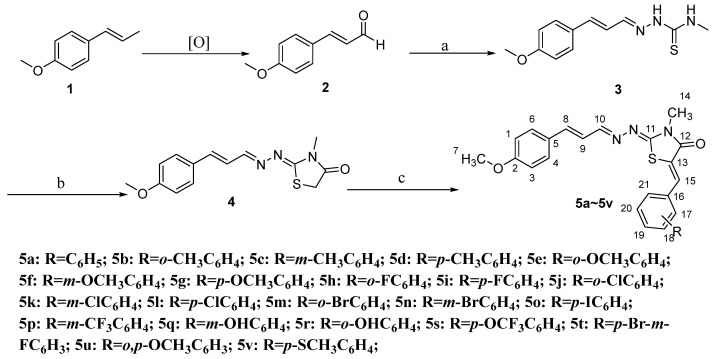
Synthesis of anethole-based thiazolinone-hydrazone compounds **5a**–**5v**. **Reagents and conditions:** (**a**) 4-methylaminothiourea, EtOH, 80 °C, 12 h; (**b**) ethyl bromoacetate, EtONa, EtOH, 80 °C, 4 h; (**c**) substituted benzaldehydes, KOH, EtOH, 80 °C, 6 h.

As illustrated in Scheme 1, the target compounds **5a**–**5v** were synthesized from 4-methoxycinnamaldehyde via the multi-step procedure. Initially, according to our previously reported method, 4-methoxycinnamaldehyde **2** was obtained through oxidation of the allylic methyl group of anethole [31]. Then, intermediate **2** and 4-methylaminothiourea underwent a condensation reaction catalyzed by hydrochloric acid to give intermediate **3**, an anethole-based thiosemicarbazide. Subsequently, intermediate **4** was prepared by cyclization of intermediate **3** with ethyl bromoacetate under basic conditions. Finally, intermediate **4** reacted with a series of substituted benzaldehydes by Knoevenagel condensation reaction to afford twenty-two thiazolinone-hydrazone target compounds **5a**–**5v** incorporating a natural anethole scaffold.

The structures of all target compounds were confirmed by ^1^H NMR, ^13^C NMR, HRMS, and FTIR spectra. In the ^1^H NMR spectra, signals in the range of *δ* 8.27–6.46 ppm were assigned to aromatic, olefinic, and imine protons, while resonances between *δ* 3.87–3.42 ppm were assigned to the methoxy and methyl groups. The ^13^C NMR spectra displayed signals at *δ* 166.4–157.3 ppm and *δ* 166.4–114.0 ppm, attributable to aromatic carbons and those of the thiazolinone-hydrazone moiety, respectively; peaks in the region of *δ* 55.3–29.8 ppm were assigned to methoxy and methyl carbons. The experimental molecular weight was consistent with the calculated molecular weight, as confirmed by HRMS. In the FT-IR spectra, absorption bands at 3072–3004 cm^−1^ and 2954–2833 cm^−1^ were assigned to unsaturated aromatic or olefinic and saturated C-H stretching vibrations, respectively. The carbonyl (C=O) stretching vibration appeared at 1706–1716 cm^−1^, while strong absorptions in the range of 1631–1429 cm^−1^ were attributed to C=C, C=N, and aromatic ring skeletal vibrations. All the related characterization data and spectra can be found in the Supplementary Materials.

### 2.2. In Vitro Antifungal Activity

The antifungal activities of the target compounds **5a**–**5v** were preliminarily tested in vitro against eight plant pathogenic fungi, including *Fusarium oxysporum* f. sp. *Cucumerinum* (*F. oxysporum* f. sp. *cucumerinum*), *Cercospora arachidicola* (*C. arachidicola*), *Physalospora piricola* (*P. piricola*), *Alternaria solani* (*A. solani*), *Gibberella zeae* (*G. zeae*), *Rhizoctonia solani* (*R. solani*), *Bipolaris maydis* (*B. maydis*), and *Colletotrichum orbiculare* (*C. orbiculare*). The results at a concentration of 50 mg/L are shown in Table 1. The commercial antifungal boscalid was used as a positive control.

Experimental evaluation revealed that compounds **5a**–**5v** exhibited certain antifungal activities against the tested eight plant pathogenic fungi. Notably, **5q** (R = *m*-OH C_6_H_4_) showed the best inhibitory activity against seven of the tested fungi (except *R. solani*), in which the inhibition rates against *P. piricola*, *A. solani*, *C. arachidicola*, and *B. maydis* were 85. 0%, 72.5%, 71.3%, and 70. 6%, respectively, indicating that **5q** (R = *m*-OH C_6_H_4_) had certain broad-spectrum antifungal activity. Moreover, **5q** (R = *m*-OH C_6_H_4_) displayed better antifungal activity against seven of the tested fungi than that of the positive control boscalid (except *C. arachidicola*). Therefore, **5q** (R = *m*-OH C_6_H_4_) is a promising lead compound worthy of further study. Overall analysis suggested that target compounds bearing *m*-R on the benzene ring generally exhibited stronger antifungal activity than those with *o*-R or *p*-R. For example, the activities of **5q** (R = *m*-OH C_6_H_4_) and **5c** (R = *m*-CH_3_ C_6_H_4_) were higher than those of **5r** (R = *o*-OH C_6_H_4_), **5b** (R = *o*-CH_3_ C_6_H_4_), and **5d** (R = *p*-CH_3_ C_6_H_4_).

### 2.3. 3D-QSAR Analysis and Theoretical Calculation

**Figure 1:**
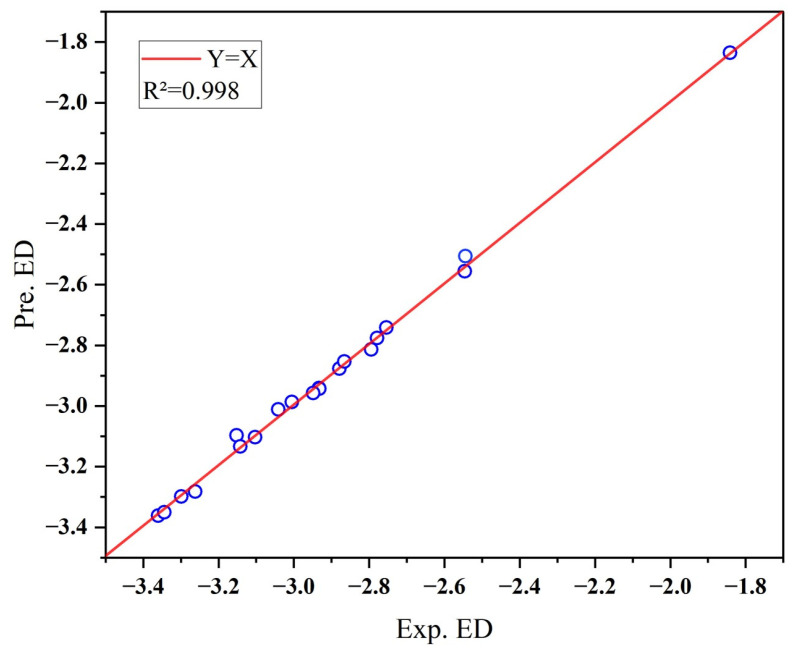
Predicted ED values of CoMFA model vs. experimental ED value.

To investigate the relationship between the structures of the target compounds and their antifungal activity against *P. piricola* in detail, the 3D-QSAR model was established using the CoMFA method in SYBYL 2.1.1 software [32]. The most active compound **5q** (R = *m*-OH C_6_H_4_) was selected as the template for model construction. As outlined in Table 2, nineteen target compounds were designated as the training set and three compounds as the test set. Prior to analysis, the structures of compounds **5a**–**5v** were energy-minimized using the Tripos force field with Gasteiger–Hückel charges, in accordance with established protocols. The statistical parameters of the established CoMFA model were summarized in Table 3. The high non-cross-validated correlation coefficient (*r*^2^ = 0.994), low standard error of estimate (*S* = 0.033), high Fisher ratio (*F* = 116.271), and acceptable cross-validated correlation coefficient (*q*^2^ = 0.529) collectively indicated that the model was robust and reliable. Furthermore, the predictive ability of the model was supported by the scatter plot of experimental versus predicted biological activities (Exp. ED and Pre. ED values), shown in Figure 1. All data points clustered closely around the Y = X line, implying that this model had a good predictive performance.

**Figure 2:**
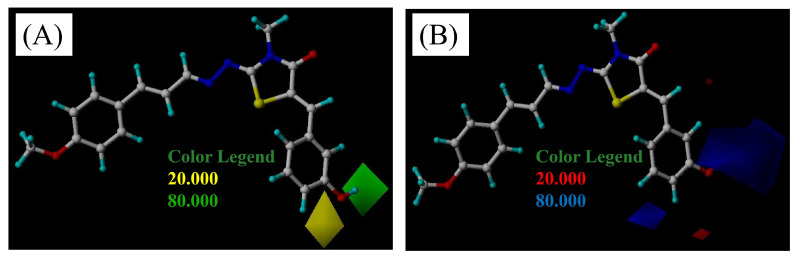
Contours of CoMFA analysis: (**A**) contours of steric contribution are represented in yellow and green; (**B**) contours of electrostatic contribution are represented in red and blue.

As summarized in Table 3, the steric and electrostatic field contributions to the model were 52.3% and 47.7%, respectively. It indicated that the steric effect of the R group played a dominant role in the antifungal activity of anethole-based thiazolinone hydrazone compounds, while electronic effects also had some influence. The corresponding field contours are shown in Figure 2. In the steric field map (Figure 2A), green contours indicate regions where introducing bulky substituents was favorable for activity, while yellow contours indicate regions where bulky substituents were unfavorable. A distinct green region can be observed near the *meta* position of the benzene ring, whereas a yellow region appeared near the *para* position, suggesting that introducing larger substituents at the *meta* position helped enhance antifungal activity. Compared to compounds with bulky groups at the *ortho* and *para* positions, *meta*-substituted analogues exhibited higher activity, such as **5q** (R = *m*-OH C_6_H_4_), **5p** (R = *m*-CF_3_ C_6_H_4_), and **5k** (R = *m*-Cl C_6_H_4_). In the electrostatic potential map (Figure 2B), the blue contours indicated regions where electron-donating groups enhanced activity, while the red contours suggested that electron-withdrawing groups were more favorable. For example, the higher activity of **5q** (R = *m*-OH C_6_H_4_) and **5c** (R = *m*-CH_3_ C_6_H_4_) compared to **5k** (R = *m*-Br C_6_H_4_) and **5p** (R = *m*-CF_3_ C_6_H_4_) was consistent with the presence of blue regions at the *meta* position, supporting the positive effect of electron-donating properties at this position.

**Figure 3:**
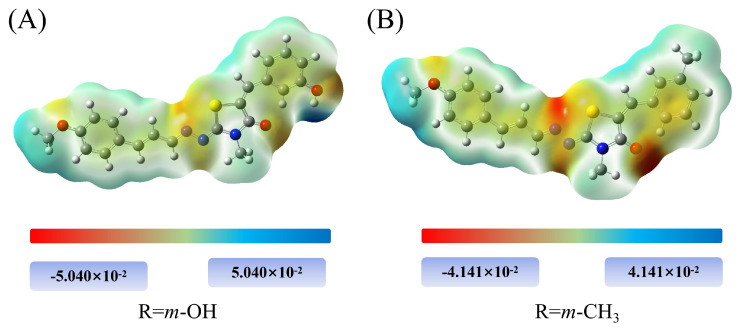
Electrostatic potential maps of the compounds: **5q** (R = *m*-OH C_6_H_4_, (**A**)) and **5c** (R = *m*-CH_3_ C_6_H_4_, (**B**)).

To further investigate the relationship between electronic effects and antifungal activity, the electrostatic potential (ESP) distributions of the target compounds **5q** (R = *m*-OH C_6_H_4_) and **5c** (R = *m*-CH_3_ C_6_H_4_) were calculated. The results are shown in Figure 3.

Research has found that **5q** (R = *m*-OH C_6_H_4_) exhibited a *p-π* conjugation effect, which was a very strong electron-donating effect, while **5c** (R = *m*-CH_3_ C_6_H_4_) exhibited *σ-π* hyperconjugation, which was a relatively weak electron-donating group. From the figure, it can be seen that the maximum electrostatic potential of compound **5c** (R = *m*-CH_3_ C_6_H_4_) was 4.141 × 10^−2^, and that of compound **5q** (R = *m*-OH C_6_H_4_) was 5.040 × 10^−2^. As the electron-donating ability increased, the positive charge on the benzene ring decreased, which was consistent with the order of antifungal activity of the two compounds against the tested fungi. In the electrostatic potential map, the negative charge centers represented in blue underwent significant changes. The regions characterized by these negative charge centers exhibited a preferential affinity for electrophilic reagents, thereby showing high potential as active sites. Overall, this indicated that electrostatic effects were an important factor influencing the antifungal activity of the target compound.

**Figure 4:**
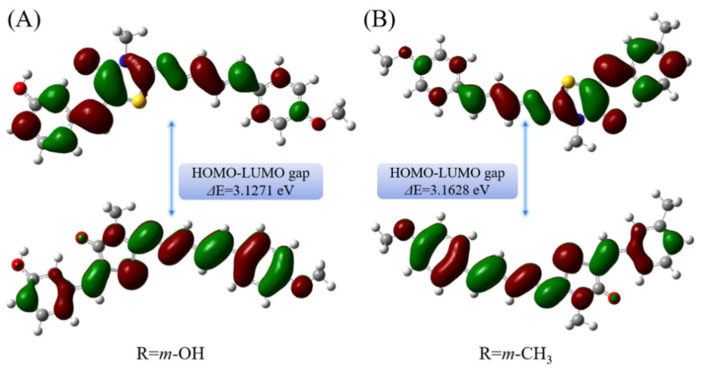
Frontier molecular orbitals of the compounds: HOMO and LUMO of **5q** (R = *m*-OH C_6_H_4_) (**A**); HOMO and LUMO of **5c** (R = *m*-CH_3_ C_6_H_4_) (**B**).

In addition, according to frontier molecular orbital theory (FMO), the highest occupied molecular orbital (HOMO) and the lowest unoccupied molecular orbital (LUMO) are the most important factors influencing biological activity [33,34]. The HOMO and the LUMO serve as the electron donor and acceptor, respectively. HOMO preferentially donates electrons, while LUMO easily accepts electrons. Therefore, using Gaussian 09 on the high-performance computing platform of Guangxi University, the HOMO and LUMO of **5c** (R = *m*-CH_3_ C_6_H_4_) with a weak electron-donating group and **5q** (R = *m*-OH C_6_H_4_) with a strong electron-donating group at the same substitution position on the benzene ring were calculated, and the results shown in Figure 4 were viewed using GaussView 5 software.

As shown in Figure 4, the energy gap (ΔE = 3.1271 eV) between the HOMO and LUMO orbitals of the target compound **5q** (R = *m*-OH C_6_H_4_) was smaller than that of the target compound **5c** (R = *m*-CH_3_ C_6_H_4_) (ΔE = 3.1628 eV), indicating that **5q** (R = *m*-OH C_6_H_4_) had a higher chemical reactivity. At the same time, the smaller energy gap in the molecule suggests it had potential biological activity [35]. This further proved that introducing strong electron-donating groups at the *meta* positions of the benzene ring helped to enhance the biological activity of molecules. Moreover, the LUMO and HOMO of these compounds were mainly distributed on the hydrazone group, thiazolinone ring, anethole group, and substituted benzene ring, which suggested that these active groups may make a major contribution to antifungal activity. This result helps further design and study these novel compounds.

### 2.4. Molecular Docking

The compound **5q** (R = *m*-OH C_6_H_4_), which had the strongest antifungal activity, was selected as a representative compound for a molecular docking study with SDH. For comparative analysis, the positive controls boscalid and **ref3a** (**ref3a** was our previous work) [30] were used as reference compounds.

**Figure 5:**
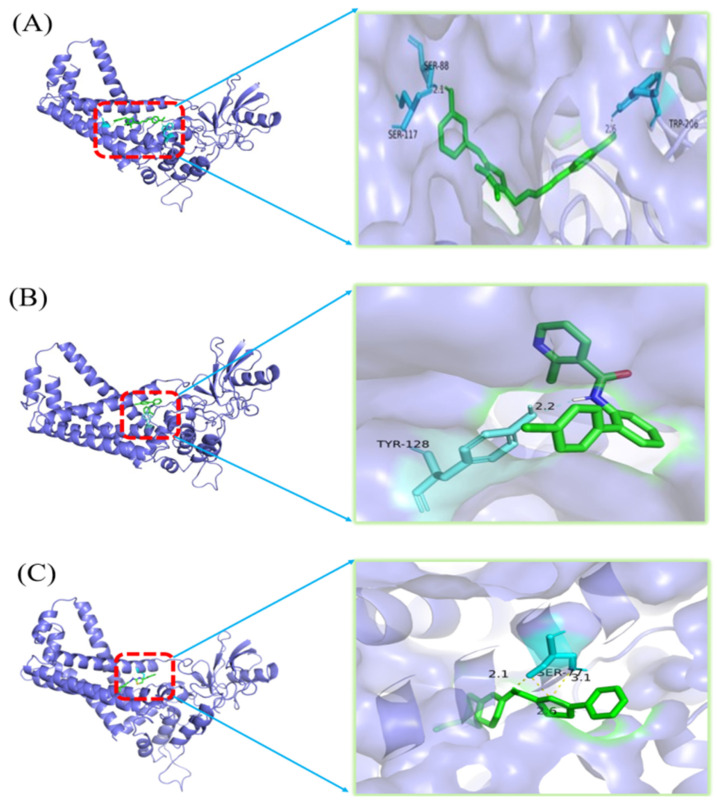
(**A**) **5q** (R = *m*-OH C_6_H_4_), (**B**) commercial SDHI boscalid, and (**C**) **ref3a**, respectively, with the binding pocket of SDH and the three-dimensional interaction mode with the receptor protein.

**Figure 6:**
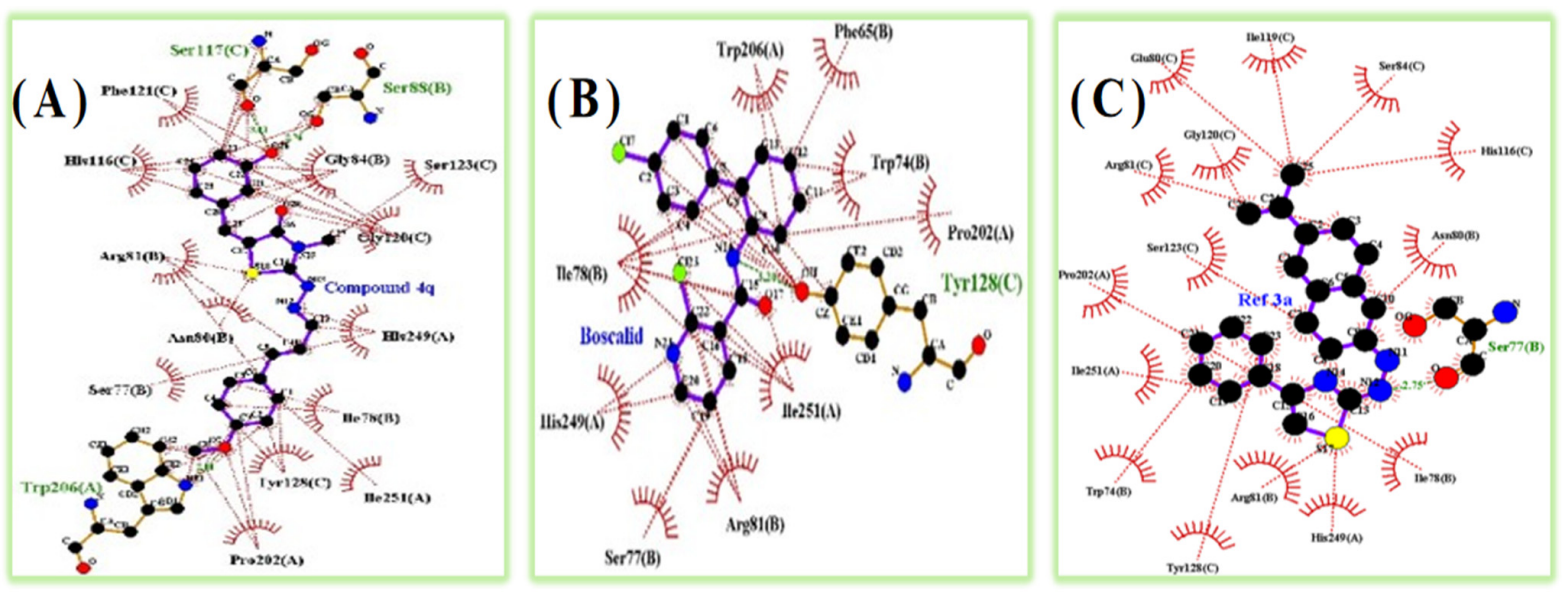
(**A**) **5q** (R = *m*-OH C_6_H_4_), (**B**) commercial SDHI boscalid, (**C**) **ref3a**, two-dimensional interaction pattern with succinate dehydrogenase.

As shown in Figure 5 and Figure 6, molecular docking results indicated that **5q** (R = *m*-OH C_6_H_4_), boscalid, and **ref3a** can all bind within the active pocket of SDH and form multiple interactions with key amino acid residues, suggesting that these molecules may have similar modes of action. For example, all three compounds were surrounded by residues C/Tyr128, B/Arg81, A/His249, A/Pro202, B/Ile78, and B/Ser77, forming Van der Waals contacts. In addition, the binding energies of compound **5q** (R = *m*-OH C_6_H_4_), the positive control boscalid, and **ref3a** were −7.16 kcal/mol, −6.91 kcal/mol, and −10.01 kcal/mol, respectively. The better affinity of compound **5q** (R = *m*-OH C_6_H_4_) with **ref3a** indicated that the addition of the aromatic ring and the resulting new electron distribution promote the increase in affinity [36,37]. In addition, the anethole of the parent scaffold was deeply inserted into the active pocket, where it interacted with key amino acid residues via Van der Waals forces, π-π stacking, and other non-covalent interactions. The introduced thiazolinone ring, hydrazone group, and phenyl ring also displayed similar interaction patterns with SDH. These molecular docking results were consistent with the aforementioned DFT theoretical calculations. Therefore, the introduction of the thiazolinone ring, hydrazone, and phenyl structure may contribute to enhancing the antifungal activity of the anethole-based derivative. The above results suggested that the representative **5q** (R = *m*-OH C_6_H_4_) may have a mechanism of action similar to that of the SDH-targeting inhibitors boscalid and **ref3a**.

## 3. Experimental Section

### 3.1. Materials

(*E*)-Anethole (GC purity 98%) and substituted benzaldehydes were purchased from Shanghai Tansoole Bio Co., Ltd. (Shanghai, China). Ethyl bromoacetate (98%) and sodium ethoxide were supplied by Aladdin Reagents (Shanghai, China). Other analytical grade reagents, anhydrous ethanol, petroleum ether, and ethyl acetate were purchased from commercial suppliers and used as received. Thin-layer chromatography (TLC) was performed on pre-coated silica gel GF254 plates (2.5 × 7.5 cm, 0.25 mm thickness, Qingdao Haiyang Chemical Co., Ltd., Qingdao, China)

### 3.2. Synthesis of Intermediate (***3***)

4-Methylaminothiourea (2.32 g, 22 mmol) was dissolved in 50 mL ethanol at first. Then, (*E*)-4-methoxycinnamaldehyde (3.24 g, 20 mmol) was added to the solution, followed by adding 2–3 mL 5% hydrochloric acid to initiate the condensation reaction. The mixture was stirred under reflux for 8–12 h, and the reaction progress was monitored by TLC. After completion, the solvent was removed under reduced pressure. The crude product was further purified by column chromatography [*V*(petroleum ether): *V*(ethyl acetate) = 10:1 to 2:1] to give intermediate **3** as a yellow solid in 71.8% yield. m.p. 171.7–172.6 °C. ^1^H NMR (600 MHz, DMSO-*d*_6_) *δ*: 11.36 (s, 1H, N-H), 8.20 (d, *J* = 4.5 Hz, 1H, C_10_-H), 7.87 (d, *J* = 9.4 Hz, 1H, N-H), 7.49 (d, *J* = 8.7 Hz, 2H, Ar-H, C_8_-H), 6.99–6.90 (m, 3H, Ar-H), 6.72 (dd, *J* = 16.1, 9.4 Hz, 1H, C_9_-H), 3.77 (s, 3H, C_7_-H), 2.98 (d, *J* = 4.6 Hz, 3H, C_12_-H); ^13^C NMR (151 MHz, DMSO-*d*_6_) *δ*: 177.7, 160.3, 145.0, 138.8, 129.0, 128.8, 123.1, 114.8, 55.6, 31.2.

### 3.3. Synthesis of Intermediate (***4***)

At room temperature, intermediate **3** (8.82 g, 35.4 mmol) was mixed and stirred with sodium ethoxide (2.53 g, 37.2 mmol) in anhydrous ethanol (100 mL), then ethyl bromoacetate (6.49 g, 38.9 mmol) was added to promote intermolecular cyclization. The reaction mixture was then heated under reflux for 6 h, cooled to room temperature, and filtered. The resulting precipitate was washed three times with anhydrous ethanol to obtain intermediate **4** as a yellow solid in 86.0% yield. m.p. 182.4–183.9 °C; ^1^H NMR (600 MHz, CDCl_3_) *δ*: 8.20 (dd, *J* = 5.9, 3.1 Hz, 1H, C_10_-H), 7.44 (d, *J* = 8.7 Hz, 2H, C_9_-H, C_8_-H), 6.97–6.91 (m, 2H, Ar-H), 6.89 (d, *J* = 8.7 Hz, 2H, Ar-H), 3.82 (s, 3H, C^7^-H), 3.77 (s, 2H, C_13_-H), 3.28 (s, 3H, C_14_-H); ^13^C NMR (151 MHz, CDCl_3_) *δ* 172.0, 162.5, 160.9, 160.5, 141.4, 128.8, 128.7 (d, *J* = 15.7 Hz), 123.2, 114.31, 55.38, 32.52, 29.80.

### 3.4. Synthesis of Anethole-Based Thiazolinone-Hydrazone Compounds ***5a***–***5v***

Intermediate **4** (1.67 g, 6.00 mmol), KOH (0.34 g, 6.06 mmol), and different substituted benzaldehydes (7.00 mmol) were mixed and dissolved in anhydrous ethanol for the Knoevenagel condensation reaction. The mixture was stirred under reflux conditions for 4 h, and the reaction progress was monitored by TLC. After the reaction was complete, the reaction solution was cooled to room temperature, resulting in the formation of a yellow precipitate. The solid was then collected by filtration, thoroughly washed with anhydrous ethanol, and dried to afford the target compounds **5a**–**5v** as yellow solid, with yields ranging from 67.3% to 88.4%.

Compound(**5a**): *(Z)-5-((Z)-Benzylidene)-2-(((1E, 2E)-3-(4-methoxyphenyl) allylidene) hydrazineylidene)-3-methylthiazolidin-4-one*. Yellow solid; yield 83.7%; m.p. 189.4–190.3 °C; FT-IR (KBr, cm^−1^): 3072 (Ar-H, C=C-H), 2954, 2833 (C-H), 1706 (C=O), 1627, 1589, 1554, 1510, 1429 (C=C, C=N, Ar), 1243 (C-O-C), 691 (C-S-C); ^1^H NMR (600 MHz, CDCl_3_) *δ*: 8.26 (dd, *J* = 7.1, 1.9 Hz, 1H, C_10_-H), 7.71 (s, 1H, C_15_-H), 7.59 (d, *J* = 7.4 Hz, 2H, C_8_-H, C_9_-H), 7.46 (t, *J* = 8.0 Hz, 4H, Ar-H), 7.39 (d, *J* = 7.4 Hz, 1H, Ar-H), 6.98 (d, *J* = 7.3 Hz, 2H, Ar-H), 6.92–6.89 (m, 2H, Ar-H), 3.84 (s, 3H, C_7_-H), 3.43 (s, 3H, C_14_-H); ^13^C NMR (151 MHz, CDCl_3_) *δ* 166.9, 161.3, 160.6, 158.6, 141.9, 133.9, 130.5, 130.1, 129.7, 128.9 (*J* = 16.7 Hz), 128.7, 123.0, 122.0, 114.3, 55.3, 29.8; HRMS (APCI): calcd for C_21_H_20_N_3_O_2_S [M+H]^+^ 378.1271, found 378.1267.

Compound(**5b**): *(Z)-2-(((1E, 2E)-3-(4-Methoxyphenyl) allylidene) hydrazineylidene)-3-methyl-5-((Z)-2-methylbenzylidene) thiazolidin-4-one.* Yellow solid; yield 86.2%; m.p. 161.5–162.4 °C; FI-IR (KBr, cm^−1^): 3058 (Ar-H, C=C-H), 2933, 2845 (C-H), 1712 (C=O), 1606, 1569, 1516, 1425 (C=C, C=N, Ar), 1258 (C-O-C), 770 (C-S-C); ^1^H NMR (600 MHz, CDCl_3_) *δ* 8.26 (t, J = 4.5 Hz, 1H, C_10_-H), 7.93 (s, 1H, C_15_-H), 7.65–7.62 (m, 1H, C_8_-H), 7.48–7.46 (m, 2H, C_9_-H, Ar-H), 7.31–7.27 (m, 3H, Ar-H), 6.97 (d, J = 4.9 Hz, 2H, Ar-H), 6.93–6.91 (m, 2H, Ar-H), 3.85 (s, 3H, C_7_-H), 3.45 (s, 3H, C_14_-H), 2.46 (s, 3H, C_22_-H); ^13^C NMR (151 MHz, CDCl_3_) *δ* 166.7, 161.2, 160.6, 158.9, 141.9, 138.6, 133.0, 130.8, 129.6, 128.8, 128.7, 128.4, 127.8, 126.3, 123.3, 123.0, 114.3, 55.3, 29.7, 20.0; HRMS (APCI): calcd. for C_22_H_22_N_3_O_2_S [M+H]^+^ 392.1427, found 392.1423.

Compound(**5c**): *(Z)-2-(((1E, 2E)-3-(4-Methoxyphenyl) allylidene) hydrazineylidene)-3-methyl-5-((Z)-3-methylbenzylidene) thiazolidin-4-one*. Yellow solid; yield 82.4%; m.p. 177.5–178.7 °C; FT-IR (KBr, cm^−1^): 3070 (Ar-H, C=C-H), 2995, 2839 (C-H), 1725 (C=O), 1627, 1597, 1510, 1425 (C=C, C=N, Ar), 1254 (C-O-C), 683 (C-S-C); ^1^H NMR (600 MHz, CDCl_3_) *δ* 8.26 (d, *J* = 8.5 Hz, 1H, C_10_-H), 7.68 (s, 1H, C_15_-H), 7.47 (d, *J* = 8.7 Hz, 2H, C_8_-H, C_9_-H), 7.42–7.38 (m, 2H, Ar-H), 7.34 (t, *J* = 7.6 Hz, 1H, Ar-H), 7.20 (d, *J* = 7.5 Hz, 1H, Ar-H), 6.99 (dd, *J* = 18.5, 10.0 Hz, 2H, Ar-H), 6.91 (d, *J* = 8.7 Hz, 2H, Ar-H), 3.84 (s, 3H, C_7_-H), 3.43 (s, 3H, C_14_-H), 2.42 (s, 3H, C_22_-H); ^13^C NMR (151 MHz, CDCl_3_) *δ* 166.9, 161.3, 160.6, 158.6, 141.9, 138.7, 133.9, 130.9, 130.7, 130.5, 129.6, 128.9, 128.7, 127.4, 123.0, 121.6, 114.3, 55.3, 29.8, 21.4; HRMS (APCI): calcd. for C_22_H_22_N_3_O_2_S [M+H]^+^ 392.1427, found 392.1420.

Compound(**5d**): *(Z)-2-(((1E, 2E)-3-(4-Methoxyphenyl) allylidene) hydrazineylidene)-3-methyl-5-((Z)-4-methylbenzylidene) thiazolidin-4-one*. Yellow solid; yield 85.3%; m.p. 221.3–223.4 °C; FT-IR (KBr, cm^−1^): 3062 (Ar-H, C=C-H), 2995, 2841 (C-H), 1712 (C=O), 1629, 1604, 1565, 1516, 1431 (C=C, C=N, Ar), 1254 (C-O-C), 706 (C-S-C); ^1^H NMR (600 MHz, CDCl_3_) *δ* 8.26 (dd, *J* = 8.0, 0.9 Hz, 1H, C_10_-H), 7.69 (s, 1H, C_15_-H), 7.51–7.45 (m, 4H, Ar-H), 7.27 (s, 1H, C_8_-H), 7.25 (s, 1H, C_9_-H), 7.02–6.96 (m, 2H, Ar-H), 6.93–6.89 (m, 2H, Ar-H), 3.84 (s, 3H, C_7_-H), 3.43 (s, 3H, C_14_-H), 2.40 (s, 3H, C_22_-H); ^13^C NMR (151 MHz, CDCl_3_) *δ*167.0, 161.2, 160.6, 158.7, 141.8, 140.2, 131.1, 130.7, 130.2, 129.7, 128.9, 128.7, 123.1, 120.7, 114.3, 55.3, 29.8, 21.5; HRMS (APCI): calcd. For. C_22_H_22_N_3_O_2_S [M+H]^+^ 392.1427, found 392.1422.

Compound(**5e**): *(Z)-5-((Z)-2-Methoxybenzylidene)-2-(((1E, 2E)-3-(4-methoxyphenyl) allylidene) hydrazineylidene)-3-methylthiazolidin-4-one*. Yellow solid; yield 87.3%; m.p. 199.4–200.3 °C; FT-IR (KBr, cm^−1^): 3043 (Ar-H, C=C-H), 2954, 2835 (C-H), 1708 (C=O), 1629, 1597, 1516 (C=C, C=N, Ar), 1245 (C-O-C), 750 (C-S-C); ^1^H NMR (600 MHz, CDCl_3_) *δ* 8.25 (dd, *J* = 7.4, 1.6 Hz, 1H, C_10_-H), 8.14 (s, 1H, C_15_-H), 7.63 (dd, *J* = 7.7, 1.3 Hz, 1H, C_8_-H), 7.46 (t, *J* = 5.8 Hz, 2H, C_9_-H, Ar-H), 7.38–7.34 (m, 1H, Ar-H), 7.04 (t, *J* = 7.5 Hz, 1H, Ar-H), 6.98–6.89 (m, 5H, Ar-H), 3.89 (s, 3H, C_7_-H), 3.84 (s, 3H, C_22_-H), 3.43 (s, 3H, C_14_-H); ^13^C NMR (151 MHz, CDCl_3_) *δ* 166.9, 161.0, 160.6, 159.0, 158.3, 141.7, 131.3, 129.1, 128.8 (*J* = 19.1 Hz), 125.8, 123.1, 121.8, 120.6, 114.3, 110.9, 55.5, 55.3, 29.7; HRMS (APCI): calcd. for C_22_H_22_N_3_O_3_S [M+H]^+^ 408.1376, found 408.1370.

Compound(**5f**): *(Z)-5-((Z)-3-Methoxybenzylidene)-2-(((1E, 2E)-3-(4-methoxyphenyl) allylidene) hydrazineylidene)-3-methylthiazolidin-4-one*. Yellow solid; yield 88.1%; m.p. 169.8–170.7 °C; FT-IR (KBr, cm^−1^): 3006 (Ar-H, C=C-H), 2954, 2843 (C-H), 1714 (C=O), 1633, 1602,1558, 1510, 1431 (C=C, C=N, Ar), 1258 (C-O-C), 679 (C-S-C); ^1^H NMR (600 MHz, CDCl_3_) *δ* 8.25 (dd, *J* = 7.1, 1.8 Hz, 1H, C_10_-H), 7.68 (s, 1H, C_15_-H), 7.48–7.45 (m, 2H, C_8_-H, C_9_-H), 7.37 (t, *J* = 7.9 Hz, 1H, Ar-H), 7.20 (d, *J* = 7.7 Hz, 1H, Ar-H), 7.10–7.08 (m, 1H, Ar-H), 6.98 (d, *J* = 7.3 Hz, 2H, Ar-H), 6.95–6.93 (m, 1H, Ar-H), 6.92–6.89 (m, 2H, Ar-H), 3.87 (s, 3H, C_7_-H), 3.84 (s, 3H, C_22_-H), 3.43 (s, 3H, C_14_-H); ^13^C NMR (151 MHz, CDCl_3_) *δ* 166.8, 161.3, 160.6, 159.9, 158.5, 141.9, 135.2, 130.5, 130.0, 128.9, 128.7, 123.0, 122.5, 122.4, 115.5, 115.2, 114.3, 55.4 (*J* = 6.9 Hz), 29.8; HRMS (APCI): calcd. for C_22_H_22_N_3_O_3_S [M+H]^+^ 408.1376, found 408.1371.

Compound(**5g**): *(Z)-5-((Z)-4-Methoxybenzylidene)-2-(((1E, 2E)-3-(4-methoxyphenyl) allylidene) hydrazineylidene)-3-methylthiazolidin-4-one*. Yellow solid; yield 88.4%; m.p. 219.6–220.3 °C; FT-IR (KBr, cm^−1^): 3052 (Ar-H, C=C-H), 3004, 2837 (C-H), 1702 (C=O), 1625, 1604, 1560, 1510, 1429 (C=C, C=N, Ar), 1254 (C-O-C), 712 (C-S-C); ^1^H NMR (600 MHz, CDCl_3_) *δ* 8.26 (dd, *J* = 7.9, 0.9 Hz, 1H, C_10_-H), 7.67 (s, 1H, C_15_-H), 7.57–7.54 (m, 2H, C_8_-H, C_9_-H), 7.48–7.45 (m, 2H, Ar-H), 6.98 (dd, *J* = 7.0, 1.9 Hz, 4H, Ar-H), 6.92–6.89 (m, 2H, Ar-H), 3.86 (s, 3H, C_7_-H), 3.84 (s, 3H, C_22_-H), 3.43 (s, 3H, C_14_-H); ^13^C NMR (151 MHz, CDCl_3_) *δ* 167.1, 161.0, 160.8, 160.6, 158.9, 141.7, 132.0, 130.4, 128.8, 128.7, 126.6, 123.1, 119.1, 114.5, 114.3, 55.4 (*J* = 9.3 Hz), 29.8; HRMS (APCI): calcd. for C_22_H_22_N_3_O_3_S [M+H]^+^ 408.1376, found 408.1372.

Compound(**5h**): *(Z)-5-((Z)-2-Fluorobenzylidene)-2-(((1E, 2E)-3-(4-methoxyphenyl) allylidene) hydrazineylidene)-3-methylthiazolidin-4-one*. Yellow solid; yield 80.2%; m.p. 188.1–188.9 °C; FT-IR (KBr, cm^−1^): 3014 (Ar-H, C=C-H), 2933, 2833 (C-H), 1710 (C=O), 1627, 1604, 1545, 1508 (C=C, C=N, Ar), 1243 (C-O-C), 764 (C-S-C); ^1^H NMR (600 MHz, CDCl_3_) *δ* 8.26 (dd, J = 5.4, 3.6 Hz, 1H, C_10_-H), 7.94 (s, 1H, C_15_-H), 7.68 (td, *J* = 7.7, 1.4 Hz, 1H, C_8_-H), 7.47–7.45 (m, 2H, C_9_-H, Ar-H), 7.37 (tdd, *J* = 8.1, 5.2, 1.6 Hz, 1H, Ar-H), 7.25 (t, *J* = 7.7 Hz, 1H, Ar-H), 7.15–7.11 (m, 1H, Ar-H), 6.98–6.96 (m, 2H, Ar-H), 6.92–6.89 (m, 2H, Ar-H), 3.84 (s, 3H, C_7_-H), 3.44 (s, 3H, C_14_-H); ^13^C NMR (151 MHz, CDCl_3_) *δ* 166.5, 162.1 (d, *J* = 253.6 Hz) 161.5, 160.7, 158.2, 142.1, 131.4 (d, *J* = 8.7 Hz), 129.1, 128.9, 128.6, 124.4 (d, *J* = 3.8 Hz), 124.0, 122.9, 122.2 (*J* = 24.5, 9.3 Hz), 116.0, 115.9, 114.3, 55.3, 29.9; HRMS (APCI): calcd. for C_21_H_19_FN_3_O_2_S [M+H]^+^ 396.1177, found 396.1171.

Compound(**5i**): *(Z)-5-((Z)-4-Fluorobenzylidene)-2-(((1E, 2E)-3-(4-methoxyphenyl) allylidene) hydrazineylidene)-3-methylthiazolidin-4-one*. Yellow solid; yield 83.2%; m.p. 211.3–212.6 °C; FT-IR (KBr, cm^−1^): 3060 (Ar-H, C=C-H), 2950, 2839 (C-H), 1706 (C=O), 1635, 1602, 1508, 1431 (C=C, C=N, Ar), 1256 (C-O-C), 710 (C-S-C); ^1^H NMR (600 MHz, CDCl_3_) *δ* 8.25 (dd, *J* = 5.0, 4.0 Hz, 1H, C_10_-H), 7.66 (s, 1H, C_15_-H), 7.59–7.56 (m, 2H, C_8_-H, C_9_-H), 7.48–7.44 (m, 2H, Ar-H), 7.17–7.12 (m, 2H, Ar-H), 6.99–6.96 (m, 2H, Ar-H), 6.93–6.89 (m, 2H, Ar-H), 3.84 (s, 3H, C_7_-H), 3.43 (s, 3H, C_14_-H); ^13^C NMR (151 MHz, CDCl_3_) *δ* 166.8, 163.9 (d, J = 252.1 Hz), 161.4, 160.7, 158.3, 142.1, 132.1 (d, *J* = 8.4 Hz), 130.2 (d, *J* = 3.3 Hz), 129.2, 128.9, 128.6, 122.9, 121.7 (d, *J* = 2.4 Hz), 116.1, 114.3, 55.3, 29.9; HRMS (APCI): calcd. for C_21_H_19_FN_3_O_2_S [M+H]^+^ 396.1177, found 396.1172.

Compound(**5j**): *(Z)-5-((Z)-2-Chlorobenzylidene)-2-(((1E, 2E)-3-(4-methoxyphenyl) allylidene) hydrazineylidene)-3-methylthiazolidin-4-one*. Yellow solid; yield 78.5%; m.p. 188.2–189.3 °C; FT-IR (KBr, cm^−1^): 3064 (Ar-H, C=C-H), 2929, 2839 (C-H), 1718 (C=O), 1674, 1635, 1510, 1429 (C=C, C=N, Ar), 1258 (C-O-C), 760 (C-S-C); ^1^H NMR (600 MHz, CDCl_3_) *δ* 8.25 (dd, *J* = 7.5, 1.4 Hz, 1H, C_10_-H), 8.06 (s, 1H, C_15_-H), 7.70 (dd, J = 7.7, 1.4 Hz, 1H, C_8_-H), 7.46 (dd, *J* = 10.4, 4.9 Hz, 3H, C_9_-H, Ar-H), 7.34 (ddd, J = 9.2, 7.5, 1.1 Hz, 2H, Ar-H), 6.95 (d, J = 7.5 Hz, 2H, Ar-H), 6.90 (t, J = 5.7 Hz, 2H, Ar-H), 3.83 (s, 3H, C_7_-H), 3.44 (s, 3H, C_14_-H); ^13^C NMR (151 MHz, CDCl_3_) *δ* 166.3, 161.5, 160.7, 158.2, 142.1, 135.6, 132.3, 130.5, 130.2, 129.2, 128.9, 128.6, 127.1, 126.6, 125.0, 122.9, 114.3, 55.3, 29.9; HRMS (APCI): calcd. for C_21_H_19_ClN_3_O_2_S [M+H]^+^ 412.0881, found 412.0875.

Compound(**5k**): *(Z)-5-((Z)-3-Chlorobenzylidene)-2-(((1E, 2E)-3-(4-methoxyphenyl) allylidene) hydrazineylidene)-3-methylthiazolidin-4-one*. Yellow solid; yield 77.8%; m.p. 203.1–204.7 °C; FT-IR (KBr, cm^−1^): 3075 (Ar-H, C=C-H), 2935, 2847 (C-H), 1710 (C=O), 1637, 1593, 1512, 1429 (C=C, C=N, Ar), 1250 (C-O-C), 687 (C-S-C); ^1^H NMR (600 MHz, CDCl_3_) *δ* 8.27 (dd, J = 6.6, 2.4 Hz, 1H, C_10_-H), 7.63 (s, 1H, C_15_-H), 7.56 (s, 1H, C_8_-H), 7.49–7.46 (m, 3H, C_9_-H, Ar-H), 7.37 (ddd, *J* = 11.0, 8.1, 4.7 Hz, 2H, Ar-H), 7.02–6.97 (m, 2H, Ar-H), 6.91 (d, *J* = 8.7 Hz, 2H, Ar-H), 3.84 (s, 3H, C_7_-H), 3.44 (s, 3H, C^14^-H); ^13^C NMR (151 MHz, CDCl_3_) *δ* 166.6, 161.6, 160.7, 158.0, 142.2, 135.7, 135.0, 130.2, 129.7, 129.5, 128.9, 128.7, 128.6, 128.1, 123.7, 122.9, 114.3, 55.4, 29.9; HRMS (APCI): calcd. for C_21_H_19_ClN_3_O_2_S [M+H]^+^ 412.0881, found 412.0876.

Compound(**5l**): *(Z)-5-((Z)-4-Chlorobenzylidene)-2-(((1E, 2E)-3-(4-methoxyphenyl) allylidene) hydrazineylidene)-3-methylthiazolidin-4-one*. Yellow solid; yield 74.9%; m.p. 212.7–213.5 °C; FT-IR (KBr, cm^−1^): 3014 (Ar-H, C=C-H), 2933, 2839 (C-H), 1710 (C=O), 1631, 1604, 1506, 1433 (C=C, C=N, Ar), 1260(C=O), 683 (C-S-C); ^1^H NMR (600 MHz, CDCl_3_) *δ* 8.27–8.22 (m, 1H, C_10_-H), 7.63 (s, 1H, C_15_-H), 7.50 (dd, *J* = 6.2, 4.5 Hz, 2H, C_8_-H, C_9_-H), 7.48–7.44 (m, 2H, Ar-H), 7.43–7.38 (m, 2H, Ar-H), 6.98–6.94 (m, 2H, Ar-H), 6.92–6.88 (m, 2H, Ar-H), 3.84 (s, 3H, C_7_-H), 3.43 (s, 3H, C_14_-H); ^13^C NMR (151 MHz, CDCl_3_) *δ* 166.7, 161.5, 160.7, 158.1, 142.1, 135.6, 132.4, 131.2, 129.2, 128.9 (*J* = 4.7 Hz), 128.6, 122.9, 122.7, 114.3, 55.3, 29.9; HRMS (APCI): calcd. for C_21_H_19_ClN_3_O_2_S [M+H]^+^ 412.0881, found 412.0875.

Compound(**5m**): *(Z)-5-((Z)-2-Bromobenzylidene)-2-(((1E, 2E)-3-(4-methoxyphenyl) allylidene) hydrazineylidene)-3-methylthiazolidin-4-one*. Yellow solid; yield 80.4%; m.p. 188.1–189.6 °C; FT-IR (KBr, cm^−1^): 3035 (Ar-H, C=C-H), 2941, 2839 (C-H), 1708 (C=O), 1637, 1595, 1516, 1427 (C=C, C=N, Ar), 1254 (C-O-C), 662 (C-S-C); ^1^H NMR (600 MHz, CDCl_3_) *δ* 8.27 (dd, J = 8.0 Hz, 1H, C_10_-H), 8.02 (s, 1H, C_15_-H), 7.69 (m, *J* = 10.9, 7.9, 1.2 Hz, 2H, Ar-H), 7.47 (t, *J* = 5.8 Hz, 2H, Ar-H), 7.42 (t, *J* = 7.6 Hz, 1H, C_8_-H), 7.24 (td, *J* = 7.8, 1.5 Hz, 1H, C_9_-H), 6.98 (dd, *J* = 13.5, 11.7 Hz, 2H, Ar-H), 6.94–6.90 (m, 2H, Ar-H), 3.85 (s, 3H, C^7^-H), 3.46 (s, 3H, C_14_-H); ^13^C NMR (151 MHz, CDCl_3_) *δ* 166.2, 161.5, 160.7, 158.2, 142.1, 134.1, 133.5, 130.6, 129.2, 128.9, 128.6, 127.7, 125.9, 125.2, 123.4, 122.9, 114.3, 55.3, 29.9; HRMS (APCI): calad. For C_21_H_19_BrN_3_O_2_S [M+H]^+^ 456.0376, 458.0355, found 456.0371, 458.0349.

Compound(**5n**): *(Z)-5-((Z)-3-Bromobenzylidene)-2-(((1E, 2E)-3-(4-methoxyphenyl) allylidene) hydrazineylidene)-3-methylthiazolidin-4-one*. Yellow solid; yield 77.2%; m.p. 196.8–197.4 °C; FT-IR (KBr, cm^−1^): 3058 (Ar-H, C=C-H), 2943, 2845 (C-H), 1708 (C=O), 1631, 1597, 1550, 1512 (C=C, C=N, Ar), 1256 (C-O-C), 683 (C-S-C); ^1^H NMR (600 MHz, CDCl_3_) *δ* 8.26 (dd, J = 6.9, 2.1 Hz, 1H, C_10_-H), 7.70 (t, J = 1.7 Hz, 1H, C_15_-H), 7.61 (s, 1H, C_8_-H), 7.52–7.46 (m, 4H, Ar-H), 7.32 (t, J = 7.9 Hz, 1H, C_9_-H), 7.02–6.96 (m, 2H, Ar-H), 6.93–6.89 (m, 2H, Ar-H), 3.84 (s, 3H, C_7_-H), 3.44 (s, 3H, C_14_-H); ^13^C NMR (151 MHz, CDCl_3_) *δ* 166.6, 161.6, 160.7, 157.9, 142.2, 136.0, 132.6, 132.4, 130.4, 128.9, 128.6 (*J* = 3.2 Hz), 128.4, 123.7, 123.1, 122.9, 114.3, 55.4, 29.9; HRMS (APCI): calad. For C_21_H_19_BrN_3_O_2_S [M+H]^+^ 456.0376, 458.0355, found 456.0370, 458.0348.

Compound(**5o**): *(Z)-5-((Z)-4-Iodobenzylidene)-2-(((1E, 2E)-3-(4-methoxyphenyl) allylidene) hydrazineylidene)-3-methylthiazolidin-4-one*. Yellow solid; yield 76.3%; m.p. 223.2–224.9 °C; FT-IR (KBr, cm^−1^): 3012 (Ar-H, C=C-H), 2960, 2841 (C-H), 1710 (C=O), 1635, 1600, 1514,1420 (C=C, C=N, Ar), 1260 (C-O-C), 662 (C-S-C); ^1^H NMR (600 MHz, CDCl_3_) *δ* 8.27 (dd, *J* = 5.5, 3.5 Hz, 1H, C_10_-H), 7.79 (d, *J* = 8.4 Hz, 2H, C_15_-H, C_8_-H), 7.61 (s, 1H, C_9_-H), 7.50–7.45 (m, 2H, Ar-H), 7.31 (d, *J* = 8.3 Hz, 2H, Ar-H), 7.01–6.97 (m, 2H, Ar-H), 6.94–6.90 (m, 2H, Ar-H), 3.85 (s, 3H, C_7_-H), 3.44 (s, 3H, C_8_-H); ^13^C NMR (151 MHz, CDCl_3_) *δ* 166.7, 161.5, 160.7, 158.0, 142.2, 138.2, 133.3, 131.4, 129.1, 128.9, 128.6, 122.9 (*J* = 12.5 Hz), 114.3, 96.0, 55.4, 29.9; HRMS (APCI): calcd. for C_21_H_19_IN_3_O_2_S [M+H]^+^ 504.0237, found 504.0233.

Compound(**5p**): *(Z)-2-(((1E, 2E)-3-(4-Methoxyphenyl) allylidene) hydrazineylidene)-3-methyl-5-((Z)-3-(trifluoromethyl) benzylidene) thiazolidin-4-one*. Yellow solid; yield 87.1%; m.p. 177.8–179.1 °C; FT-IR (KBr, cm^−1^): 3002 (Ar-H, C=C-H), 2943, 2837 (C-H), 1710 (C=O), 1633, 1602, 1512, 1427, 1345 (C=C, C=N, Ar), 1125 (C-O-C), 691 (C-S-C); ^1^H NMR (600 MHz, CDCl_3_) *δ* 8.26 (dd, *J* = 6.4, 2.6 Hz, 1H, C_10_-H), 7.80–7.76 (m, 2H, C_8_-H, Ar-H), 7.70 (s, 1H, C_15_-H), 7.63 (d, *J* = 7.8 Hz, 1H, C_9_-H), 7.59 (t, *J* = 7.7 Hz, 1H, Ar-H), 7.49–7.46 (m, 2H, Ar-H), 7.01–6.97 (m, 2H, Ar-H), 6.93–6.90 (m, 2H, Ar-H), 3.84 (s, 3H, C_7_-H), 3.45 (s, 3H, C_14_-H); ^13^C NMR (151 MHz, CDCl_3_) *δ* 166.5, 161.7, 160.7, 157.7, 142.3, 134.8, 132.6, 131.6 (d, J = 30.2 Hz), 129.5, 128.9, 128.6, 128.4, 126.7 (d, *J* = 3.8 Hz), 125.9 (d, *J* = 3.6 Hz), 124.3, 124.6 (d, J = 271.8 Hz), 122.9, 114.3, 55.3, 30.0; HRMS (APCI): calad. For C_22_H_19_F_3_N_3_O_2_S [M+H]^+^ 446.1145, found 446.1140.

Compound(**5q**): *(Z)-5-((Z)-3-Hydroxybenzylidene)-2-(((1E, 2E)-3-(4-methoxyphenyl) allylidene) hydrazineylidene)-3-methylthiazolidin-4-one*. Yellow solid; yield 72.1%; m.p. 235.4–236.9 °C; FT-IR (KBr, cm^−1^): 3427 (-OH), 2993 (Ar-H, C=C-H), 2933, 2835 (C-H), 1700 (C=O), 1631, 1595, 1510, 1437 (C=C, C=N, Ar), 1256 (C-O-C), 679 (C-S-C); ^1^H NMR (600 MHz, DMSO) *δ* 8.29 (d, *J* = 9.5 Hz, 1H, C_10_-H), 7.61 (d, *J* = 8.7 Hz, 2H, C_8_-H, C_9_-H), 7.48 (s, 1H, C_15_-H), 7.17–7.04 (m, 3H, Ar-H), 6.95 (d, *J* = 8.8 Hz, 2H, Ar-H), 6.84 (s, 1H, Ar-H), 6.70 (d, *J* = 8.1 Hz, 1H, Ar-H), 6.67 (d, *J* = 7.4 Hz, 1H, Ar-H), 3.79 (s, 3H, C_7_-H), 3.28 (s, 3H, C_14_-H); ^13^C NMR (151 MHz, DMSO) *δ* 166.6, 161.3, 160.7, 159.1, 142.7, 134.6, 132.0, 130.0, 129.6, 128.8, 123.3, 120.1, 118.2, 116.7, 114.8, 56.4, 55.7, 30.0, 19.0; HRMS (APCI): calcd. for C_21_H_20_N_3_O_2_S [M+H]^+^ 394.1220, found 394.1216.

Compound(**5r**): *(Z)-5-((Z)-2-Hydroxybenzylidene)-2-(((1E, 2E)-3-(4-methoxyphenyl) allylidene) hydrazineylidene)-3-methylthiazolidin-4-one*. Yellow solid; yield 67.3%; m.p. 247.1–248.4 °C; FT-IR (KBr, cm^−1^): 3429 (-OH), 3058 (Ar-H, C=C-H), 2954, 2839 (C-H), 1685 (C=O), 1631, 1591, 1547, 1516, 1435 (C=C, C=N, Ar), 1258 (C-O-C), 752 (C-S-C); ^1^H NMR (600 MHz, DMSO) *δ* 10.45 (s, 1H, -OH), 8.32 (d, J = 9.6 Hz, 1H, C_10_-H), 7.96 (s, 1H, C_15_-H), 7.62 (d, *J* = 8.8 Hz, 2H, C_8_-H, Ar-H), 7.47 (dd, *J* = 7.7, 1.3 Hz, 1H, C_9_-H), 7.30 (dd, *J* = 11.0, 4.5 Hz, 1H, Ar-H), 7.19 (d, *J* = 15.9 Hz, 1H, Ar-H), 7.06–6.99 (m, 2H, Ar-H), 6.99–6.97 (m, 3H, Ar-H), 3.80 (s, 3H, C_7_-H), 3.32 (s, 3H, C_14_-H); ^13^C NMR (151 MHz, DMSO) *δ* 166.5, 161.5, 160.8, 158.7, 157.5, 142.8, 132.1, 129.6, 128.8, 125.1, 123.1, 121.0, 120.7, 120.0, 116.5, 114.8, 55.7, 30.1; HRMS (APCI): calcd. for C_21_H_20_N_3_O_2_S [M+H]^+^ 394.1220, found 394.1215.

Compound(**5s**): *(Z)-2-(((1E, 2E)-3-(4-Methoxyphenyl) allylidene) hydrazineylidene)-3-methyl-5-((Z)-4-(trifluoromethoxy) benzylidene) thiazolidin-4-one*. Yellow solid; yield 83.1%; m.p. 182.1–183.4 °C; FT-IR (KBr, cm^−1^): 3008 (Ar-H, C=C-H), 2943, 2839 (C-H), 1708 (C=O), 1637, 1600, 1562, 1514, 1427 (C=C, C=N, Ar), 1270 (C-O-C), 614 (C-S-C); ^1^H NMR (600 MHz, CDCl_3_) *δ* 8.26 (dd, *J* = 5.6, 3.4 Hz, 1H, C_10_-H), 7.67 (s, 1H, C_15_-H), 7.62–7.60 (m, 2H, C_8_-H, C_9_-H), 7.48–7.45 (m, 2H, Ar-H), 7.29 (d, J = 8.2 Hz, 2H, Ar-H), 6.98–6.96 (m, 2H, Ar-H), 6.92–6.90 (m, 2H, Ar-H), 3.84 (s, 3H, C_7_-H), 3.44 (s, 3H, C_14_-H); ^13^C NMR (151 MHz, CDCl_3_) *δ* 166.6, 161.5, 160.7, 158.0, 149.6 (d, *J* = 1.6 Hz), 142.2, 132.5, 131.5, 128.9, 128.6 (d, *J* = 1.2 Hz), 123.1, 122.9, 121.2, 119.5 (d, J = 256.7 Hz), 114.3, 55.3, 29.9; HRMS (APCI): calcd. for C_22_H_19_F_3_N_3_O_3_S [M+H]^+^ 462.1094, found 462.1089.

Compound(**5t**): *(Z)-5-((Z)-4-Bromo-3-fluorobenzylidene)-2-(((1E, 2E)-3-(4-methoxphenyl) allylidene) hydrazineylidene)-3-methylthiazolidin-4-one*. Yellow solid; yield 80.3%; m.p. 236.7–237.8 °C; FT-IR (KBr, cm^−1^): 3006 (Ar-H, C=C-H), 2945, 2841 (C-H), 1706 (C=O), 1641, 1604, 1514, 1487, 1429 (C=C, C=N, Ar), 1266 (C-O-C), 679 (C-S-C); ^1^H NMR (600 MHz, CDCl_3_) *δ* 8.27 (dd, *J* = 5.5, 3.5 Hz, 1H, C_10_-H), 7.65–7.59 (m, 2H, C_15_-H, Ar-H), 7.50–7.46 (m, 2H, Ar-H), 7.35 (dd, *J* = 9.5, 2.0 Hz, 1H, C_8_-H), 7.26–7.23 (m, 1H, C_9_-H), 7.02–6.97 (m, 2H, Ar-H), 6.94–6.90 (m, 2H, Ar-H), 3.85 (s, 3H, C_7_-H), 3.45 (s, 3H, C_14_-H); ^13^C NMR (151 MHz, CDCl_3_) *δ* 166.5, 161.8 (d, J = 271.8 Hz), 160.8, 157.6, 142.4, 135.2 (d, J = 7.55 Hz), 134.0, 128.9, 128.5, 127.8 (d, J = 1.51 Hz), 126.7 (d, J = 3.02 Hz), 124.3, 122.8, 117.2, 117.1, 114.3, 110.4, 55.4, 30.0; HRMS (APCI): calcd. for C_21_H_18_BrFN_3_O_2_S [M+H]^+^ 476.0261, 474.0282, found 476.0257, 474.0279.

Compound(**5u**): *(Z)-5-((Z)-2,4-Dimethoxybenzylidene)-2-(((1E, 2E)-3-(4-methoxyphenyl) allylidene) hydrazineylidene)-3-methylthiazolidin-4-one*. Yellow solid; yield 86.2%; m.p. 216.9–217.6 °C; FT-IR (KBr, cm^−1^): 3004 (Ar-H, C=C-H), 2937, 2833 (C-H), 1716 (C=O), 1631, 1606, 1550, 1514, 1422 (C=C, C=N, Ar), 1275 (C-O-C), 716 (C-S-C); ^1^H NMR (600 MHz, CDCl_3_) *δ* 8.25 (d, *J* = 8.2 Hz, 1H, C_10_-H), 8.08 (s, 1H, C_15_-H), 7.57 (d, *J* = 8.6 Hz, 1H, C_8_-H), 7.46 (d, *J* = 8.7 Hz, 2H, C_9_-H, Ar-H), 7.01–6.95 (m, 2H, Ar-H), 6.90 (d, *J* = 8.7 Hz, 2H, Ar-H), 6.57 (dd, *J* = 8.6, 2.3 Hz, 1H, Ar-H), 6.46 (d, *J* = 2.3 Hz, 1H, Ar-H), 3.87 (s, 3H, C_7_-H), 3.86 (s, 3H, C_22_-H), 3.84 (s, 3H, C_23_-H), 3.42 (s, 3H, C_14_-H); ^13^C NMR (151 MHz, CDCl_3_) *δ* 167.2, 162.5, 160.8, 160.5, 159.9, 159.3, 141.5, 130.5, 128.8 (*J* = 7.1 Hz), 125.6, 123.2, 118.7, 116.2, 114.3, 105.1, 98.4, 55.5 (*J* = 11.7 Hz), 55.3, 29.7; HRMS (APCI): calcd. for C_23_H_24_N_3_O_4_S [M+H]^+^ 438.1482, found 438.1477.

Compound(**5v**): *(Z)-2-(((1E, 2E)-3-(4-Methoxyphenyl) allylidene) hydrazineylidene)-3-methyl-5-((Z)-4-(methylthio) benzylidene) thiazolidin-4-one*. Yellow solid; yield 75.4%; m.p. 206.7–207.4 °C; FT-IR (KBr, cm^−1^): 3008 (Ar-H, C=C-H), 2939, 2839 (C-H), 1708 (C=O), 1637, 1600, 1558,1514,1427 (C=C, C=N, Ar), 1260 (C-O-C), 710 (C-S-C); ^1^H NMR (600 MHz, CDCl_3_) *δ* 8.26 (dd, *J* = 6.8, 2.2 Hz, 1H, C_10_-H), 7.65 (s, 1H, C_15_-H), 7.50 (d, *J* = 8.4 Hz, 2H, C_8_-H, C_9_-H), 7.47 (d, *J* = 8.7 Hz, 2H, Ar-H), 7.28 (d, *J* = 8.4 Hz, 2H, Ar-H), 7.01–6.96 (m, 2H, Ar-H), 6.91 (d, *J* = 8.7 Hz, 2H, Ar-H), 3.84 (s, 3H, C_7_-H), 3.43 (s, 3H, C_14_-H), 2.52 (s, 3H, C_22_-H); ^13^C NMR (151 MHz, CDCl_3_) *δ* 167.0, 161.2, 160.6, 158.5, 141.9, 141.6, 130.5, 130.3, 130.0, 128.9, 128.7, 125.9, 123.0, 120.8, 114.3, 55.3, 29.8, 15.0; HRMS (APCI): calcd. for C_22_H_22_N_3_O_2_S_2_ [M+H]^+^ 424.1148, found 424.1142.

### 3.5. Antifungal Activity Test

The in vitro antifungal activities of all target compounds against eight plant pathogenic fungi were evaluated at a concentration of 50 mg/L using the agar dilution method. The commercial fungicide boscalid was selected as the positive control. Test compounds were dissolved in acetone, with sterile distilled water serving as the blank control. Plates containing the drug medium were incubated at (24 ± 1) °C for 48 h, after which the mycelial diameter was measured. Values for each compound, expressed as the mean of three replicates, were compared with those of the blank control. The inhibition rate was finally calculated according to the following formula:

Inhibitory rate (%) = (***CK*** − ***PT***)/***CK*** × 100%

***PT***: Tested compound mycelium diameter; ***CK***: Blank control mycelium diameter

### 3.6. 3D-QSAR Analysis

Using Sybyl-X 2.1.1 software and nineteen target compounds, 3D-QSAR analysis based on the CoMFA method was performed, and the ED values were used to represent the inhibitory rate of the target compounds against *P. piricola*. The conversion formula is as follows:

ED = lg {***I***/[(100 − ***I***) × ***MW***]}

***I***: relative inhibitory rate; ***WM***: molecular weight

**Figure 7:**
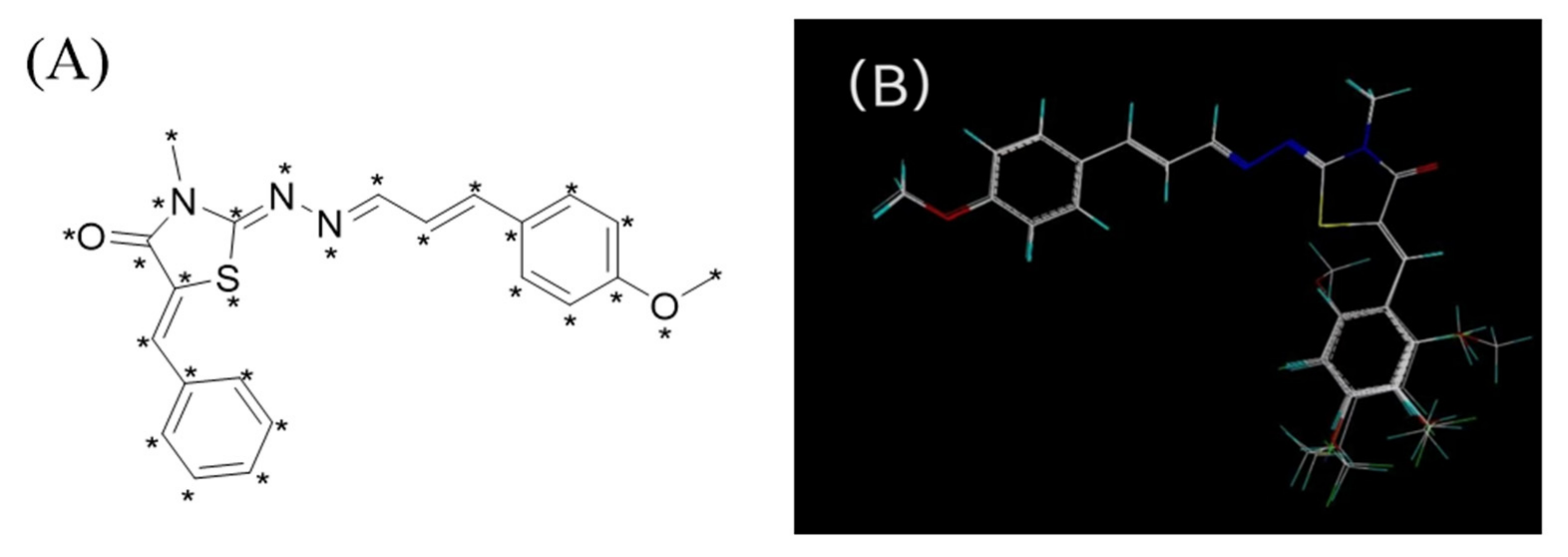
(**A**) The starred skeleton of the target compounds (the asterisk-marked atoms were selected as the common skeleton for superposition.); (**B**) The superimposed skeleton of the target compounds.

The optimized conformations of the target products **5a** to **5v** were generated using the Tripos force field and the Gasteiger–Hückel charge conjugate gradient method. The common skeleton atom asterisk labeling diagram of the nineteen compounds (Figure 7A) and the superimposition results are shown in Figure 7B. Finally, the constructed 3D-QSAR model was analyzed using partial least squares (PLS) to obtain the standard deviation, Fisher validation value, correlation coefficient, and cross-validation coefficient. Subsequently, the three compounds from the test set were used to further confirm the reliability of the established model.

### 3.7. Molecular Docking and Theoretical Calculation Analysis

Homology modeling was performed according to previously published methods and completed on the SWISS-MODEL web server (https://swissmodel.expasy.org/) (accessed on 17 December 2025) [4,38]. The 3D structure of SDH (PDB ID: 2WQY) was downloaded from the RSCB Protein Database (http://www.rcsb.org/) (accessed on 18 December 2025) and used as a template, while the target protein sequences corresponding to branches B, C, and D were obtained from the NCBI website (https://www.ncbi.nlm.nih.gov/) (accessed on 17 December 2025) as CEL53558.1, CCO28606.1, and CEL58000.1, respectively. Based on the established SDH structure, molecular docking was performed using AutoDock Tools-1.5.6 software, and an active pocket was predicted and screened for docking through AutoLigand. Molecular docking studies were conducted between the target compound **5q** (R = *m*-OH C_6_H_4_) with broad-spectrum antifungal activity and the positive control boscalid with SDH. The 3D and 2D interactions of the molecular docking were viewed on PyMOL 2.6.2 and LigPlus v2.2.5 software.

In addition, theoretical calculations of the electrostatic potential and frontier molecular orbitals of target compounds **5c** (R = *m*-CH_3_ C_6_H_4_) and **5q** (R = *m*-OH C_6_H_4_) were further carried out using Gaussian 09 [DFT/B3LYP/6-31G (d, p); it is a regular and commonly used functional and basis set, and this combination can reasonably describe the electronic distribution of the system while maintaining good computational accuracy] [39,40] on the high-performance computing platform of Guangxi University. The results were visualized using GaussView 5 software.

### 3.8. Characterization

The ^1^H NMR and ^13^C NMR spectra of compounds **3**, **4**, and **5a**–**5v** were measured using Bruker Avance III HD 600 MHz NMR spectrometer (Bruker, Zurich, Switzerland), with DMSO-d6 or CDCl3 as the solvent and TMS as the internal standard. The infrared spectra of target compounds **5a**–**5v** were measured on Nicolet iS 50 FT-IR spectrometer (Thermo Scientific Co., Ltd., Madison, WI, USA) using the KBr pellet method. High-resolution mass spectra of target compounds **5a**–**5v** were obtained on Q Exactive mass spectrometer (Thermo Scientific Co., Ltd., Madison, WI, USA), equipped with an APCI ion source. The melting points of target compounds **5a**–**5v** were determined on an MP420 automatic melting point apparatus (Hanon Co., Ltd., Jinan, China). All of these test results were correct.

## 4. Conclusions

In summary, starting from anethole-derived (*E*)-4-methoxycinnamaldehyde, twenty-two unreported thiazolinone-hydrazone compounds **5a**–**5v** were synthesized by multi-step reactions. These compounds contain a natural anethole skeleton, and their structures were fully characterized by ^1^H/^13^C NMR, HRMS, and FTIR. Preliminary bioactivity tests showed that **5q** (R = *m*-OH C_6_H_4_) exhibited the best inhibitory activity against most of the tested plant pathogenic fungi, with inhibition rates ranging from 51.3% to 85.0%, indicating that this compound had certain broad-spectrum antifungal activity. Based on the bioassay results, a statistically reliable 3D-QSAR model (*r*^2^ = 0.994, *q*^2^ = 0.529) was established using the CoMFA method to study the relationship between the structures of the target compounds and their antifungal activity against *P. piricola*. DFT calculations implied that the anethole framework and thiazolinone-hydrazone-phenyl group in the target compounds play an important role in their antifungal activity. At the same time, substituents subtly alter the local distribution of FMO and ESP in the target compounds, resulting in changes in antifungal activity. Moreover, molecular docking results showed that the target compound **5q** (R = *m*-OH C_6_H_4_) may have an action mechanism similar to that of inhibitors boscalid and **ref3a** targeting SDH. Overall, **5q** (R = *m*-OH C_6_H_4_) represented a promising lead candidate worthy of further investigation.

## Figures and Tables

**Table 1:** Antifungal activity of the target compounds **5a**–**5v** at 50 µg/mL.

	Relative Inhibition Rate (%) Against the Fungi							
Compounds	*F. oxysporum* f. sp. *cucumerinum*	*C. arachidicola*	*P.* *piricola*	*A. solani*	*G. zeae*	*R. solani*	*B. myadis*	*C. orbicalare*
**5a** (R = C_6_H_5_)	29.0 ± 0.5	16.7 ± 1.1	17.1 ± 0.6	37.3 ± 0.9	37.8 ± 2.3	63.1 ± 0.7	20.5 ± 0.8	29.0 ± 1.6
**5b** (R = *o*-CH_3_ C_6_H_4_)	33.8 ± 0.7	23.3 ± 0.6	38.6 ± 2.4	32.7 ± 1.5	37.8 ± 0.8	13.1 ± 1.2	15.3 ± 1.5	24.3 ± 0.4
**5c** (R = *m*-CH_3_ C_6_H_4_)	23.8 ± 1.3	30.0 ± 0.9	27.9 ± 1.3	37.3 ± 1.2	37.8 ± 1.5	41.3 ± 0.6	25.8 ± 2.4	24.3 ± 1.3
**5d** (R = *p*-CH_3_ C_6_H_4_)	33.8 ± 2.2	16.7 ± 2.1	31.4 ± 0.3	28.2 ± 0.8	35.0 ± 1.8	28.8 ± 0.9	20.5 ± 2.2	24.3 ± 1.8
**5e** (R = *o*-OCH_3_ C_6_H_4_)	24.3 ± 1.6	30.0 ± 0.6	31.4 ± 0.8	37.3 ± 0.7	32.2 ± 1.1	22.5 ± 1.7	20.5 ± 0.6	24.3 ± 0.9
**5f** (R = *m*-OCH_3_ C_6_H_4_)	33.8 ± 0.2	16.7 ± 0.6	35.0 ± 0.9	32.7 ± 1.9	32.2 ± 1.6	31.9 ± 1.4	20.5 ± 2.1	19.5 ± 1.2
**5g** (R = *p*-OCH_3_ C_6_H_4_)	24.3 ± 0.6	23.3 ± 0.9	24.3 ± 1.5	37.3 ± 0.9	40.6 ± 2.3	28.8 ± 0.7	20.5 ± 0.8	24.3 ± 0.5
**5h** (R = *o*-F C_6_H_4_)	33.8 ± 2.3	23.3 ± 1.4	35.0 ± 2.3	37.3 ± 0.6	46.1 ± 0.7	44.4 ± 2.6	20.5 ± 1.4	24.3 ± 1.9
**5i** (R = *p*-F C_6_H_4_)	33.8 ± 1.7	23.3 ± 2.4	52.9 ± 0.8	41.8 ± 2.1	32.2 ± 0.9	18.8 ± 1.9	10.5 ± 1.6	19.0 ± 1.1
**5j** (R = *o*-Cl C_6_H_4_)	33.8 ± 1.2	23.3 ± 0.7	17.1 ± 0.3	19.1 ± 1.7	23.9 ± 1.3	28.8 ± 1.0	25.8 ± 0.9	14.8 ± 1.7
**5k** (R = *m*-Cl C_6_H_4_)	33.8 ± 0.8	23.3 ± 1.2	45.7 ± 2.1	41.8 ± 1.3	37.8 ± 1.5	35.0 ± 1.1	25.8 ± 1.8	29.0 ± 0.2
**5l** (R = *p*-Cl C_6_H_4_)	33.8 ± 0.5	16.7 ± 1.5	42.1 ± 1.3	41.8 ± 0.7	35.0 ± 0.8	19.4 ± 0.7	20.5 ± 0.4	29.0 ± 0.8
**5m** (R = *o*-Br C_6_H_4_)	24.3 ± 2.2	16.7 ± 1.8	17.1 ± 1.1	32.7 ± 1.2	26.7 ± 1.4	28.8 ± 1.1	15.3 ± 2.1	33.8 ± 0.4
**5n** (R = *m*-Br C_6_H_4_)	33.8 ± 0.8	16.7 ± 0.9	24.3 ± 2.6	37.3 ± 0.8	37.8 ± 1.0	28.8 ± 0.6	25.8 ± 1.6	24.3 ± 2.0
**5o** (R = *p*-I C_6_H_4_)	33.8 ± 1.7	16.7 ± 1.6	31.4 ± 0.9	37.3 ± 2.4	37.8 ± 0.6	31.9 ± 0.7	15.3 ± 0.9	24.3 ± 0.5
**5p** (R = *m*-CF_3_ C_6_H_4_)	33.8 ± 1.1	16.7 ± 1.7	24.3 ± 0.6	41.8 ± 2.1	37.8 ± 1.6	41.3 ± 1.2	15.3 ± 1.7	24.3 ± 2.1
**5q** (R = *m*-OH C_6_H_4_)	51.3 ± 1.8	71.3 ± 2.1	85.0 ± 1.4	72.5 ± 1.6	53.9 ± 0.9	53.1 ± 2.5	70.6 ± 1.1	64.6 ± 0.8
**5r** (R = *o*-OH C_6_H_4_)	29.0 ± 0.6	23.3 ± 0.9	52.9 ± 1.6	46.4 ± 2.1	40.6 ± 1.2	56.9 ± 1.1	15.3 ± 1.8	24.3 ± 0.6
**5s** (R = *p*-OCF_3_ C_6_H_4_)	33.8 ± 0.7	30.0 ± 0.7	35.0 ± 0.3	37.3 ± 1.5	40.6 ± 2.7	50.6 ± 1.5	20.5 ± 2.6	29.0 ± 1.8
**5t** (R = *p*-Br-*m*-F C_6_H_3_)	24.3 ± 0.4	16.7 ± 0.0	17.1 ± 0.7	37.3 ± 2.6	37.8 ± 1.9	31.9 ± 0.8	20.5 ± 1.9	24.3 ± 0.8
**5u** (R = *o*, *p*-OCH_3_ C_6_H_3_)	33.8 ± 2.5	16.7 ± 0.3	42.1 ± 2.2	32.7 ± 1.1	32.2 ± 0.9	41.3 ± 1.2	15.3 ± 1.7	19.5 ± 1.6
**5v** (R = *p*-SCH_3_ C_6_H_4_)	14.8 ± 0.9	16.7 ± 1.1	49.3 ± 1.8	37.3 ± 1.4	37.8 ± 0.9	28.8 ± 0.6	15.3 ± 0.7	24.3 ± 1.3
Boscalid ^a^	26.9 ± 1.1	82.4 ± 0.8	60.2 ± 1.4	65.9 ± 1.7	12.0 ± 0.5	46.5 ± 1.4	30.8 ± 1.2	25.0 ± 2.1

a: Positive control commercial sterilant. The experiment was repeated three times.

**Table 2:** The ED values of experimental and predicted activities.

Compd.	MW	Exp. ED	Pre. ED	Residue
**5a** (R = C_6_H_5_)	377.11	−3.26	−3.28	0.02
**5b** (R = *o*-CH_3_ C_6_H_4_)	391.13	−2.79	−2.81	0.02
**5c** (R = *m*-CH_3_ C_6_H_4_)	391.13	−3.00	−2.98	−0.02
**5d** (R = *p*-CH_3_ C_6_H_4_) ^a^	391.13	−2.93	−2.94	0.01
**5e** (R = *o*-OCH_3_ C_6_H_4_)	407.13	−2.94	−2.95	0.01
**5f** (R = *m*-OCH_3_ C_6_H_4_)	407.13	−2.87	−2.87	0
**5g** (R = *p*-OCH_3_ C_6_H_4_)	407.13	−3.10	−3.10	0
**5h** (R = *o*-F C_6_H_4_)	395.11	−2.86	−2.85	−0.01
**5i** (R = *p*-F C_6_H_4_)	395.11	−2.54	−2.55	0.01
**5j** (R = *o*-Cl C_6_H_4_)	411.08	−3.29	−3.29	0
**5k** (R = *m*-Cl C_6_H_4_)	411.08	−2.68	−2.77	0.01
**5l** (R = *p*-Cl C_6_H_4_)	411.08	−2.75	−2.74	−0.01
**5m** (R = *o*-Br C_6_H_4_)	455.03	−3.34	−3.35	0.01
**5n** (R = *m*-Br C_6_H_4_)	455.03	−3.15	−3.09	−0.06
**5o** (R = *p*-I C_6_H_4_)^a^	503.01	−3.04	−3.01	−0.03
**5p** (R = *m*-CF_3_ C_6_H_4_)	445.10	−3.14	−3.13	−0.01
**5q** (R = *m*-OH C_6_H_4_)	393.11	−1.84	−1.83	−0.01
**5r** (R = *o*-OH C_6_H_4_)	393.11	−2.54	−2.50	−0.04
**5s** (R = *p*-OCF_3_ C_6_H_4_)	461.10	−2.93	−2.94	0.01
**5t** (R = *p*-Br-*m*-F C_6_H_3_) ^a^	473.02	−3.36	−3.36	0
**5u** (R = *o*, *p*-OCH_3_ C_6_H_3_)	437.14	−2.77	−2.77	0
**5v** (R = *p*-SCH_3_ C_6_H_4_)	423.10	−2.63	−2.65	0.01

Exp. ED: experimental value; Pre. ED: predictive value; ^a^: test-set compound.

**Table 3:** Summary of CoMFA analysis.

Statistical Parameters	CoMFA
*q* ^2^	0.529
*r* ^2^	0.994
*S*	0.033
*F*	116.271
**Field contribution (%)**	
Steric	52.3
Electrostatic	47.7

*q*^2^: cross-validated correlational coefficient; *r*^2^: non-validated correlational coefficient; *S*: standard error of estimate; *F*: the Fischer ratio.

## Data Availability

Data is contained within the article or the Supplementary Materials.

## Supplementary Materials

The following supporting information can be downloaded at: https://www.mdpi.com/article/10.3390/molecules31071078/s1, Figures S1–S92: FT-IR, ^1^H-NMR, ^13^C-NMR, and HRMS spectra of compounds. Figure S93: 3D structure (a) and Ramachandran plot (b) [PDB ID: 2WQY (Remodelling of carboxin binding to the Q-site of avian respiratory complex II), homology modeling on SWISS-MODEL web]. Figure S94: The model of sequence alignments based on 2WQY. Figure S95: The model quality estimate of homology modeling.

## Sample Availability

Samples of the compounds are available from the authors.
