# Supplementary material for: Synthesis, Antifungal Activity, 3D-QSAR, and Molecular Docking Study of Anethole-Based Thiazolinone-Hydrazone Compounds
Source: Molecules. 2026 Mar 25;31(7):1078. doi: 10.3390/molecules31071078 (PMC13074763; doi:10.3390/molecules31071078)
Supplement: Supplementary file 1 [file molecules-31-01078-s001.zip › molecules-4214863-supplementary.pdf]

Supporting Material

Synthesis, Antifungal Activity, 3D-QSAR, and Molecular Docking Study of Anethole-Based Thiazolinone-Hydrazone Compounds

Yao Chen[1], Yucheng Cui[1], Youqiong Bi[1], Zhangli Guo[1], Xianli Ma[1,2], Wengui Duan[1,*] Guishan Lin[1,*]

[1] School of Chemistry and Chemical Engineering, Guangxi University, Nanning 530004, Guangxi, China; cyygxu2026@163.com (Y.C.); cuiyc1109@126.com (Y.C.); biyouqiong1021@163.com (Y.B.); guo08292026@163.com (Z.G.); mxl78@glmc.edu.cn (X.M.)

[2] Guilin Medical University, Guangxi Key Laboratory for Pharmaceutical Molecular Discovery and Druggability Optimization, Guilin 541199, Guangxi, China; mxl78@glmc.edu.cn (X.M.)

* Corresponding Authors: wgduan@gxu.edu.cn (Wengui Duan),

gslin@gxu.edu.cn (Guishan Lin).

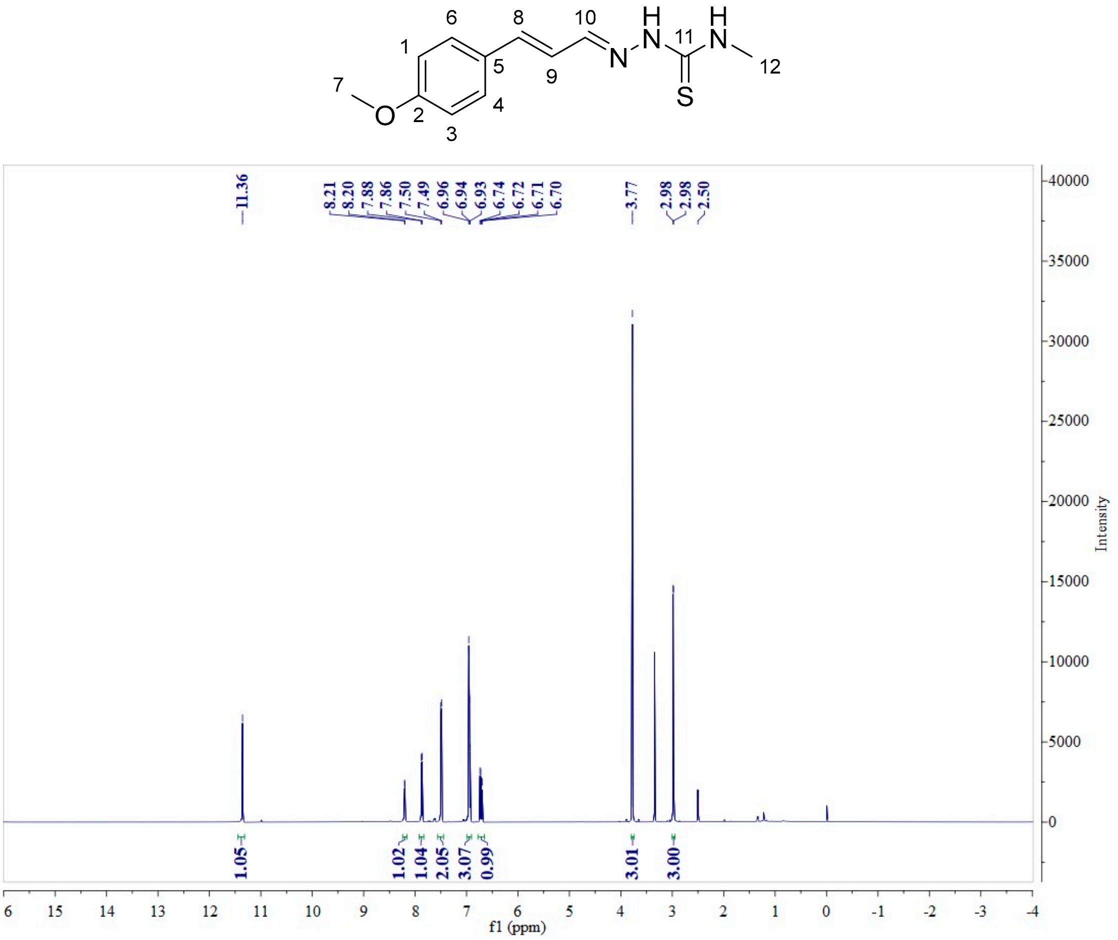

Figure S1. ^{1}H-NMR spectrum of intermediate **3**.

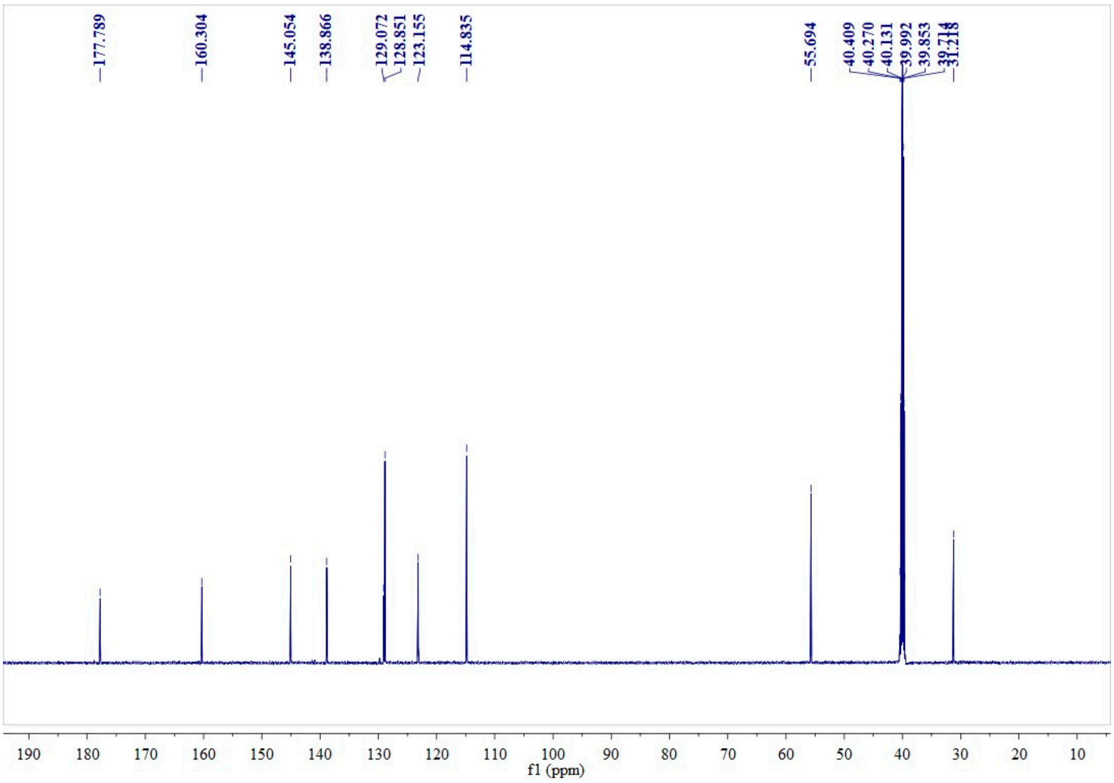

Figure S2. ^{13}C-NMR spectrum of intermediate **3**.

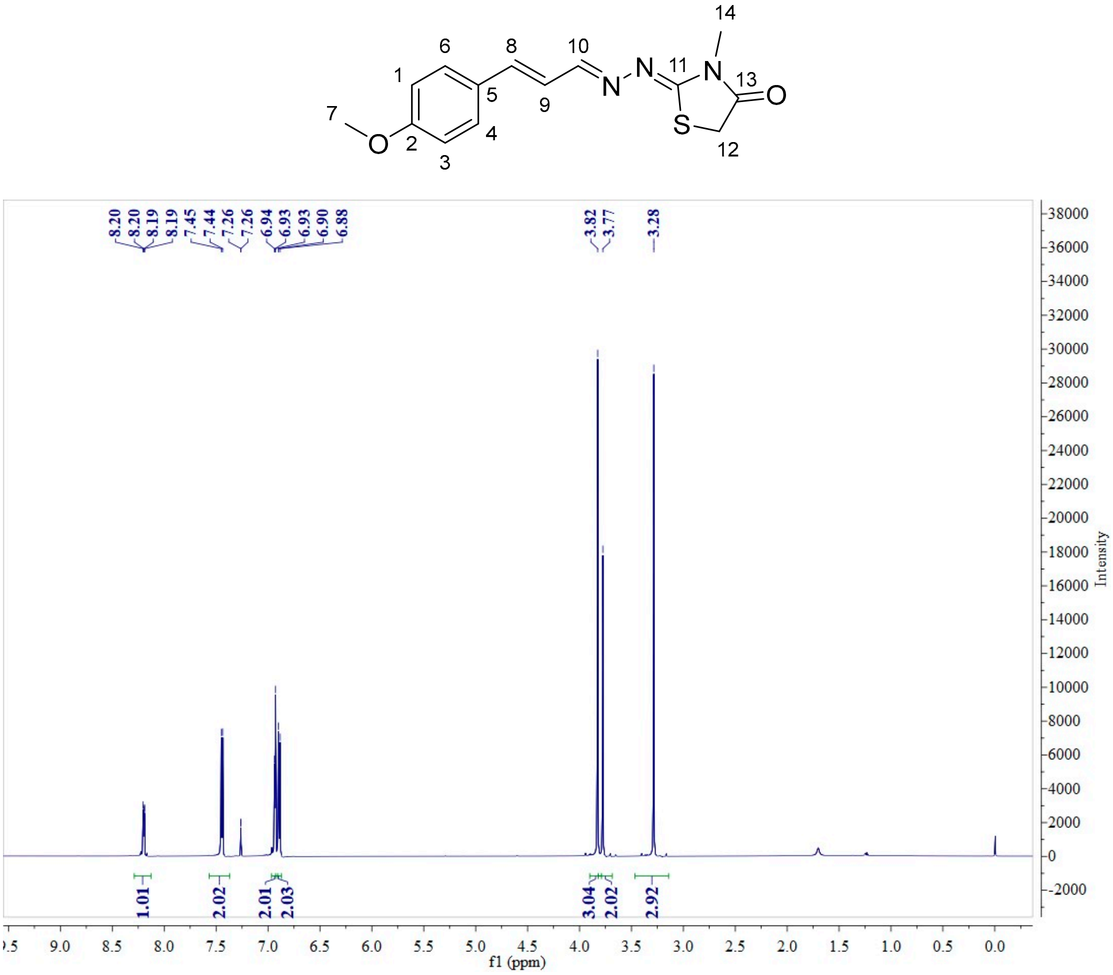

Figure S3. ^{1}H-NMR spectrum of intermediate 4.

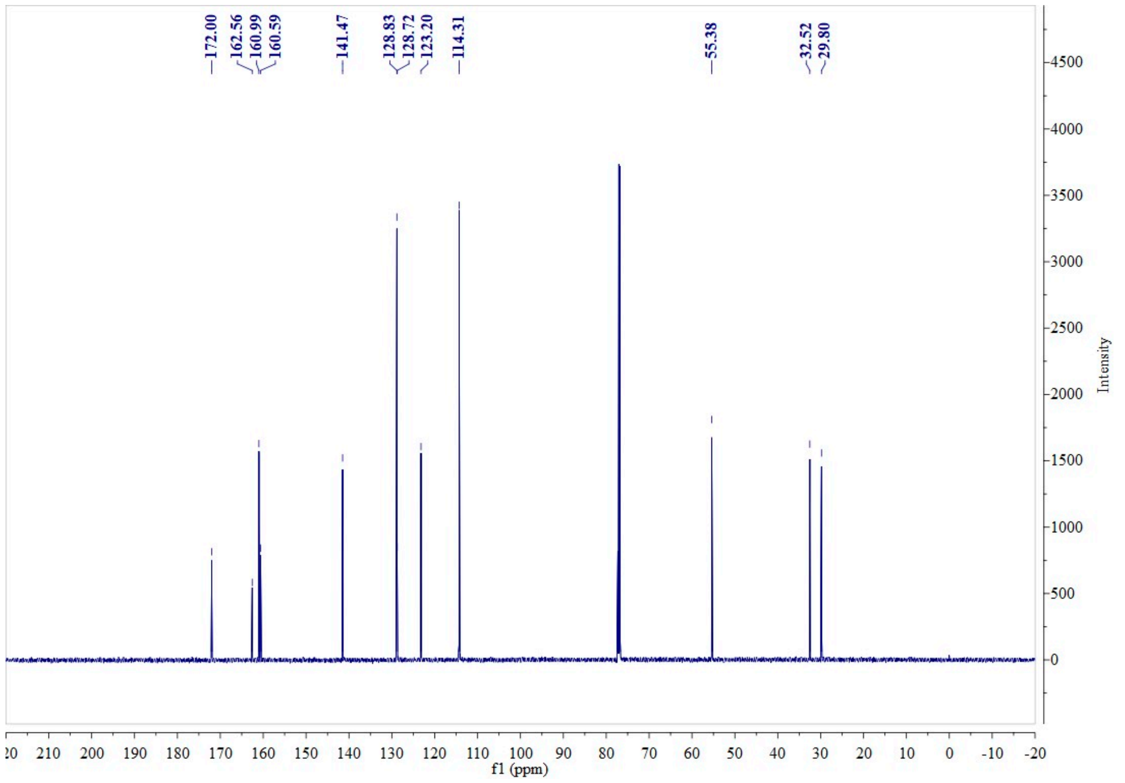

Figure S4. ^{13}C-NMR spectrum of intermediate 4.

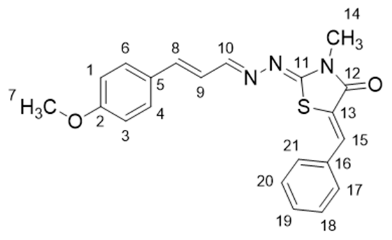

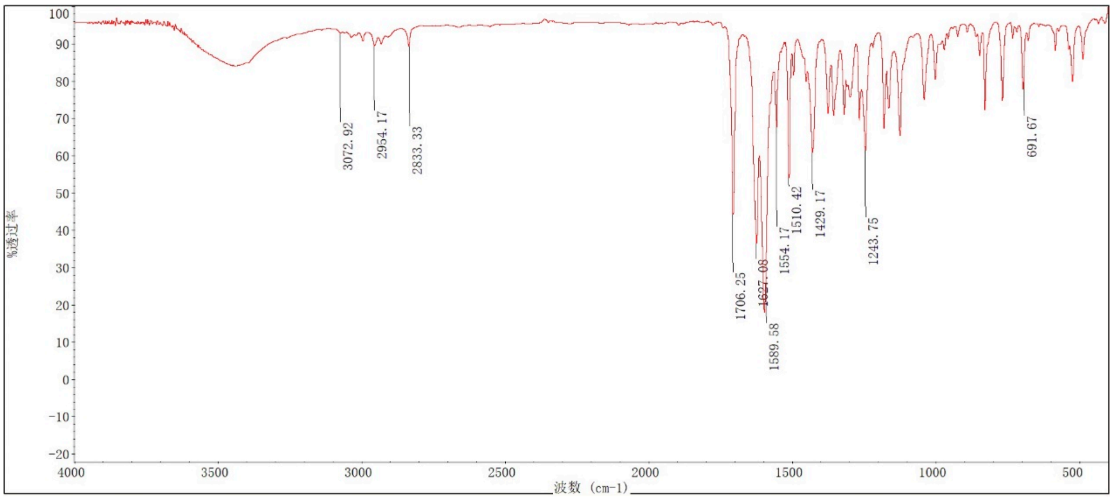

Figure S5. FT-IR spectrum of compound **5a**.

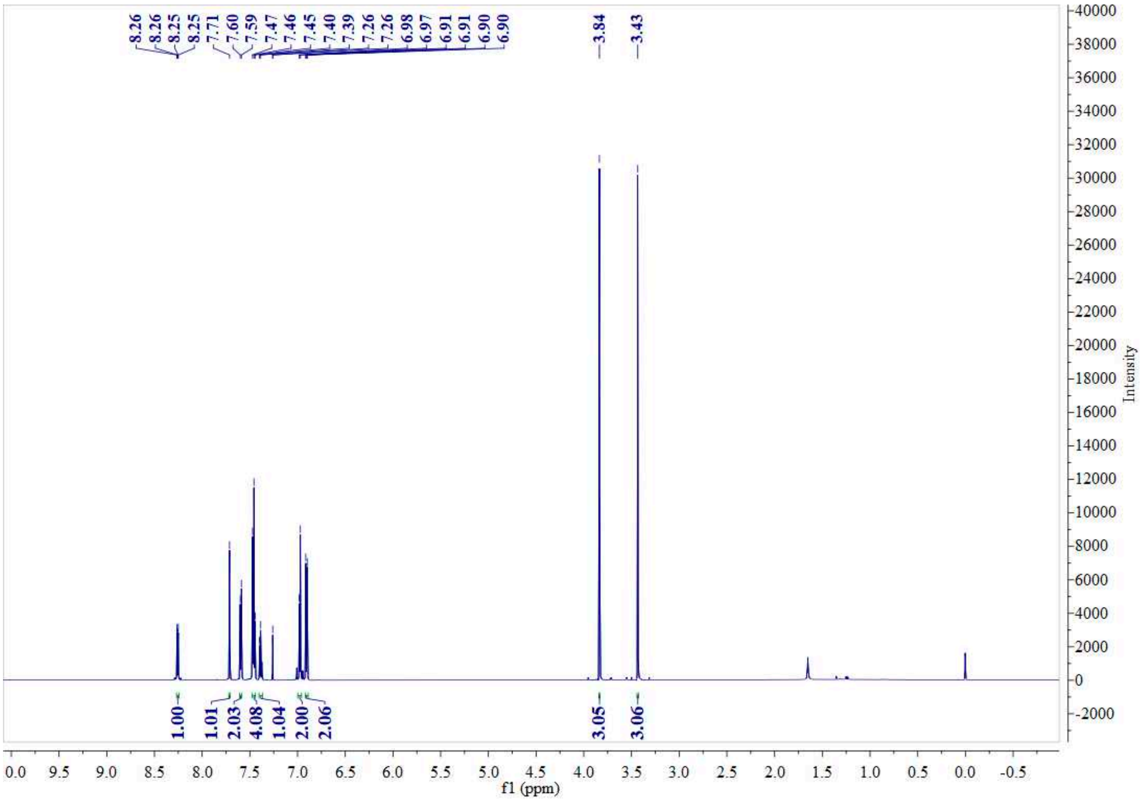

Figure S6. ^{1}H-NMR spectrum of compound **5a**.

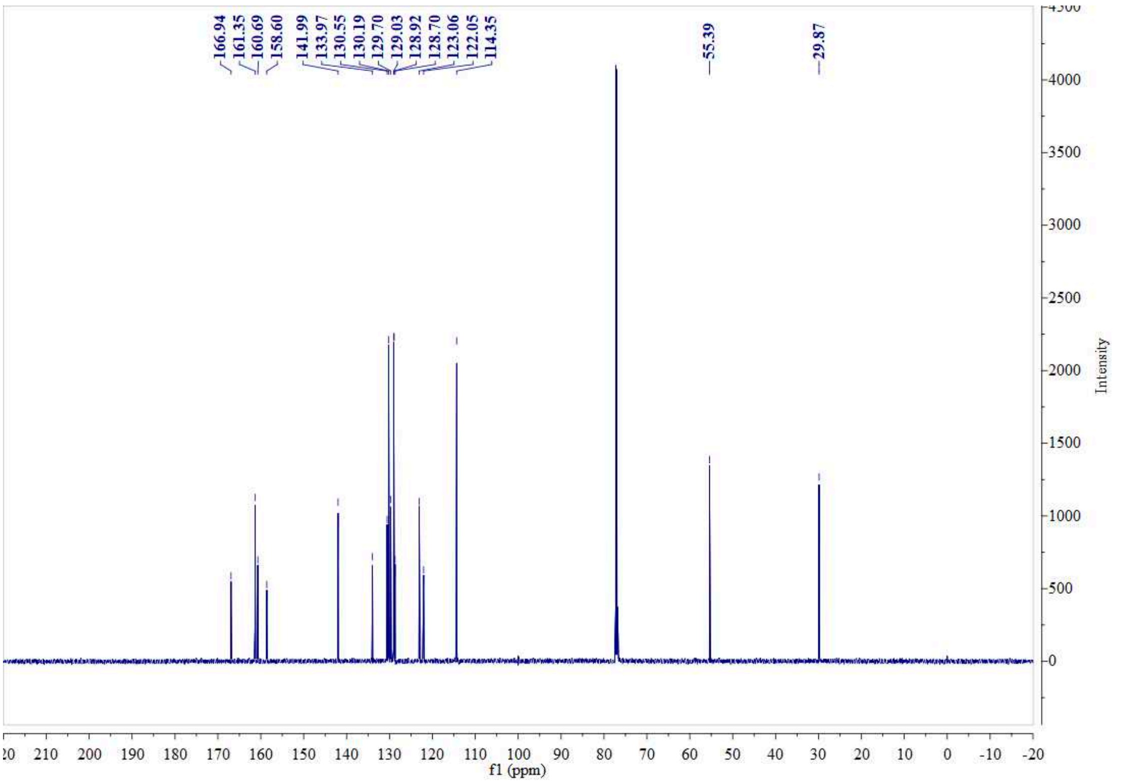

Figure S7. ^{13}C-NMR spectrum of compound **5a**.

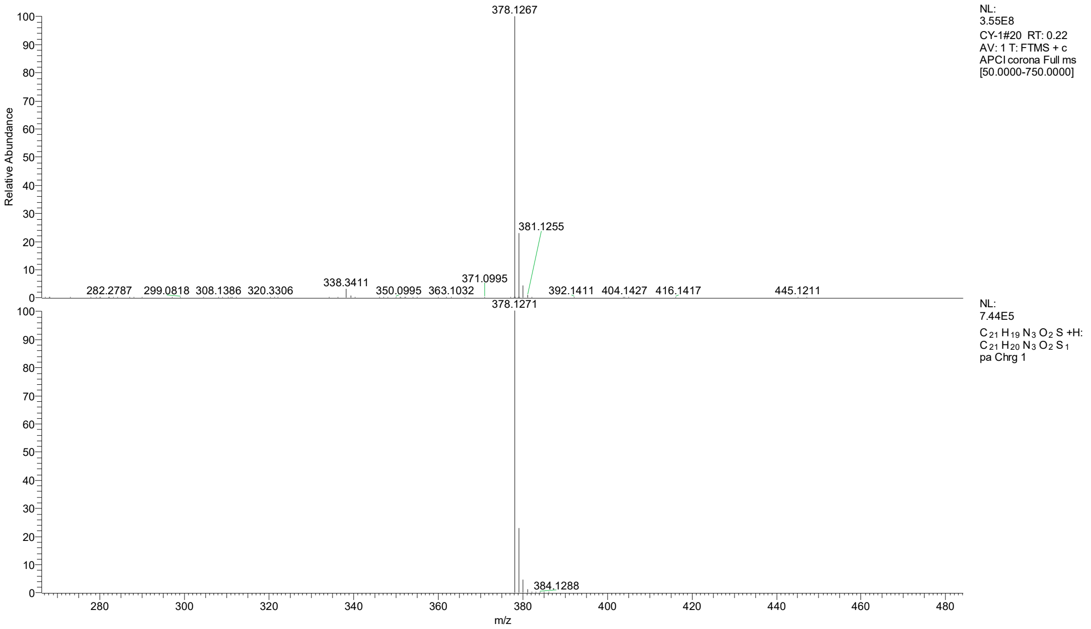

Figure S8. HRMS spectrum of compound **5a**.

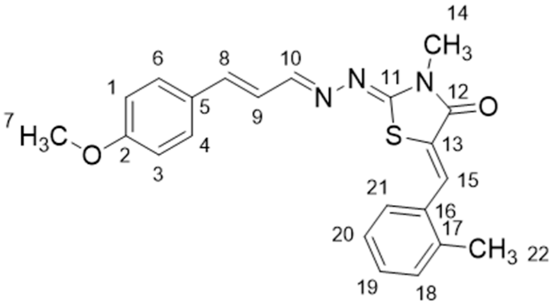

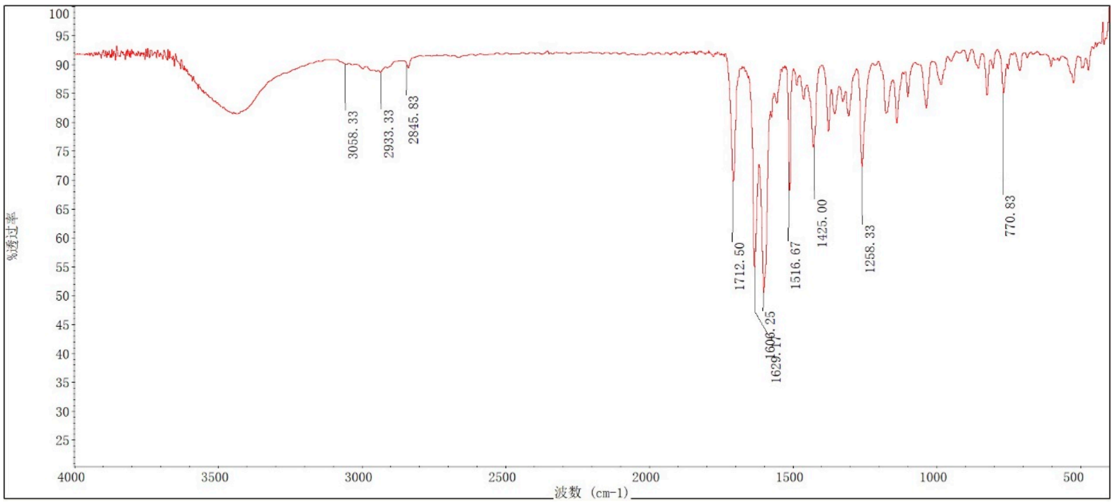

Figure S9. FT-IR spectrum of compound **5b**.

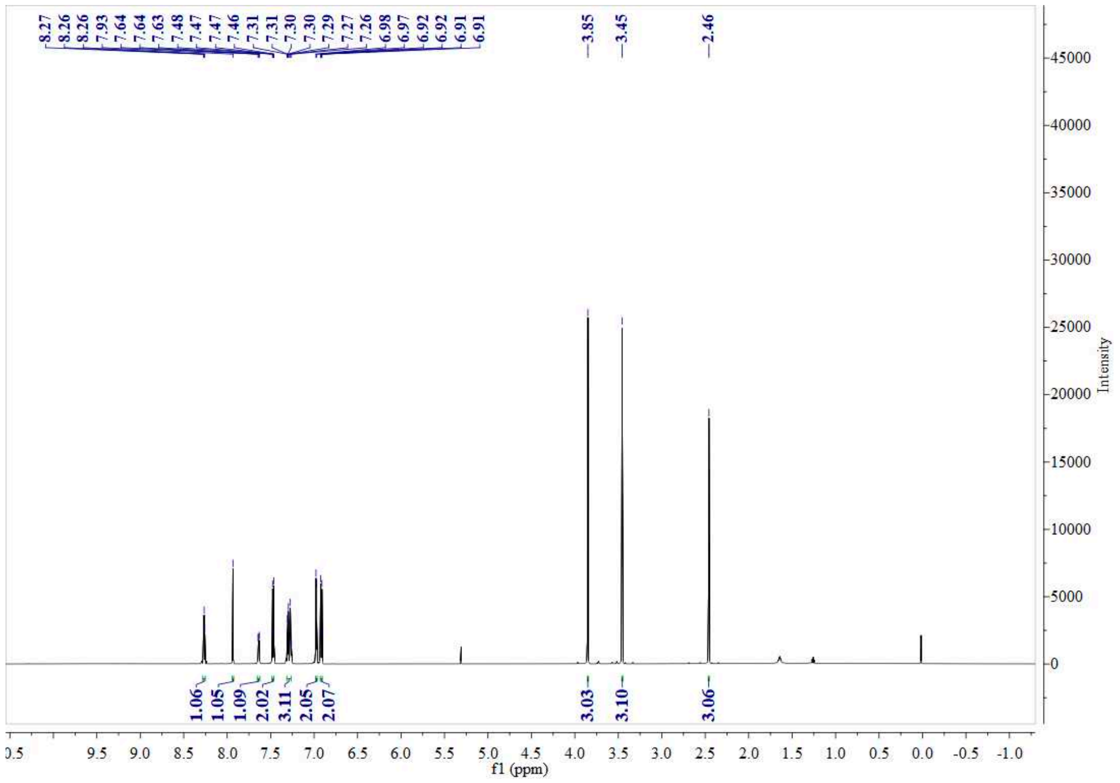

Figure S10. ¹H-NMR spectrum of compound **5b**.

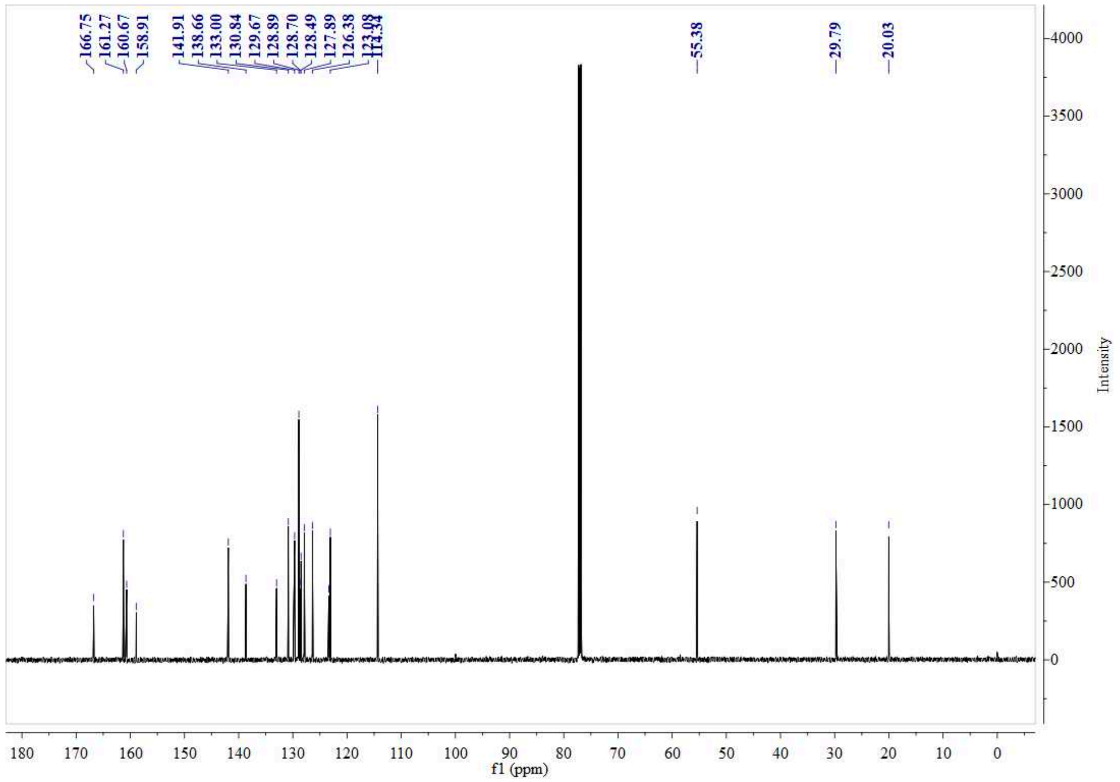

Figure S11. ¹³C-NMR spectrum of compound **5b**.

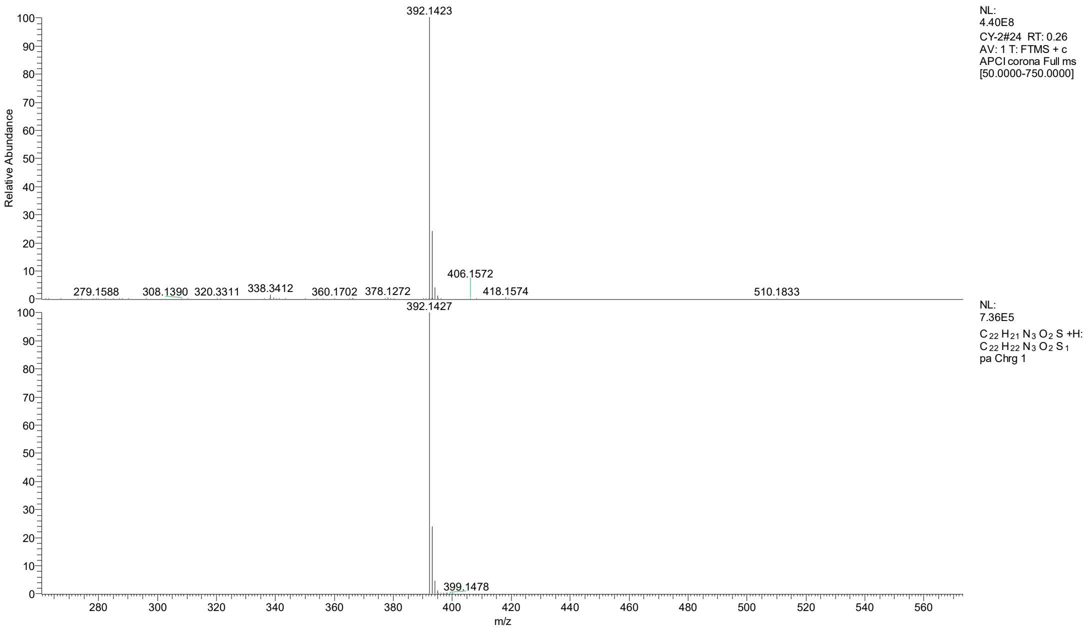

Figure S12. HRMS spectrum of compound **5b**.

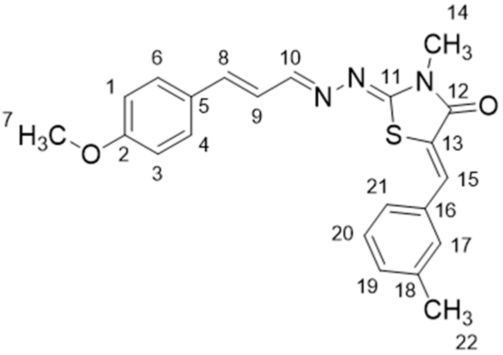

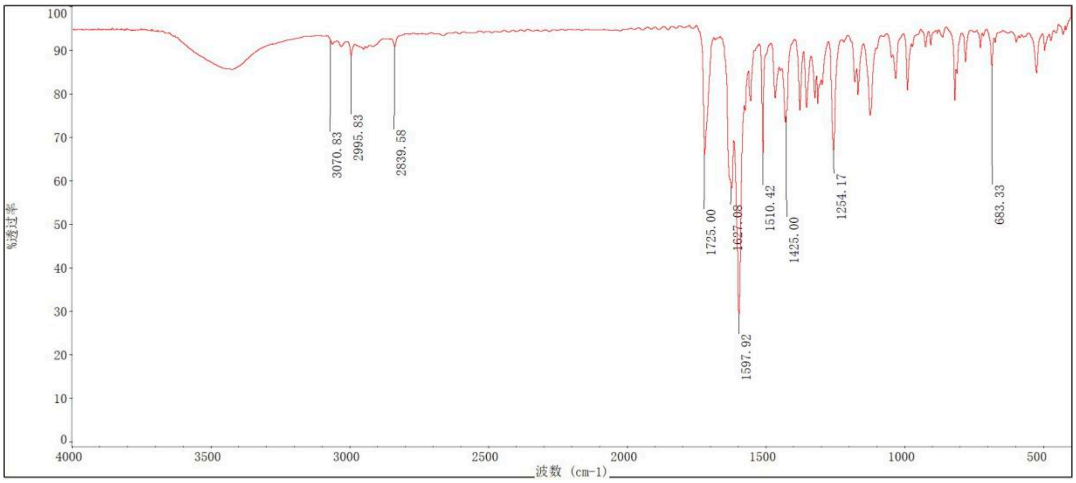

Figure S13. FT-IR spectrum of compound **5c**.

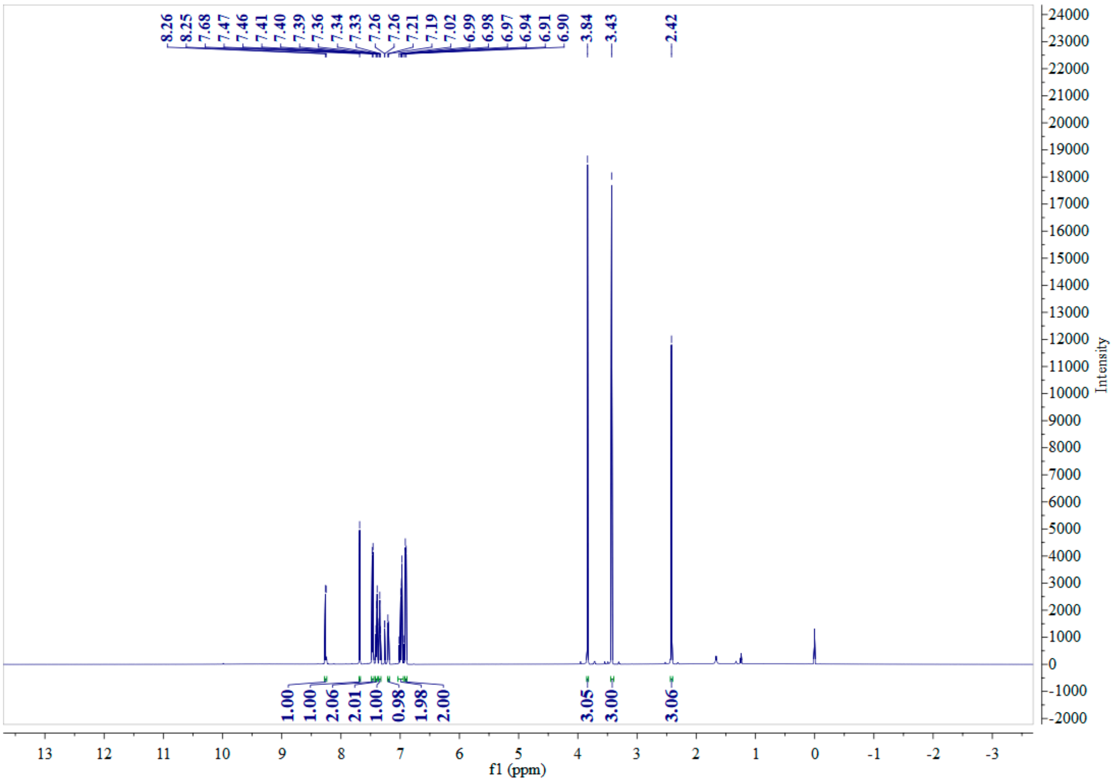

Figure S14. ^{1}H-NMR spectrum of compound **5c**.

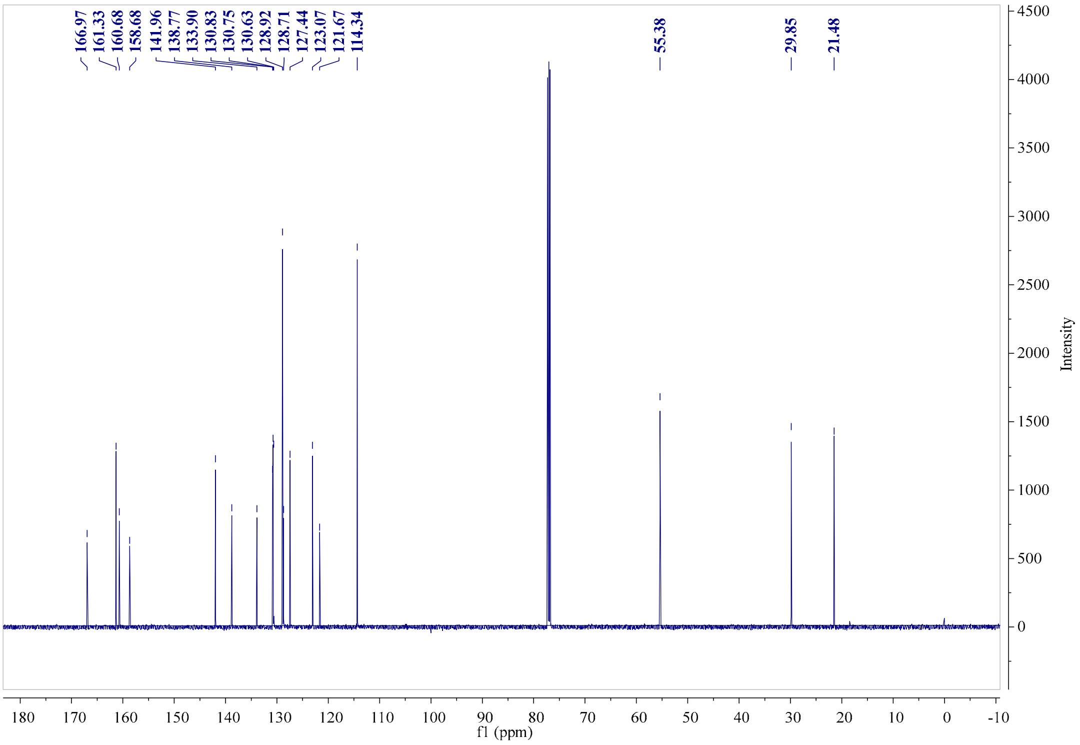

Figure S15. ^{13}C-NMR spectrum of compound **5c**.

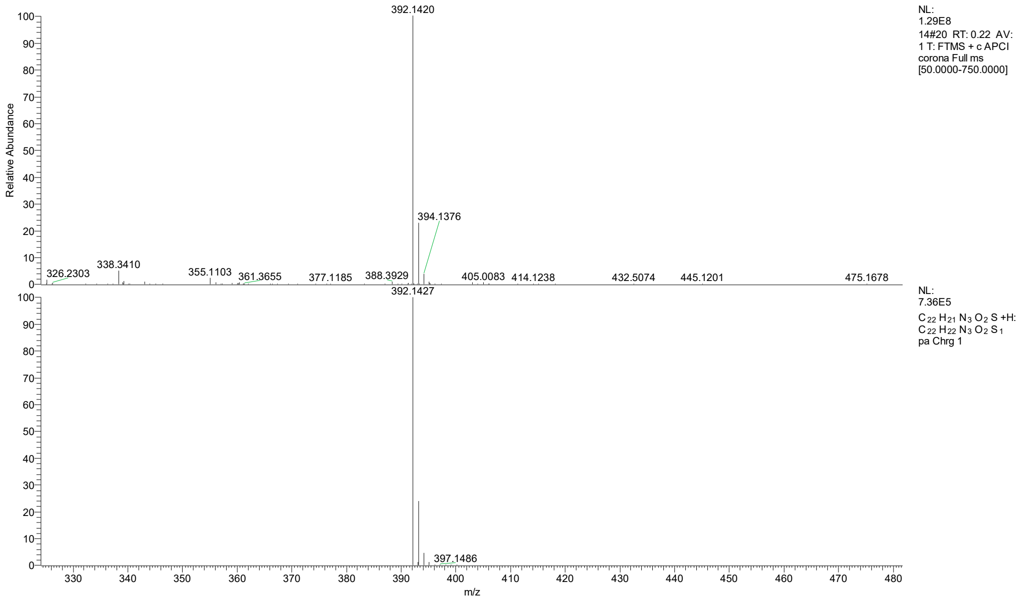

Figure S16. HRMS spectrum of compound **5c**.

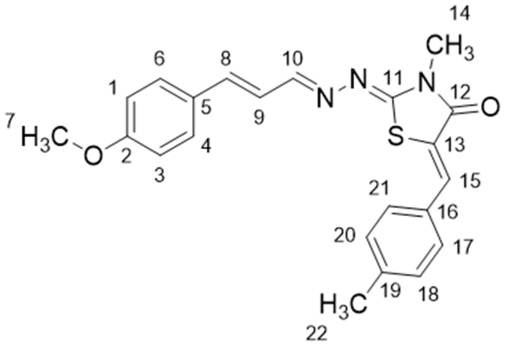

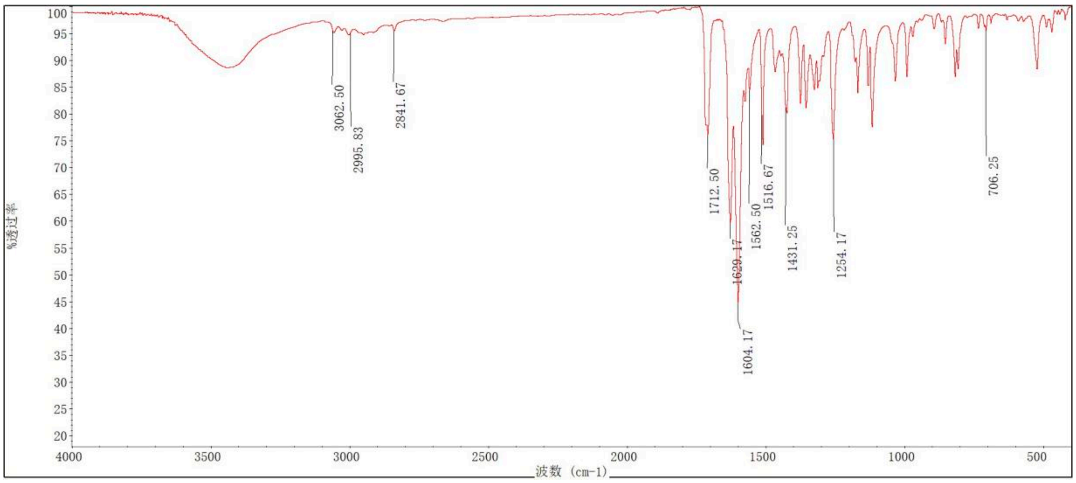

Figure S17. FT-IR spectrum of compound **5d**.

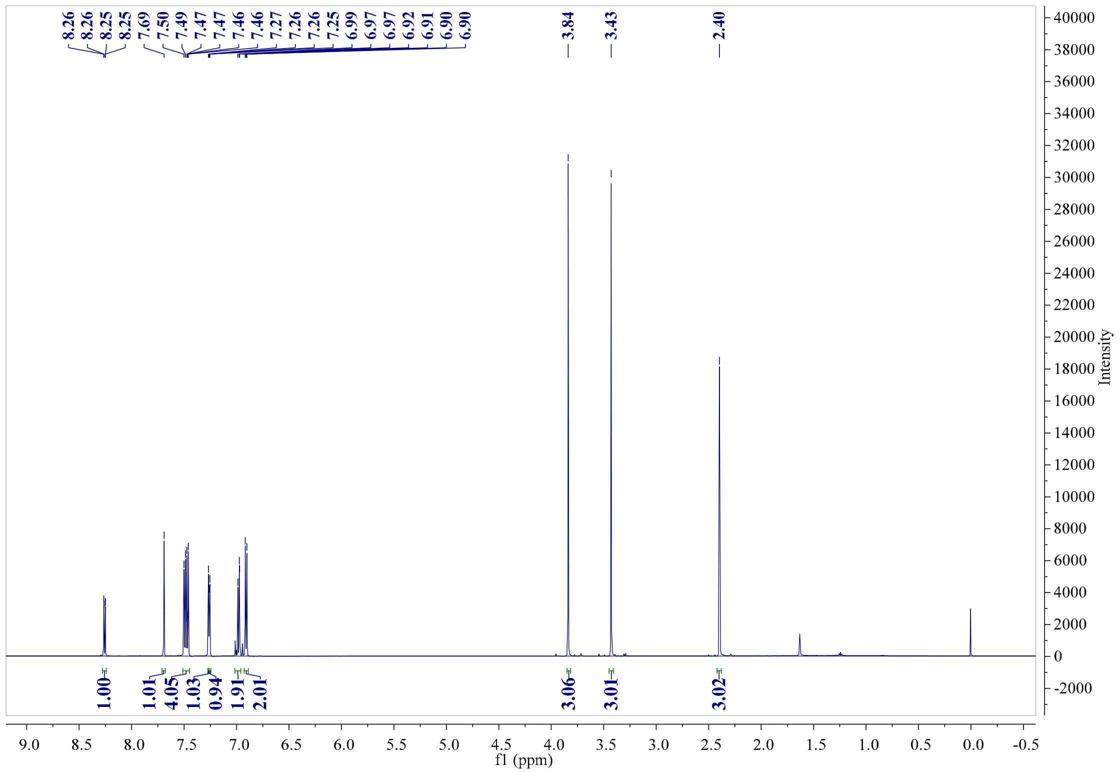

Figure S18. ^{1}H-NMR spectrum of compound **5d**.

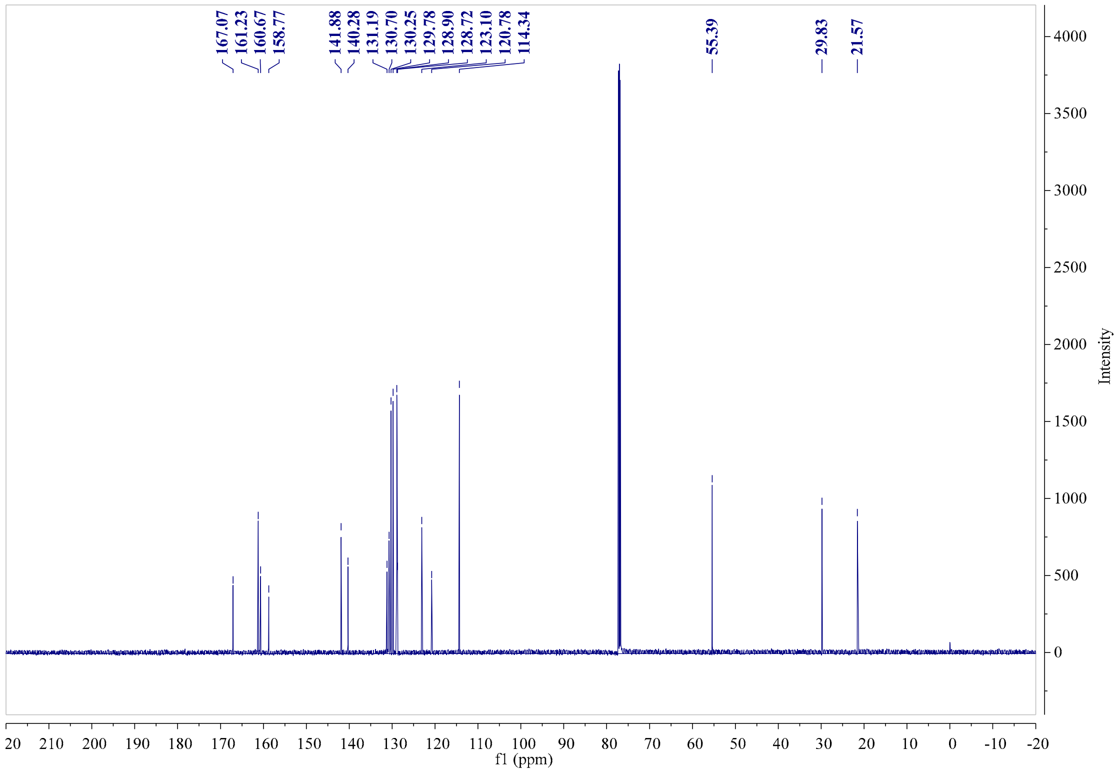

Figure S19. ^{13}C-NMR spectrum of compound **5d**.

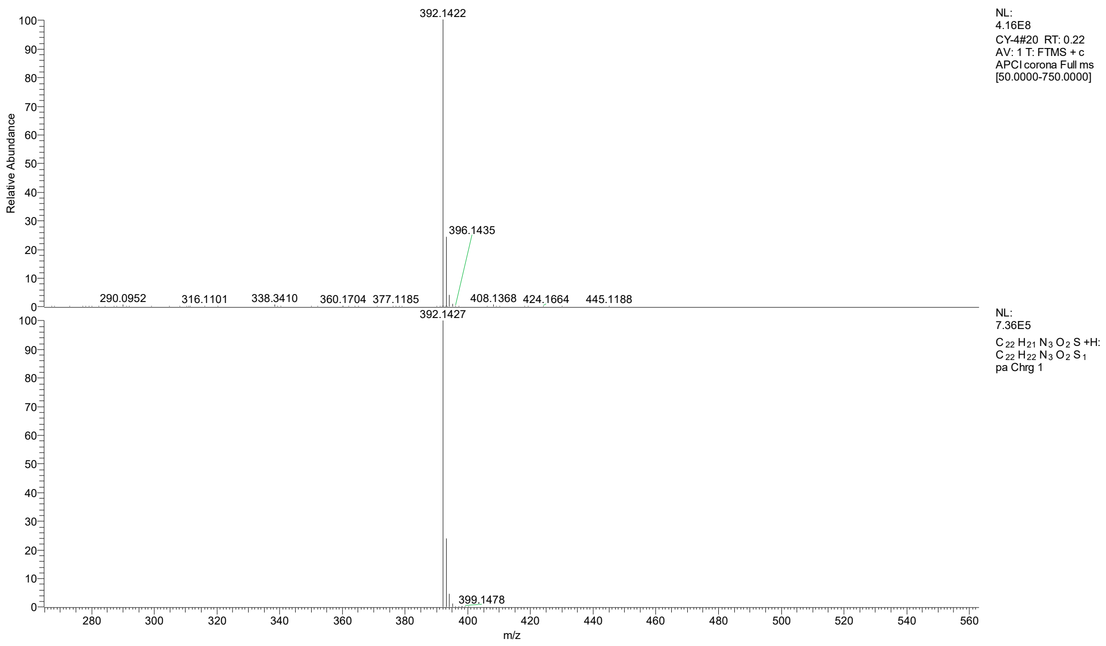

Figure S20. HRMS spectrum of compound **5d**.

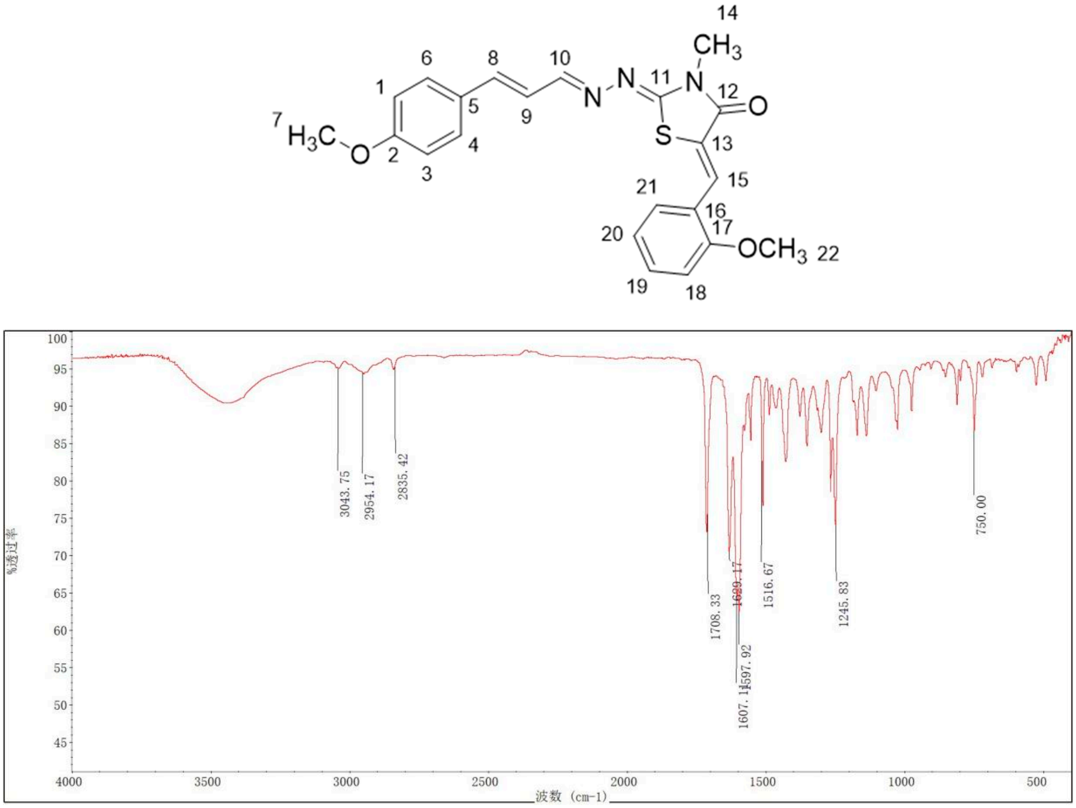

Figure S21. FT-IR spectrum of compound **5e**.

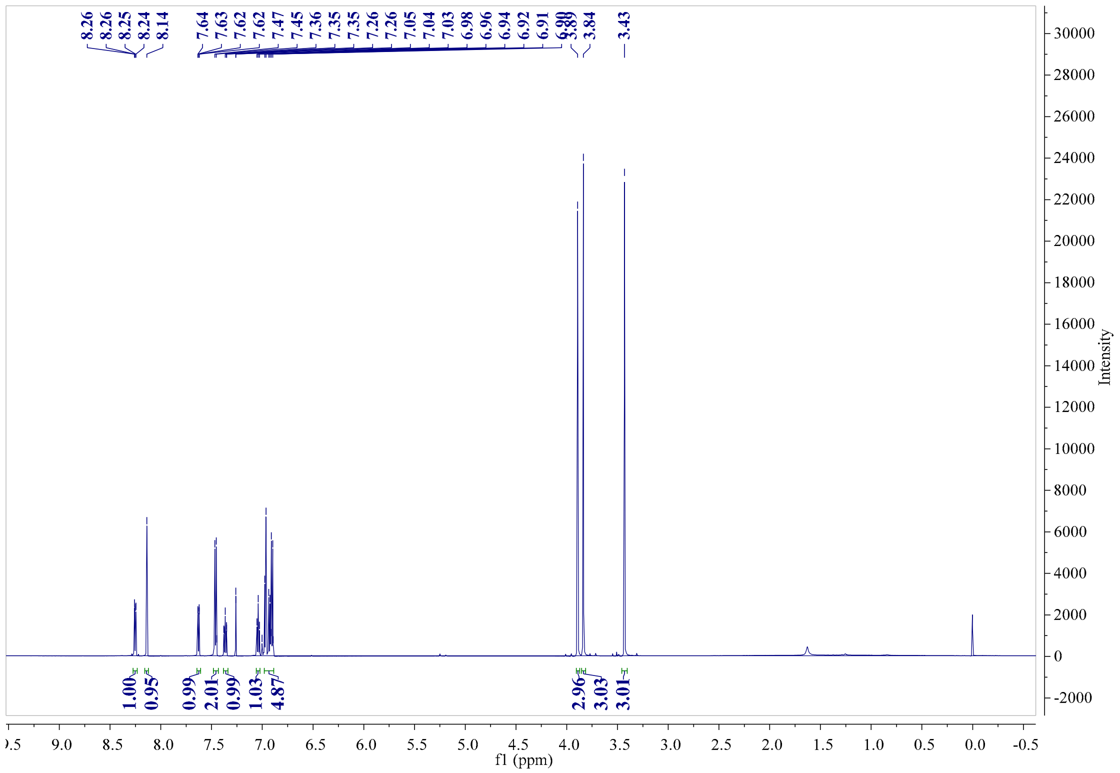

Figure S22. ^{1}H-NMR spectrum of compound **5e**.

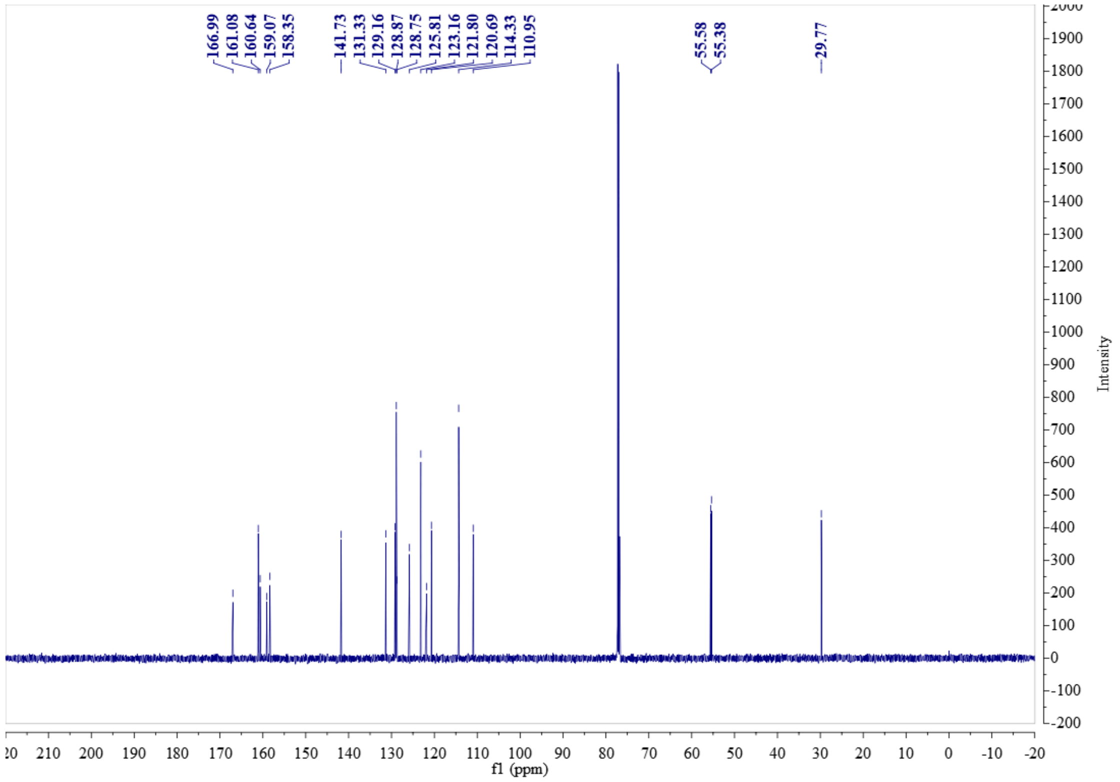

Figure S23. ^{13}C-NMR spectrum of compound **5e**.

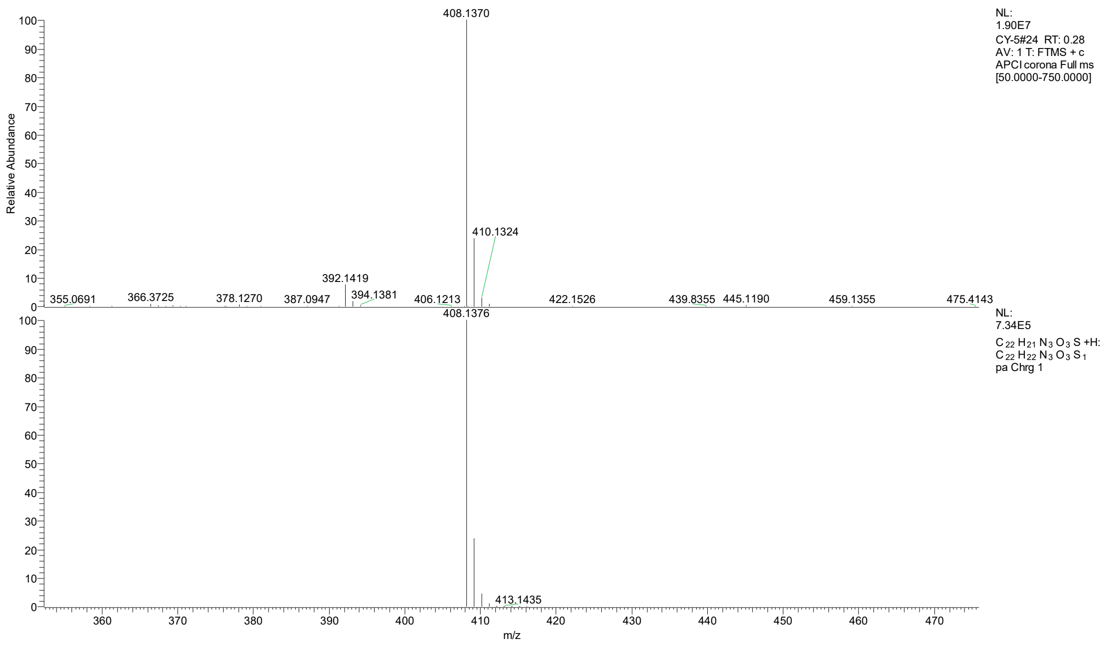

Figure S24. HRMS spectrum of compound **5e**.

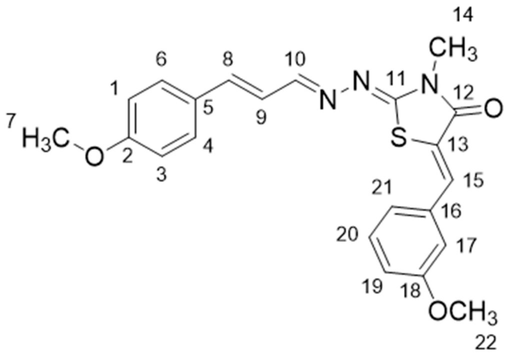

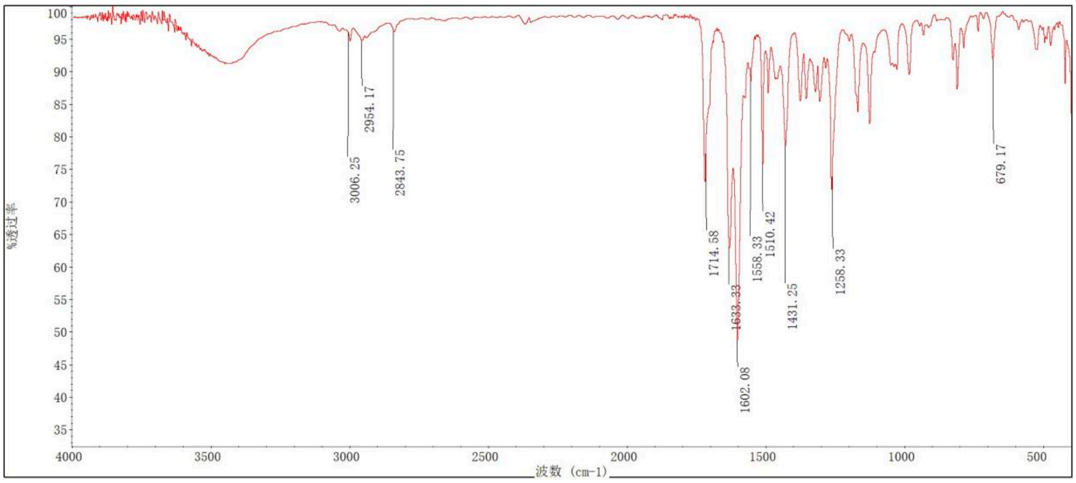

Figure S25. FT-IR spectrum of compound **5f**.

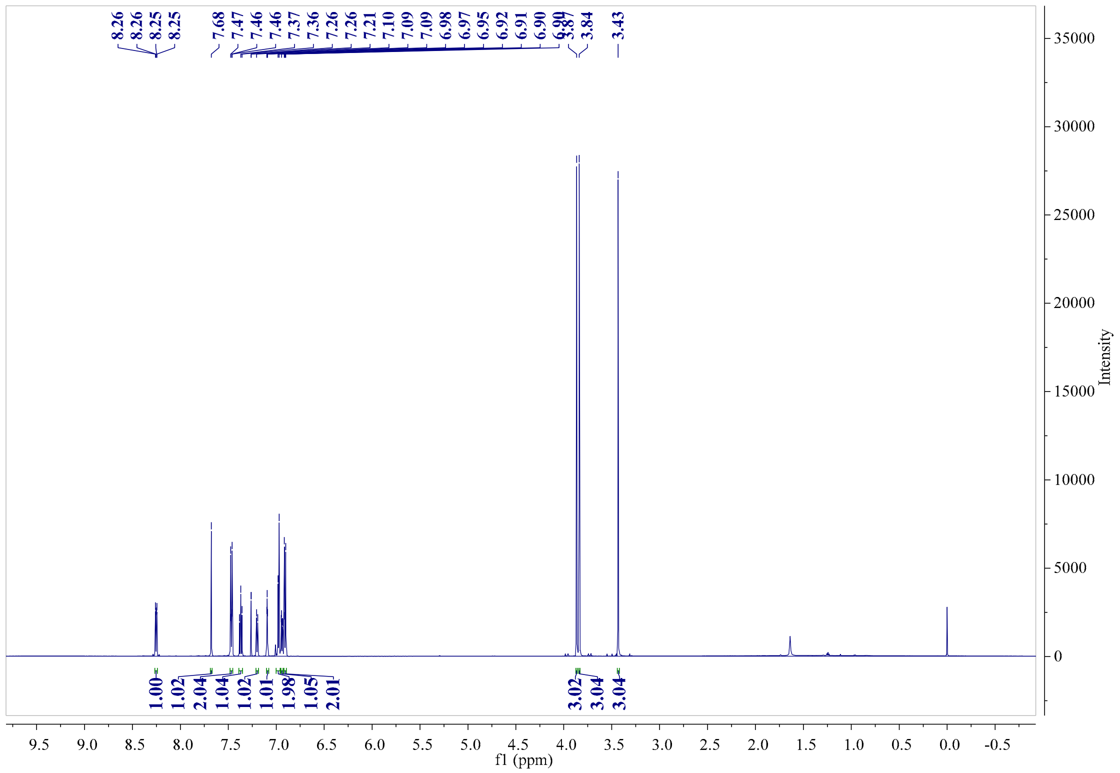

Figure S26. ^{1}H-NMR spectrum of compound **5f**.

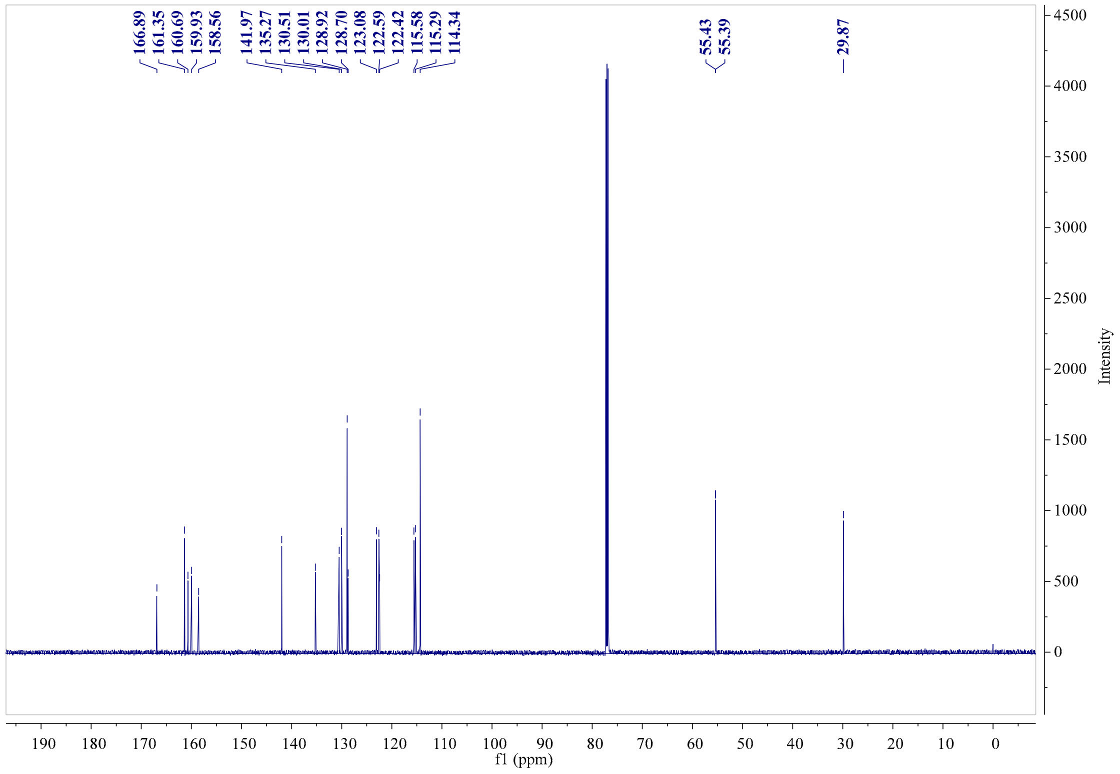

Figure S27. ^{13}C-NMR spectrum of compound **5f**.

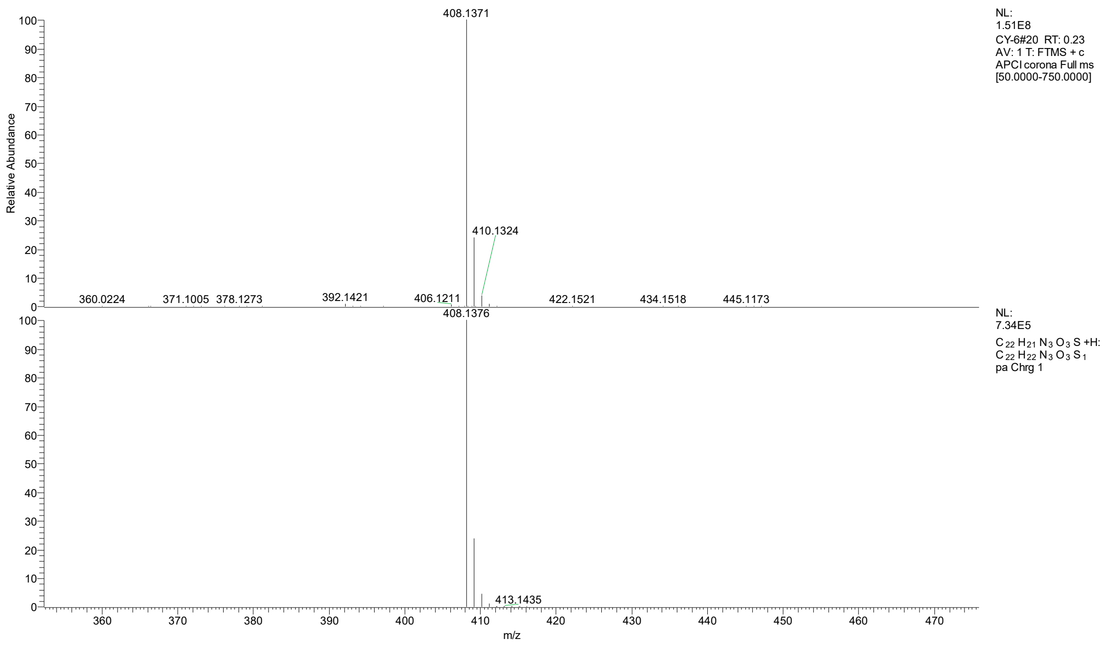

Figure S28. HRMS spectrum of compound **5f**.

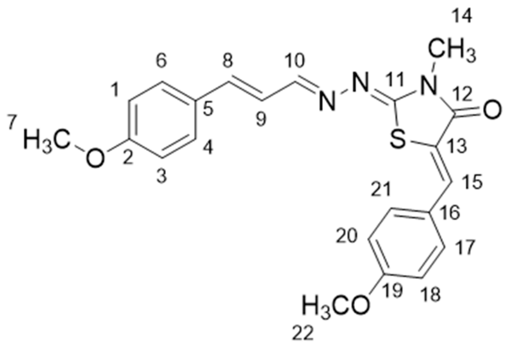

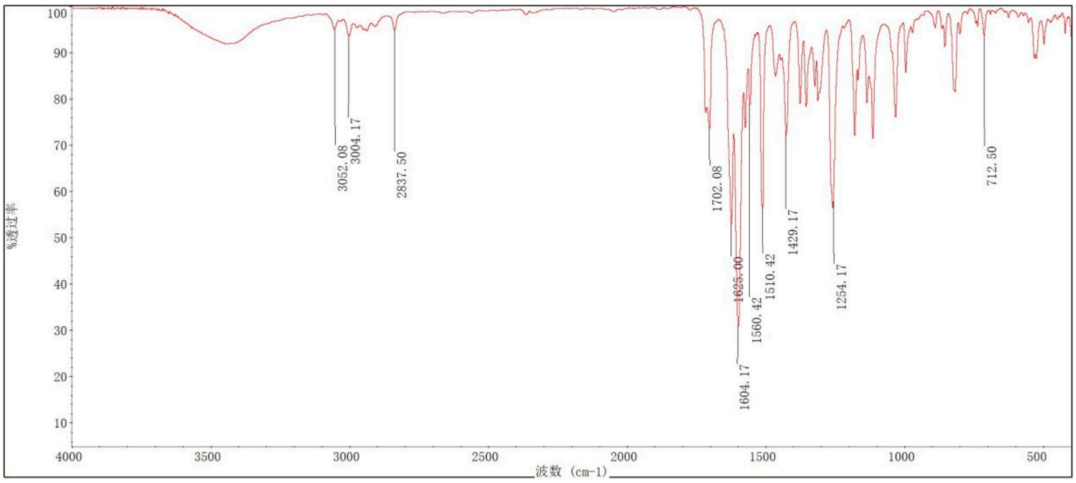

Figure S29. FT-IR spectrum of compound **5g**.

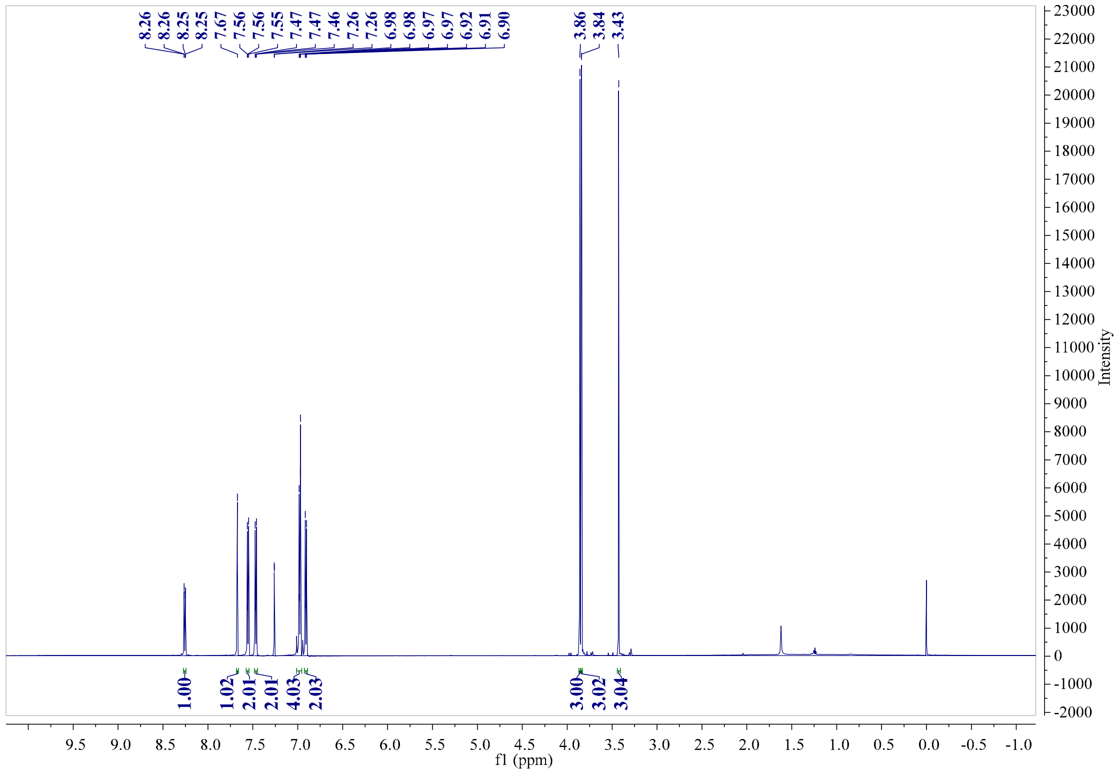

Figure S30. ^{1}H-NMR spectrum of compound **5g**.

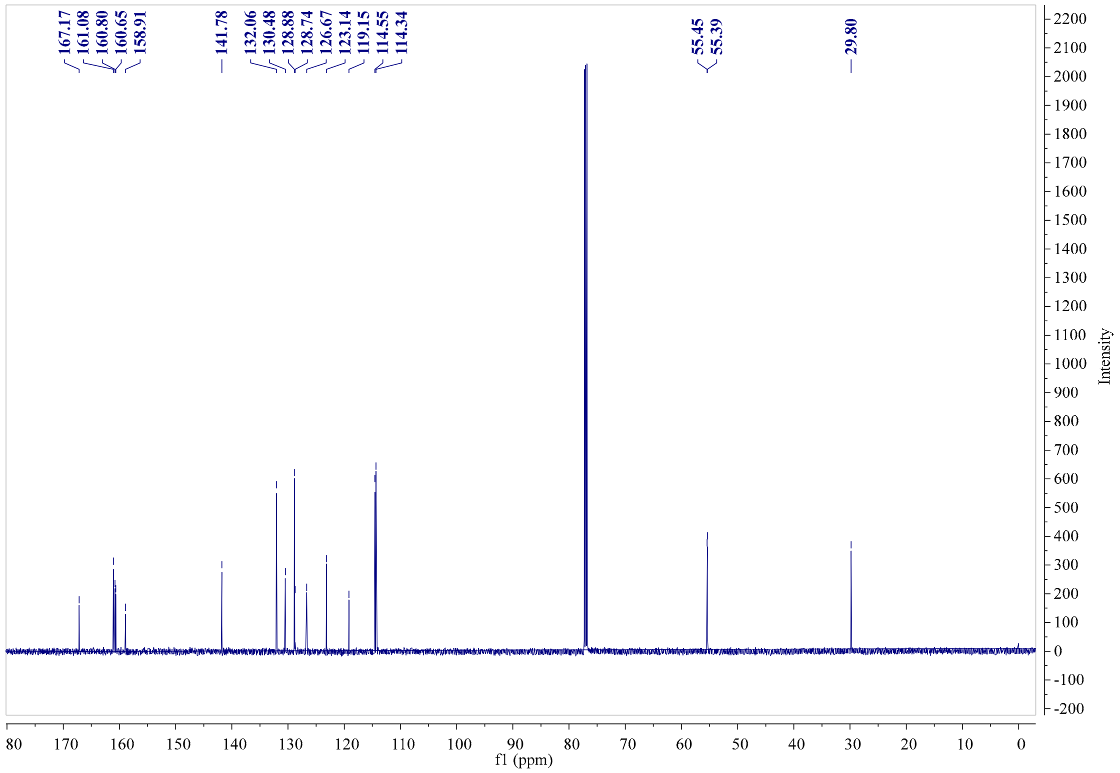

Figure S31. ^{13}C-NMR spectrum of compound **5g**.

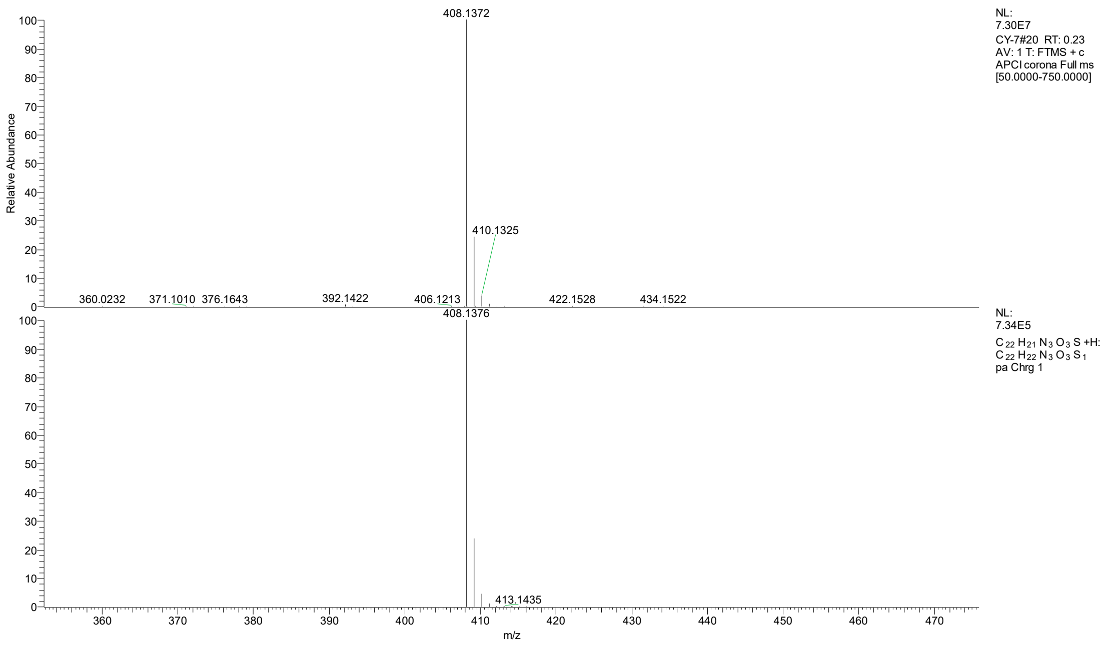

Figure S32. HRMS spectrum of compound **5g**.

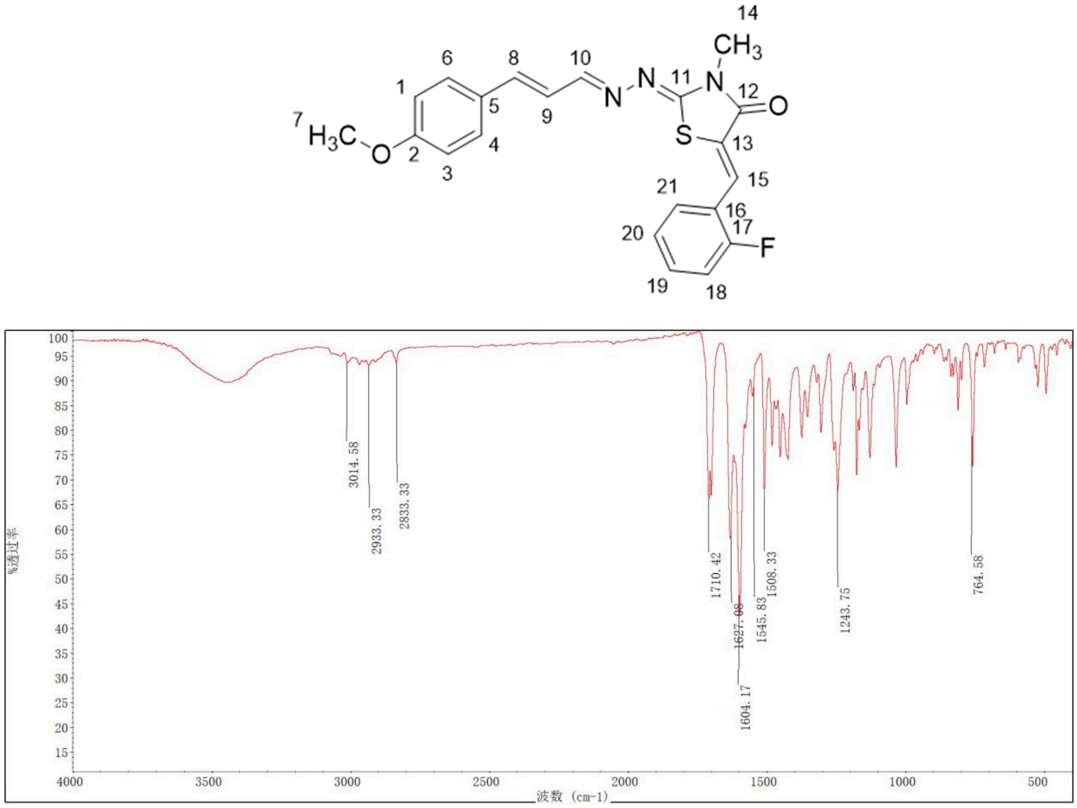

Figure S33. FT-IR spectrum of compound **5h**.

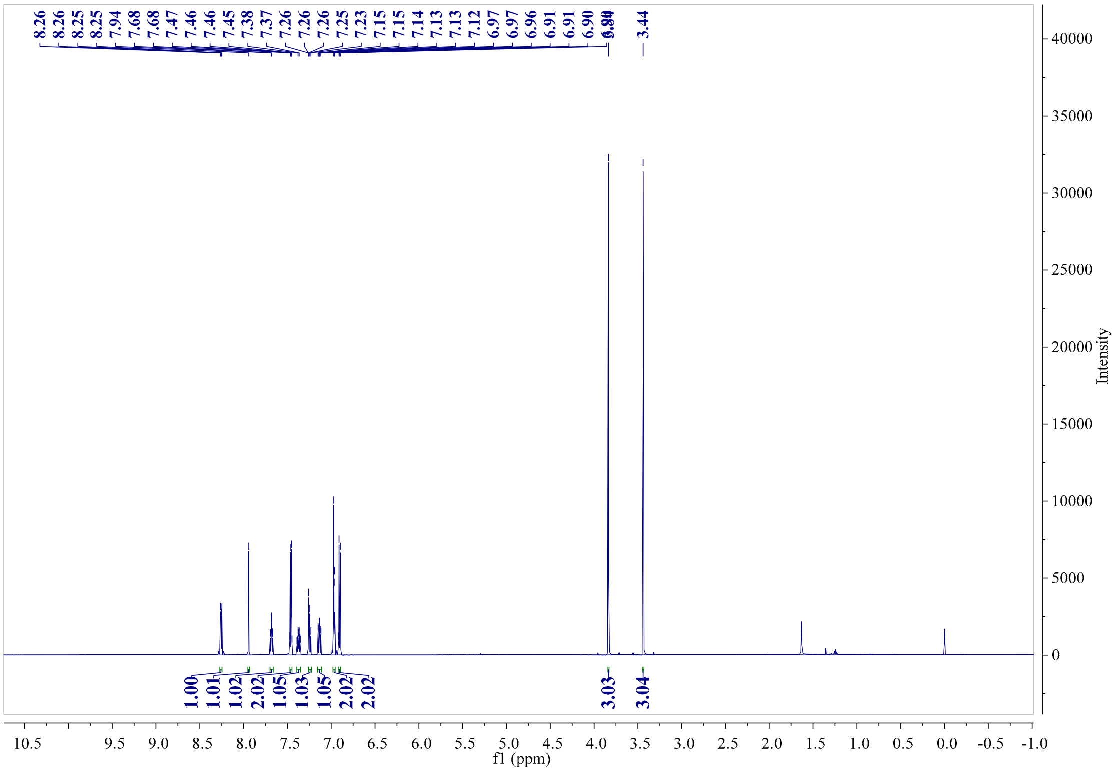

Figure S34. ^{1}H-NMR spectrum of compound **5h**.

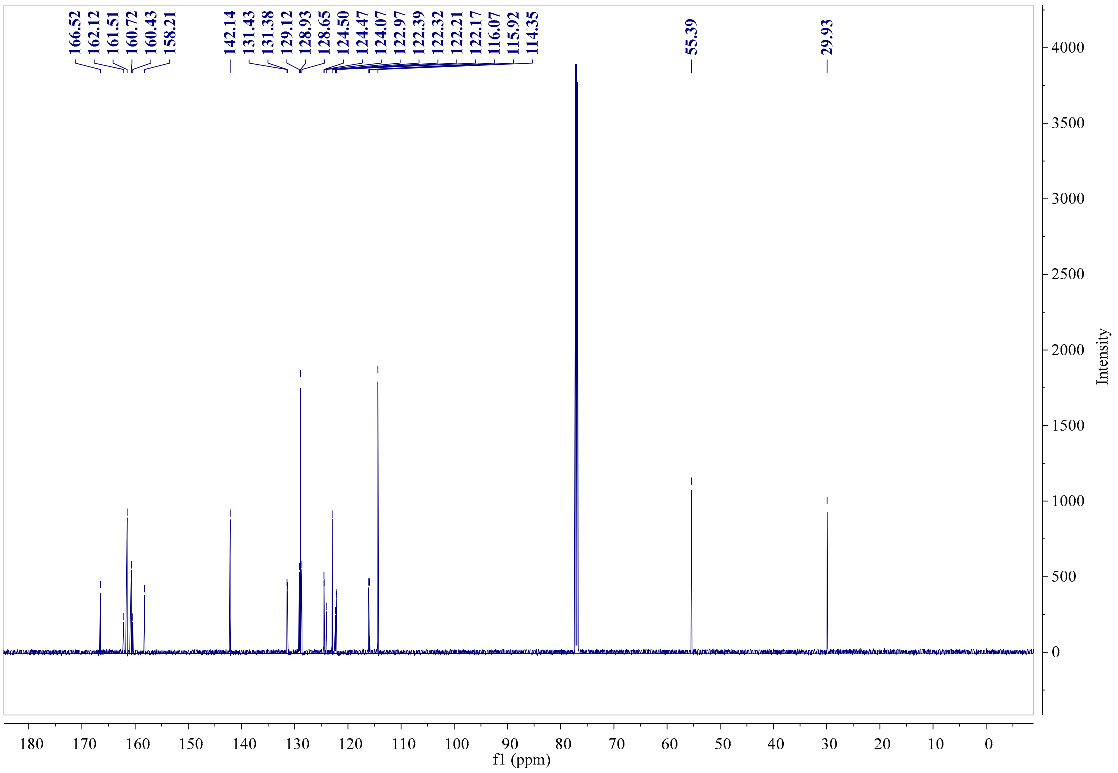

Figure S35. ^{13}C-NMR spectrum of compound **5h**.

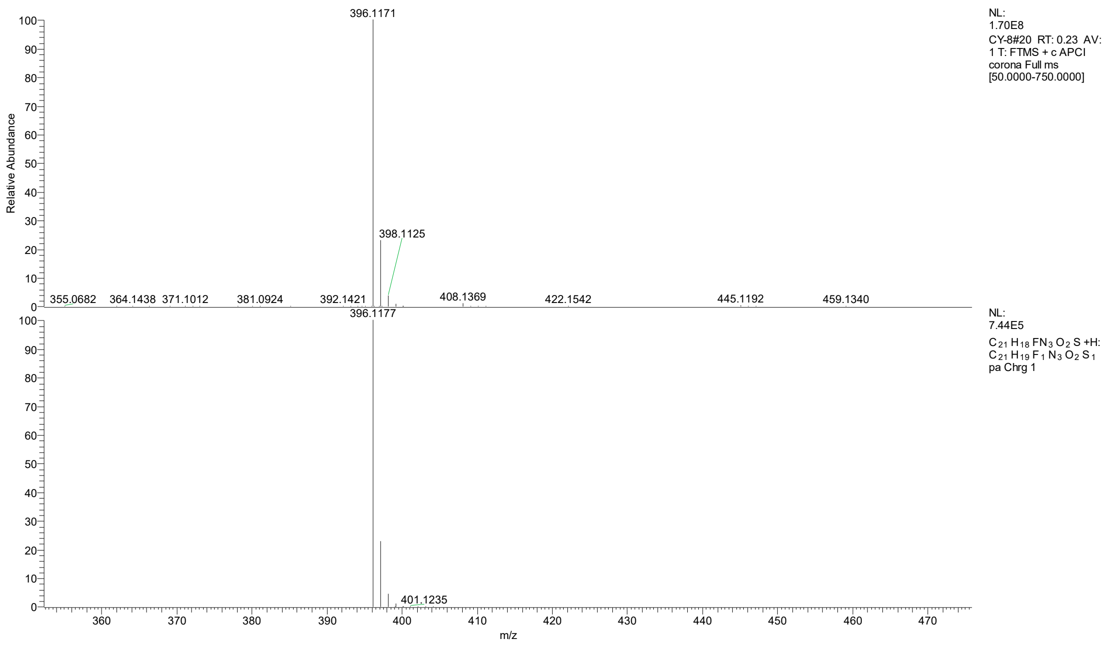

Figure S36. HRMS spectrum of compound **5h**.

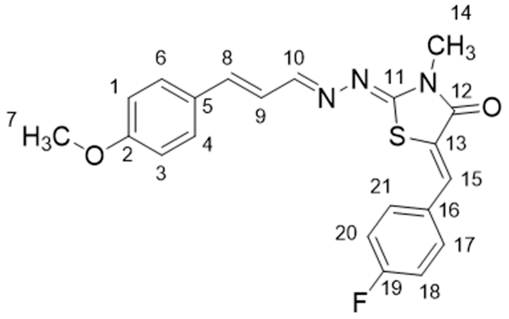

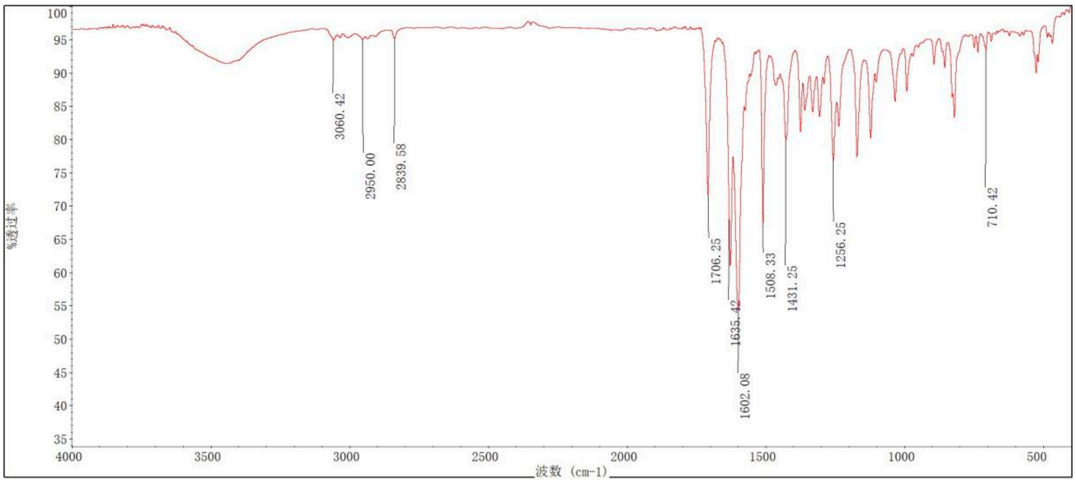

Figure S37. FT-IR spectrum of compound **5i**.

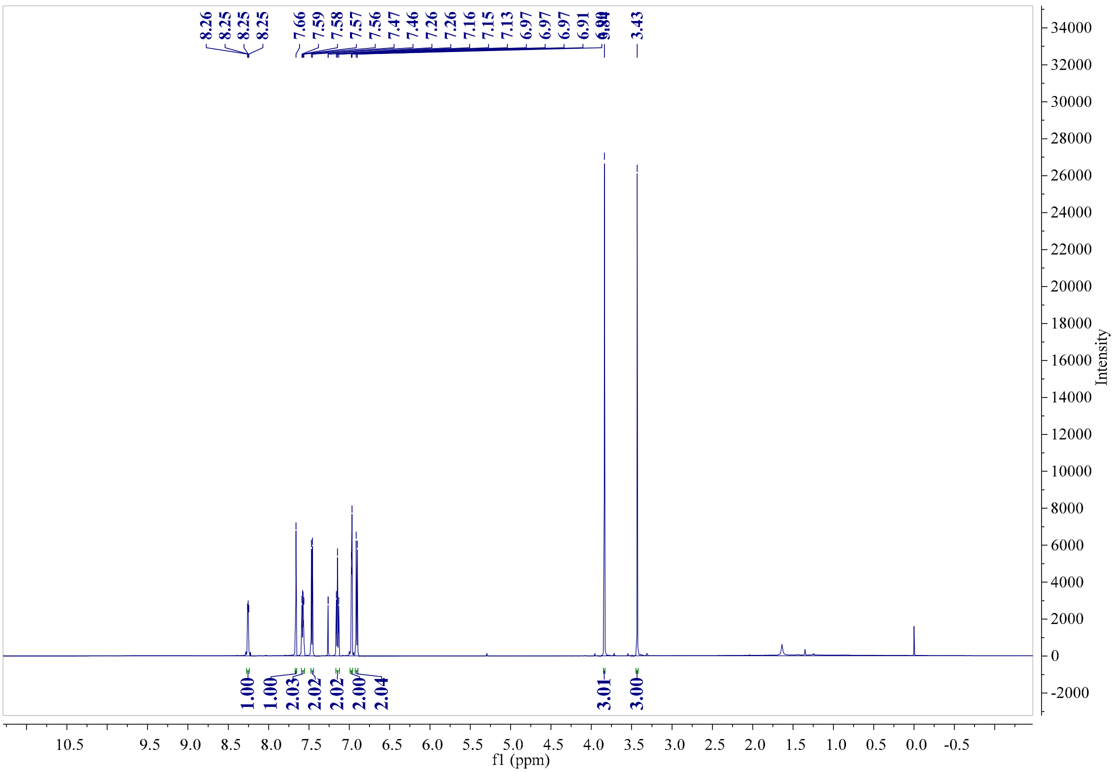

Figure S38. ^{1}H-NMR spectrum of compound **5i**.

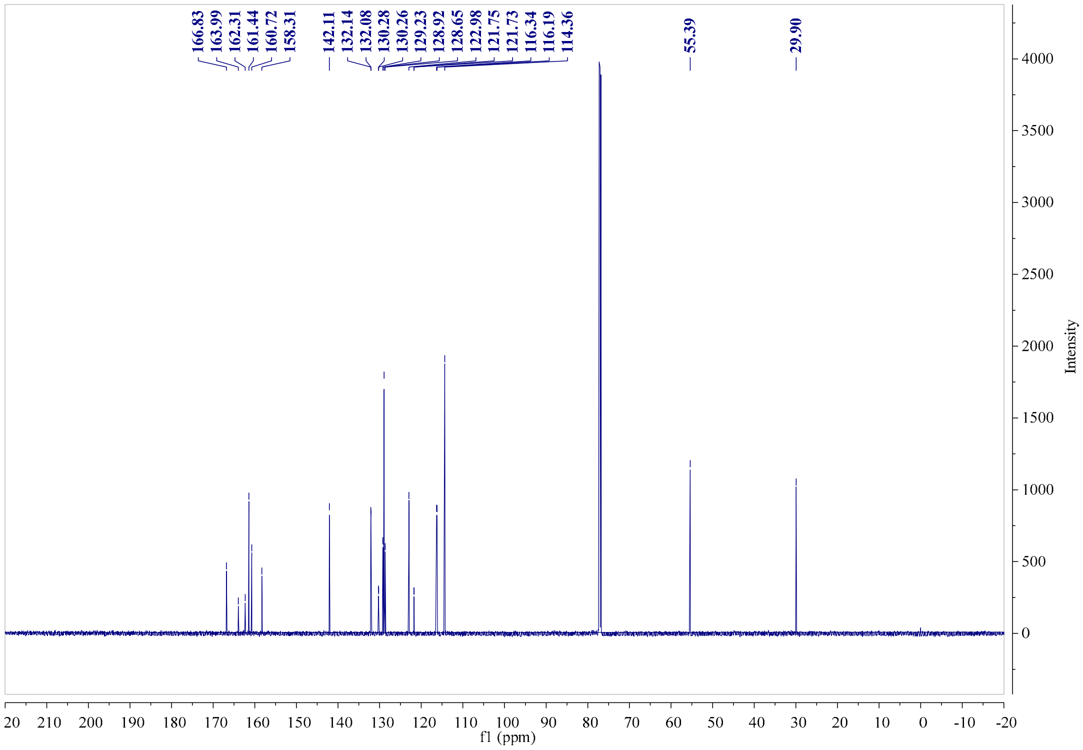

Figure S39. ^{13}C-NMR spectrum of compound **5i**.

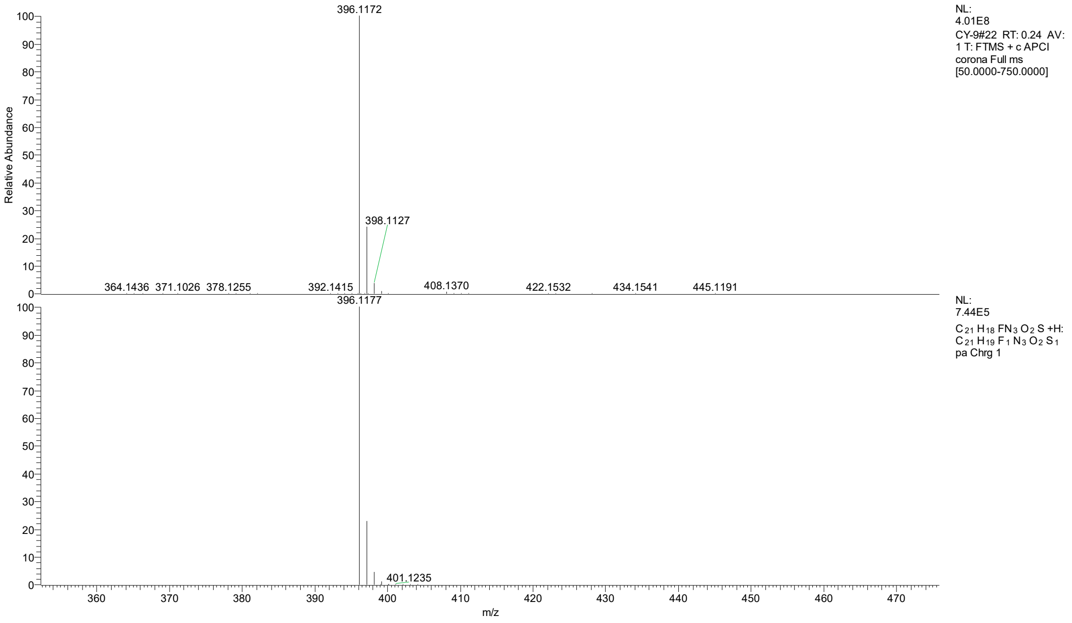

Figure S40. HRMS spectrum of compound **5i**.

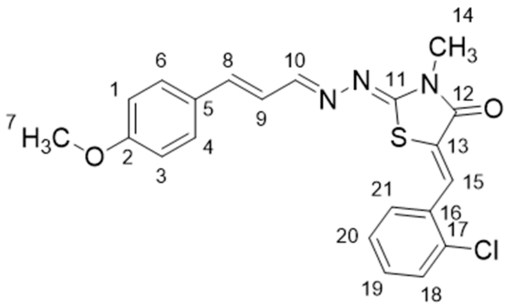

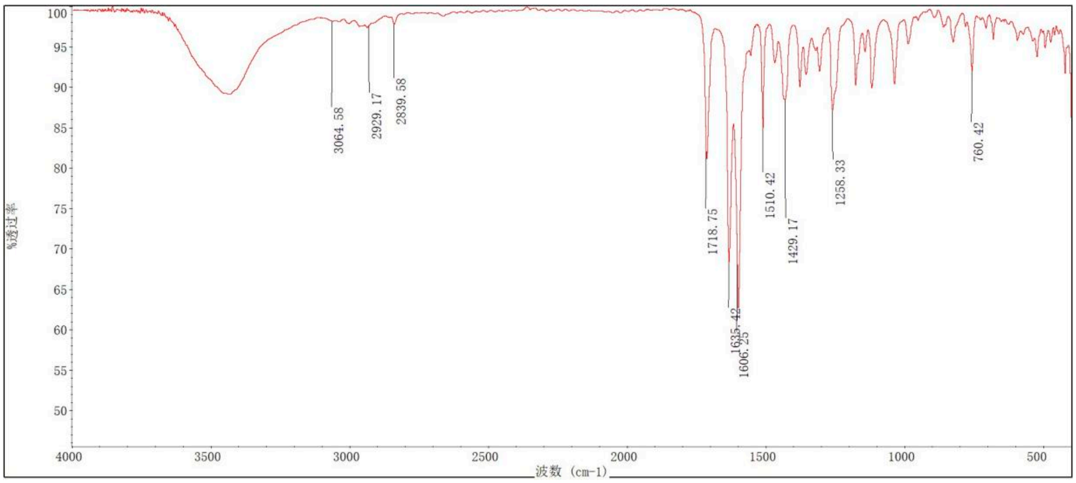

Figure S41. FT-IR spectrum of compound **5j**.

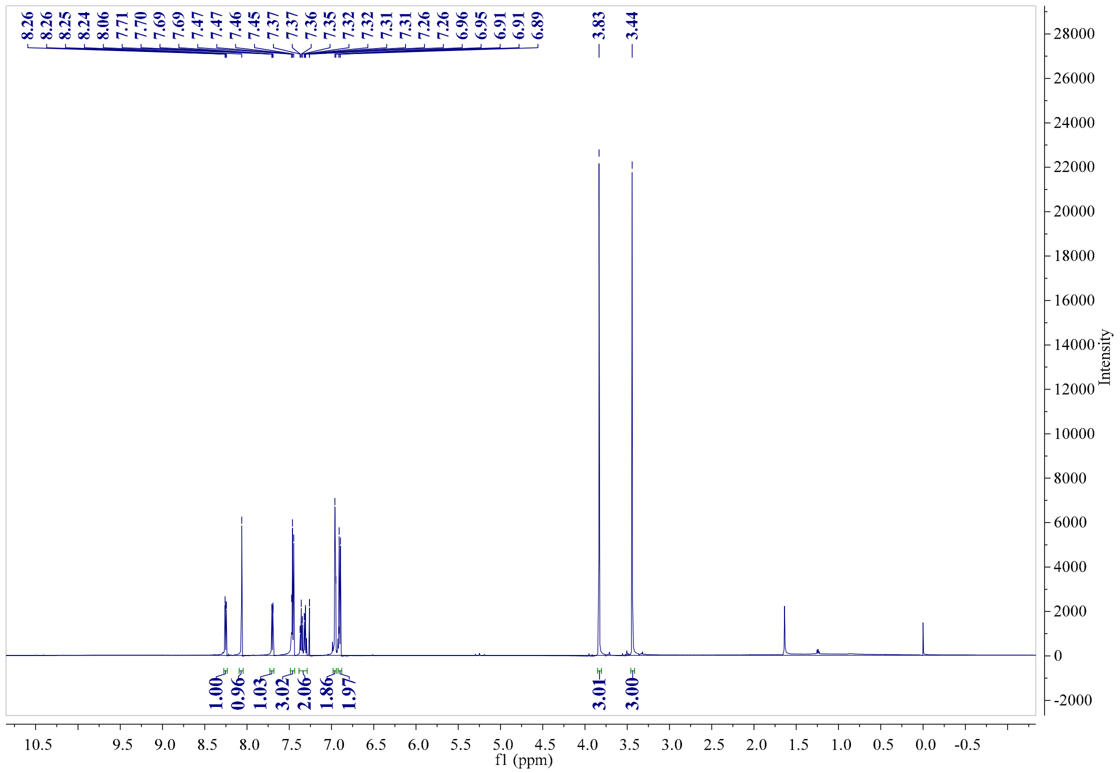

Figure S42. ^{1}H-NMR spectrum of compound **5j**.

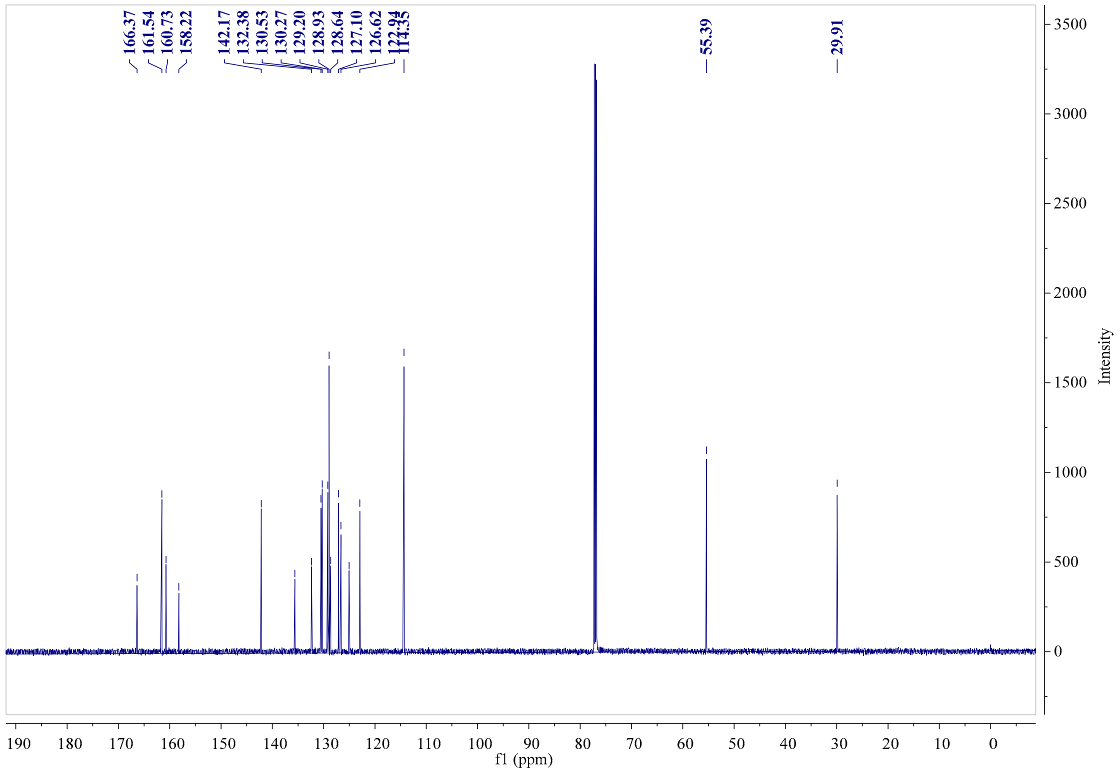

Figure S43. ^{13}C-NMR spectrum of compound **5j**.

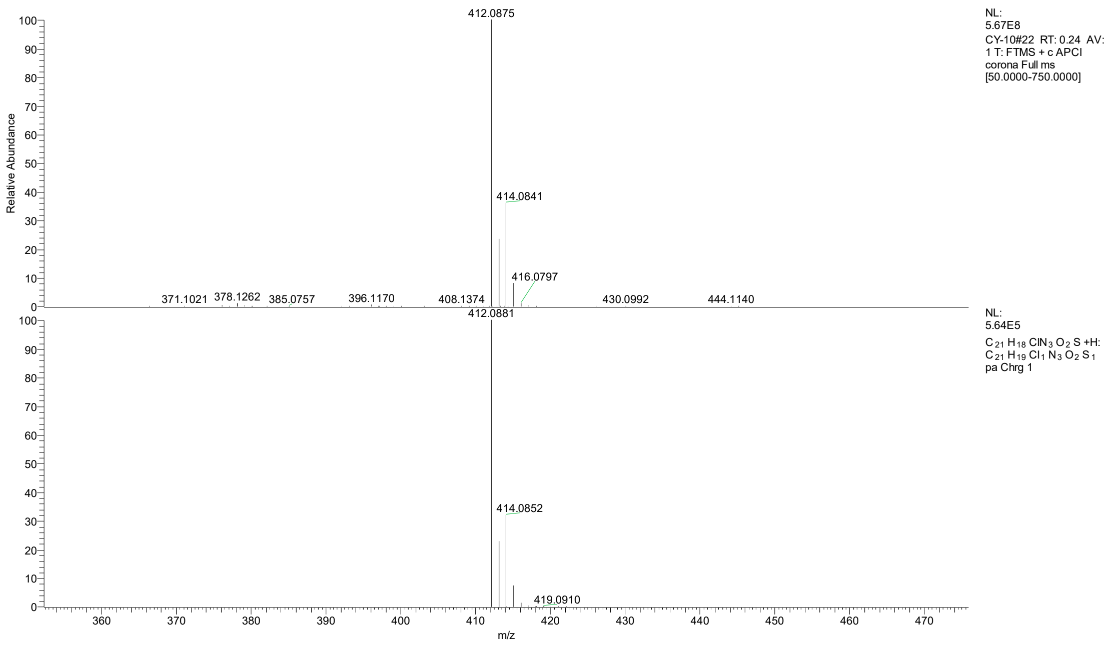

Figure S44. HRMS spectrum of compound **5j**.

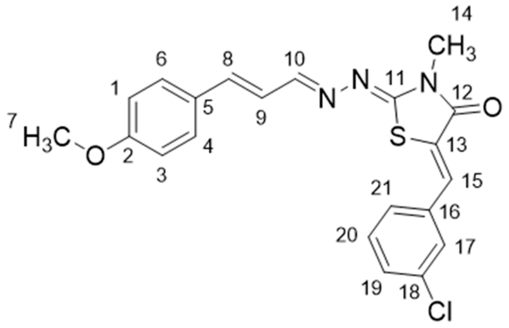

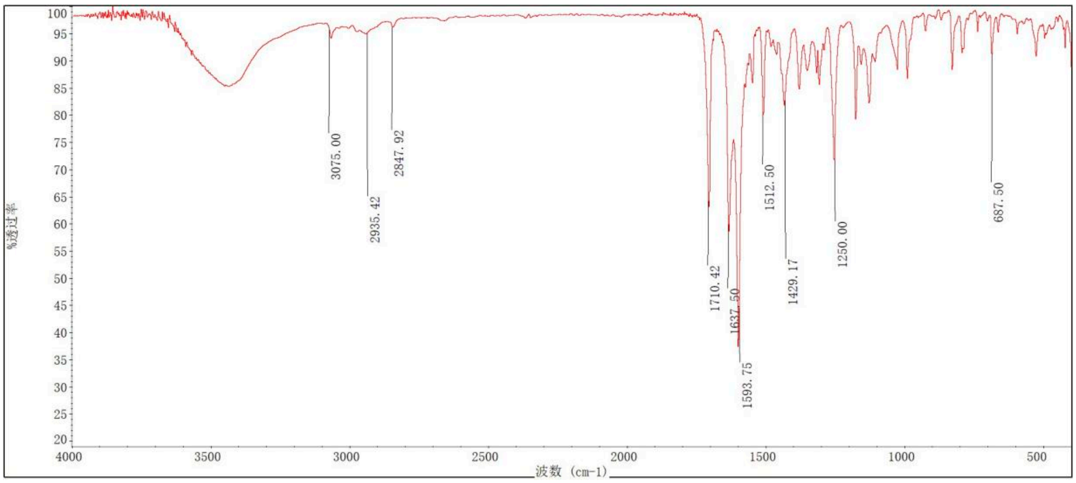

Figure S45. FT-IR spectrum of compound **5k**.

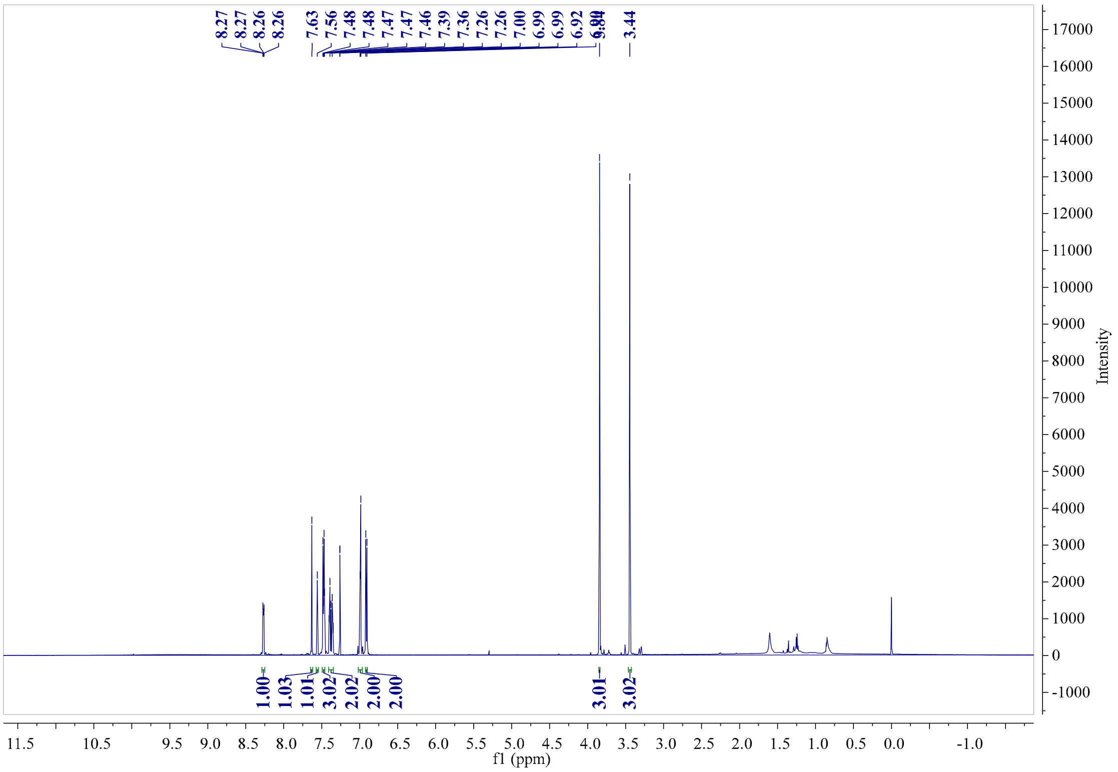

Figure S46. ^{1}H-NMR spectrum of compound **5k**.

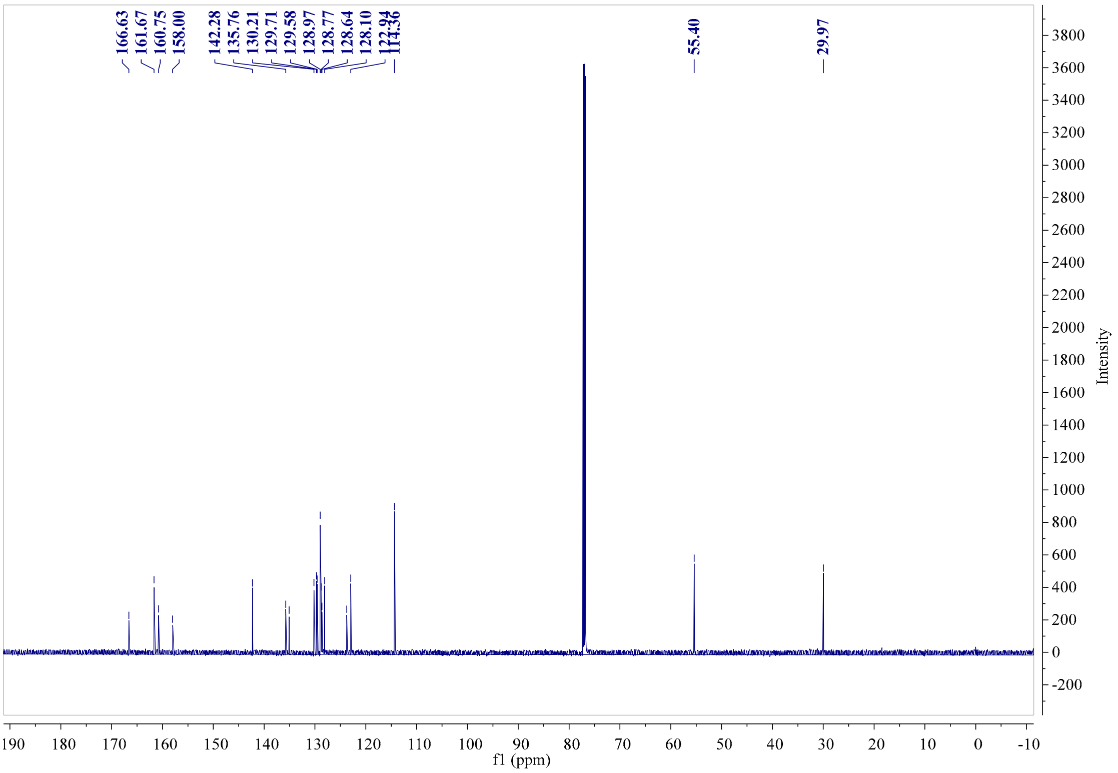

Figure S47. ^{13}C-NMR spectrum of compound **5k**.

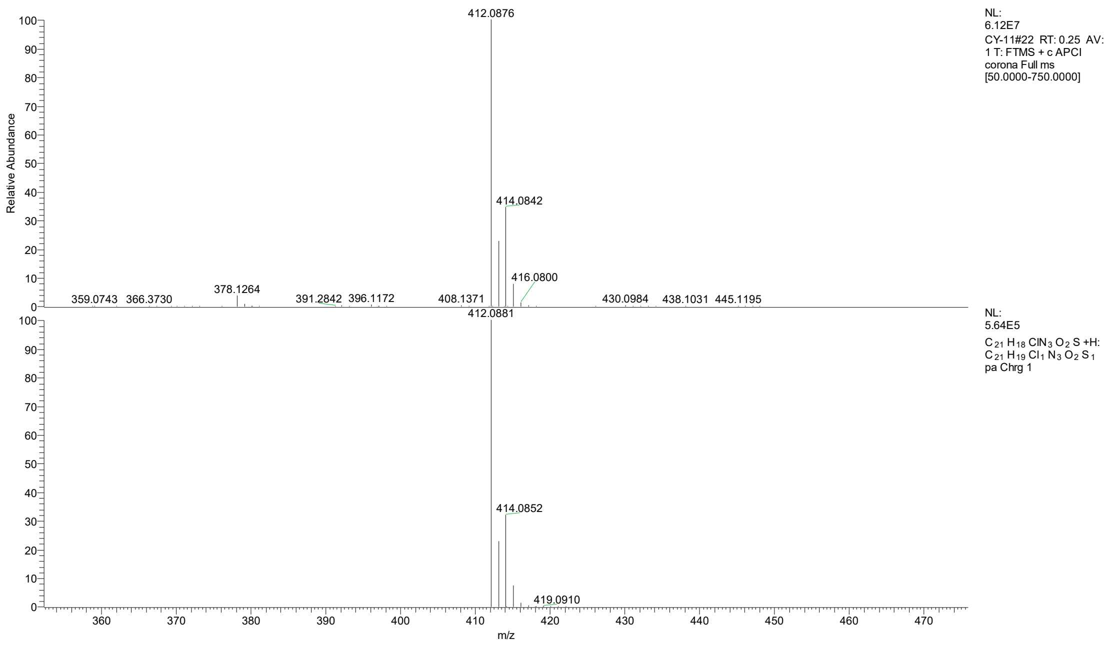

Figure S48. HRMS spectrum of compound **5k**.

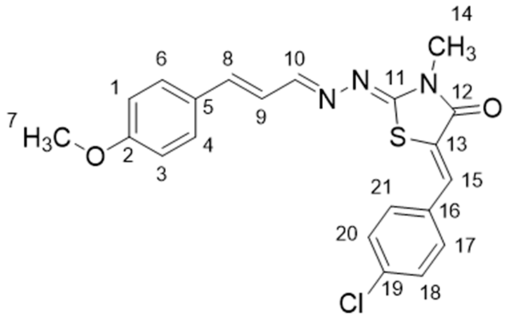

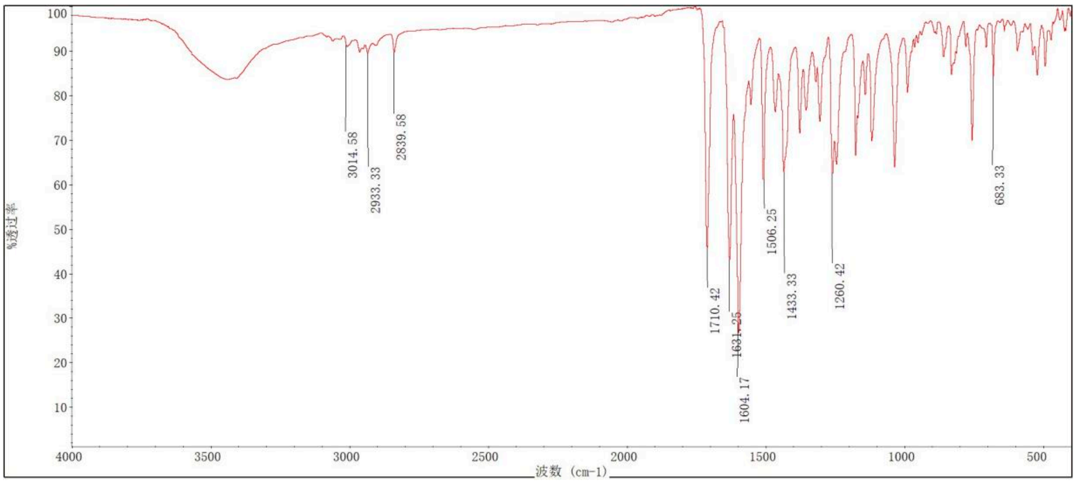

Figure S49. FT-IR spectrum of compound **5l**.

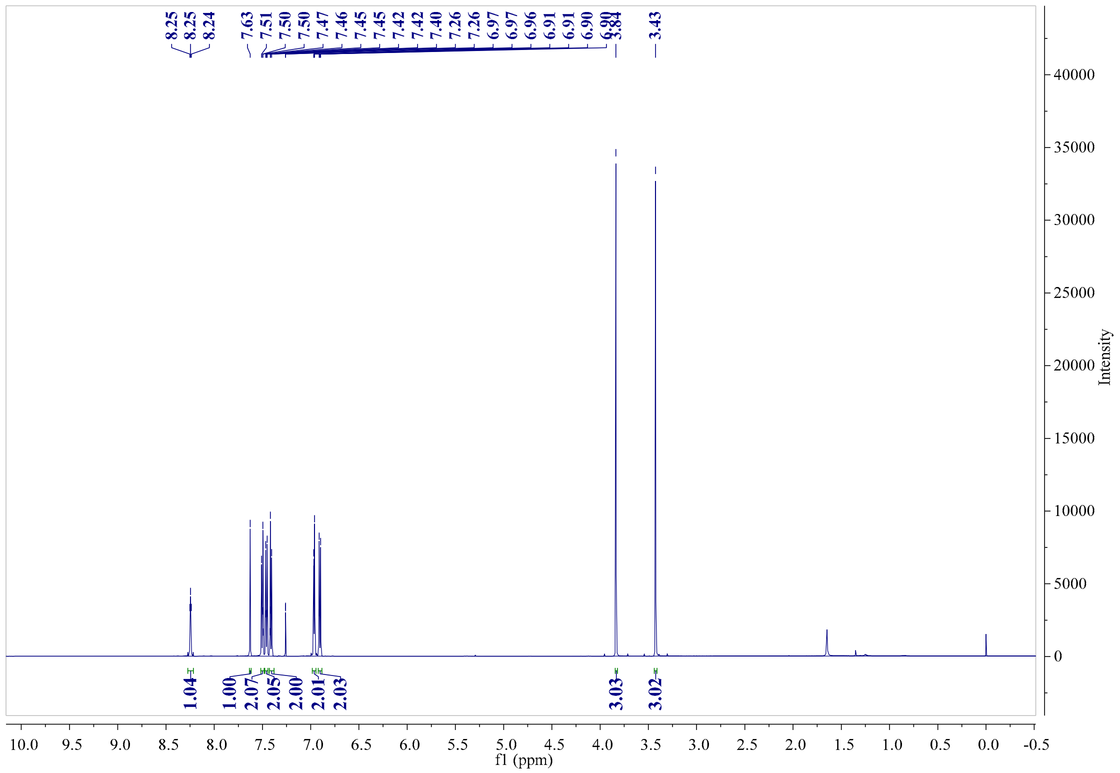

Figure S50. ^{1}H-NMR spectrum of compound **5l**.

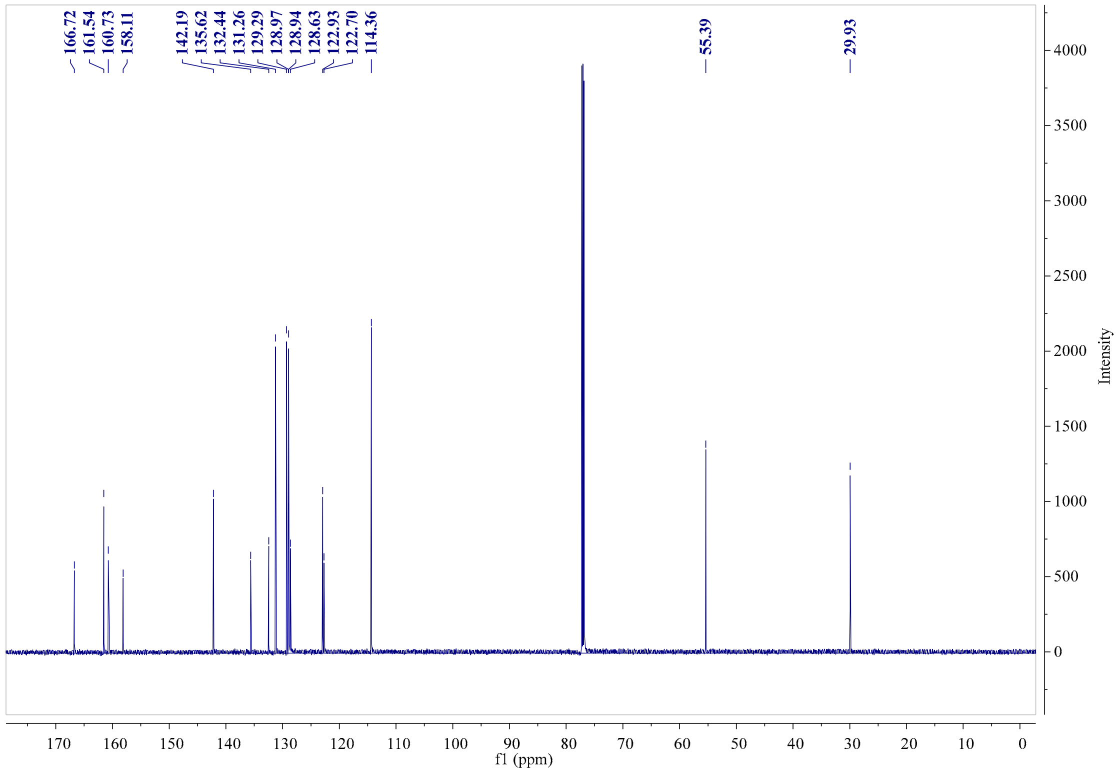

Figure S51. ^{13}C-NMR spectrum of compound **5l**.

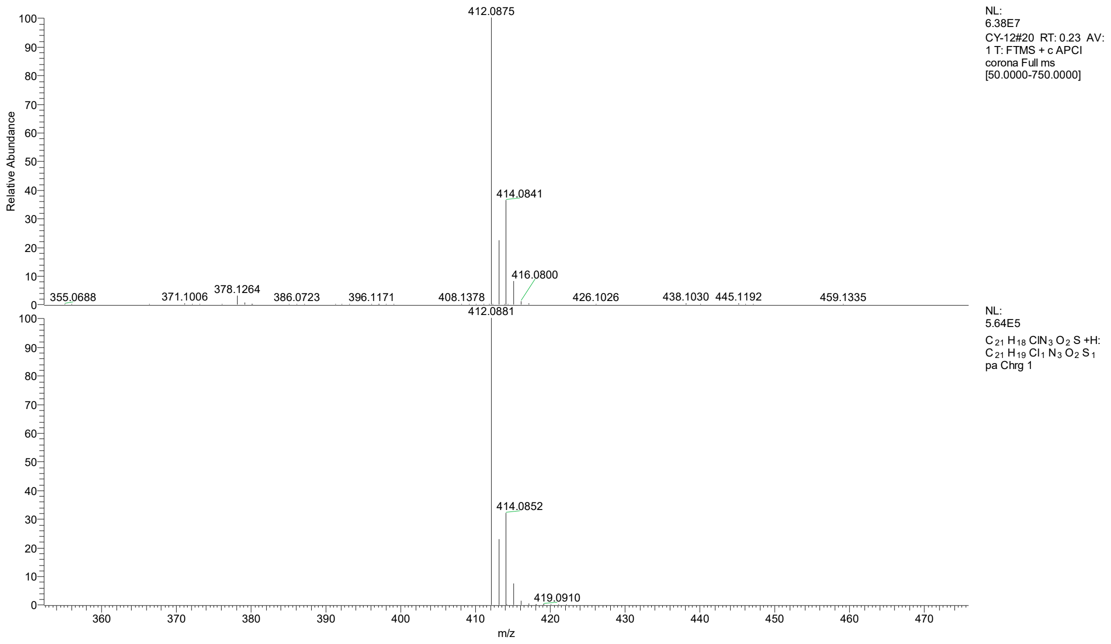

Figure S52. HRMS spectrum of compound **5l**.

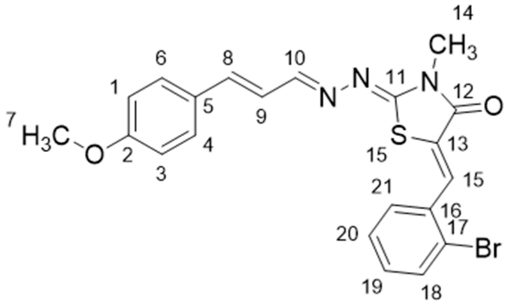

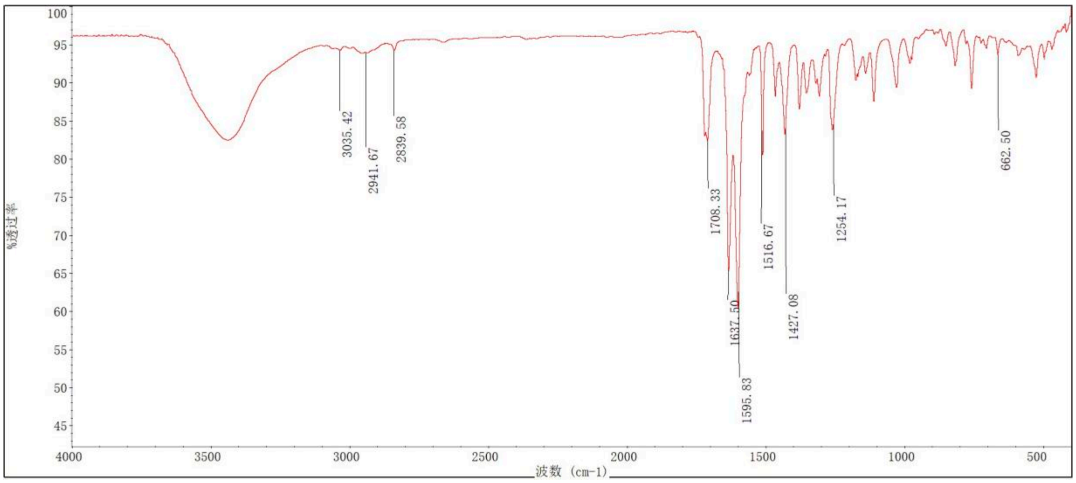

Figure S53. FT-IR spectrum of compound **5m**.

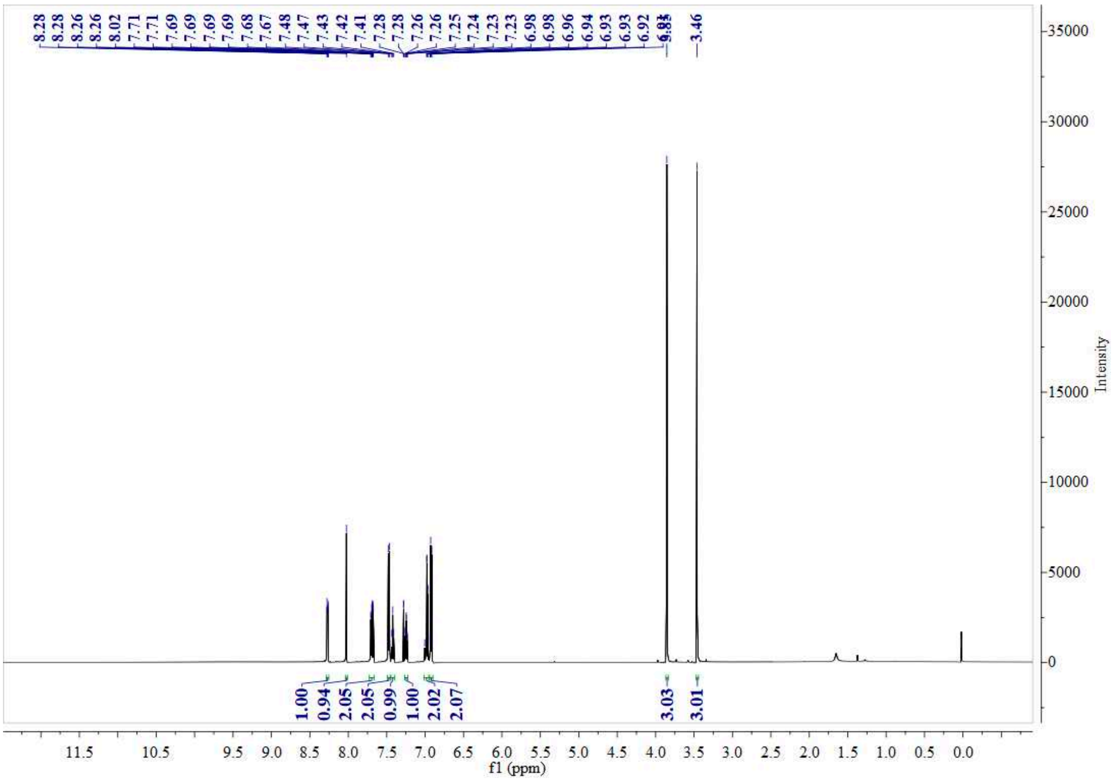

Figure S54. ^{1}H-NMR spectrum of compound **5m**.

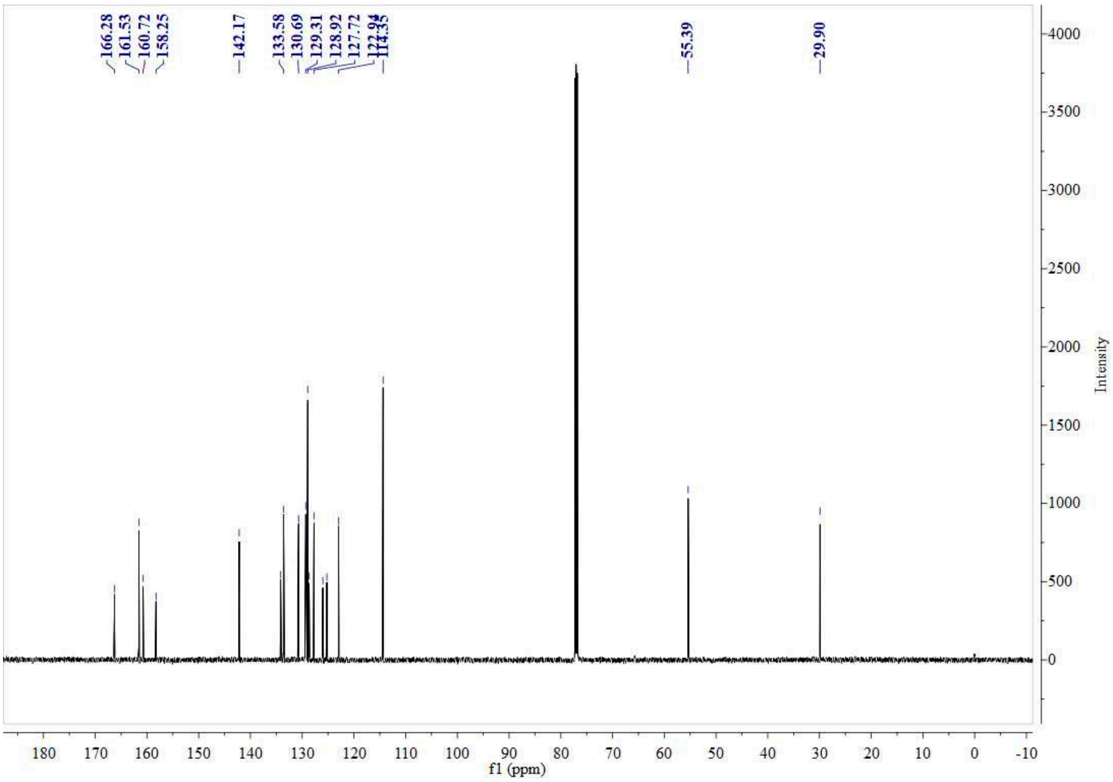

Figure S55. ^{13}C-NMR spectrum of compound **5m**.

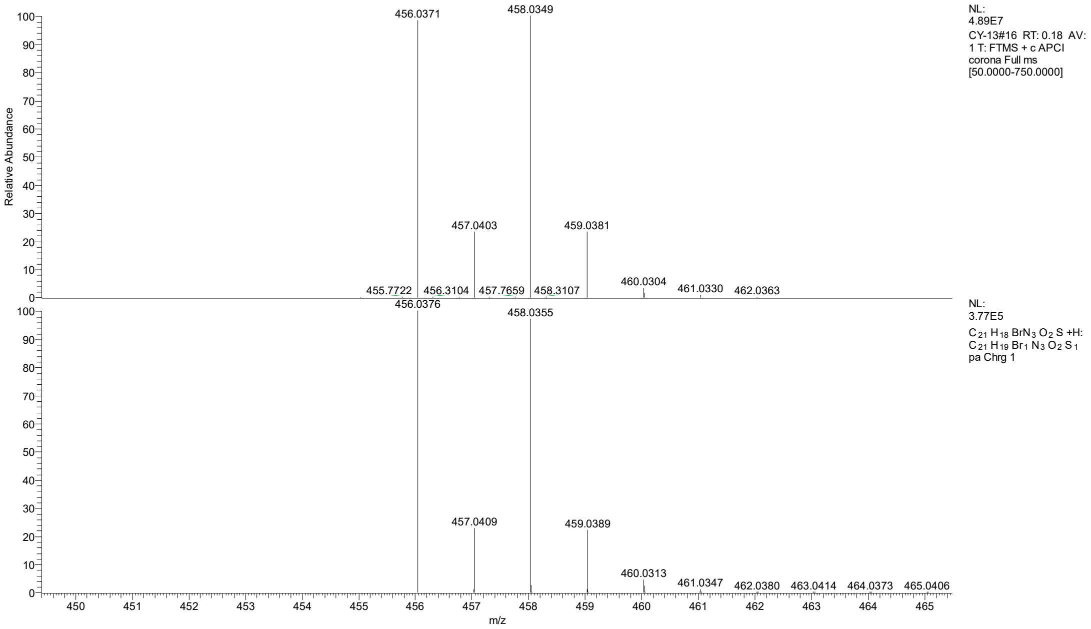

Figure S56. HRMS spectrum of compound **5m**.

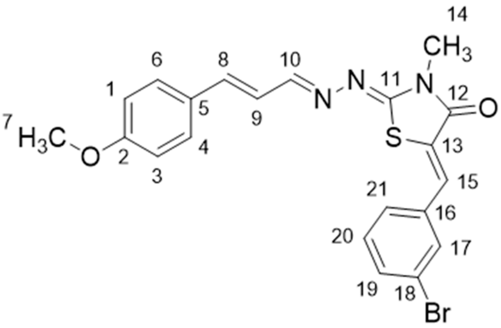

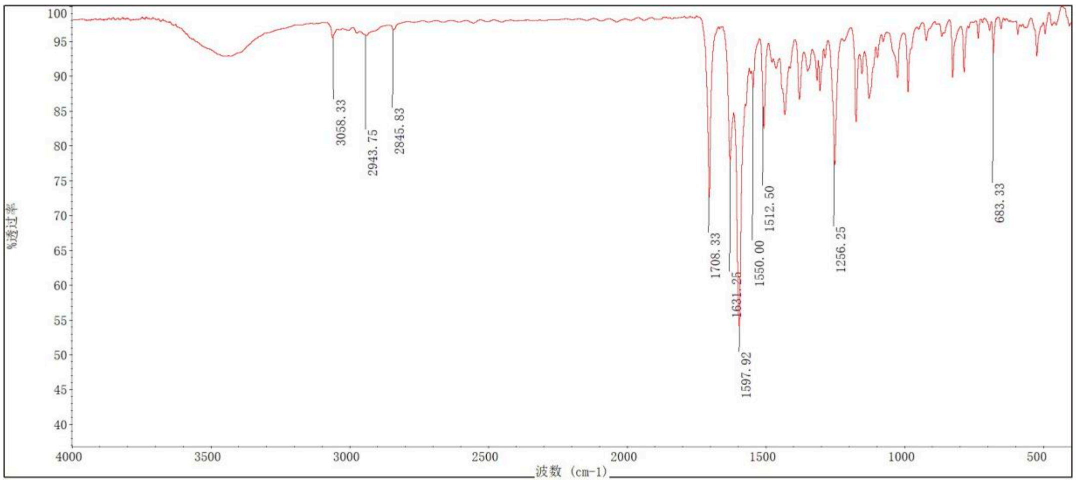

Figure S57. FT-IR spectrum of compound **5n.**

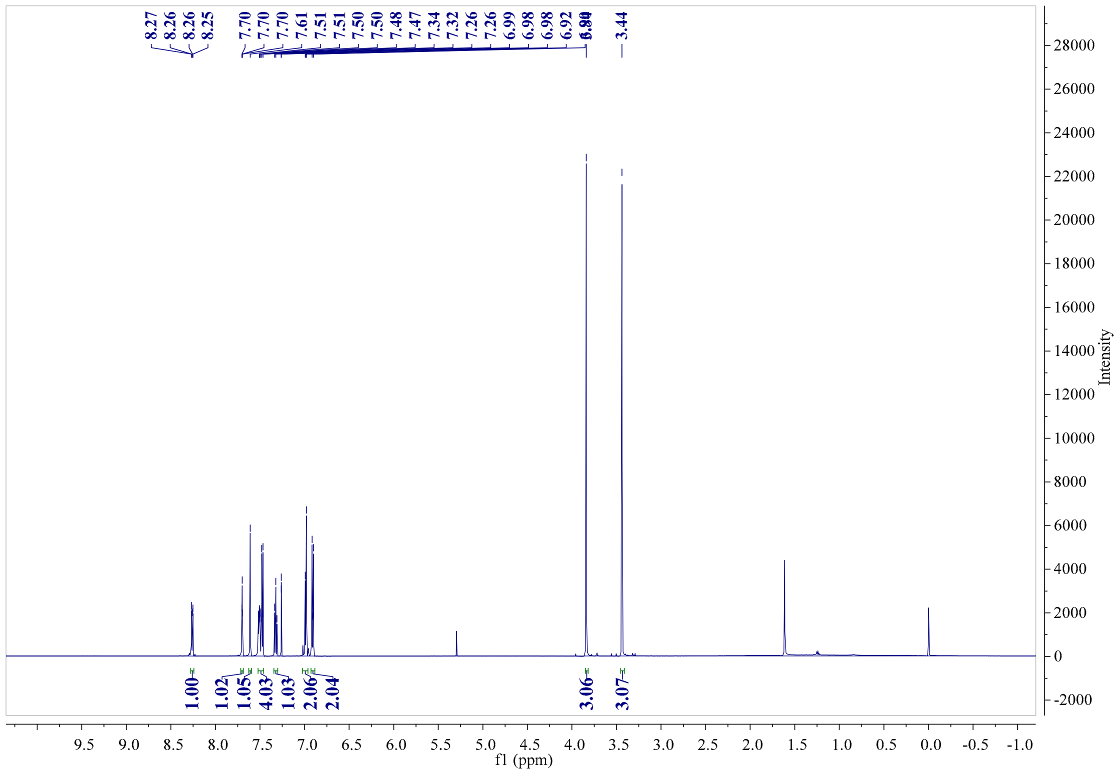

Figure S58. ^{1}H-NMR spectrum of compound **5n.**

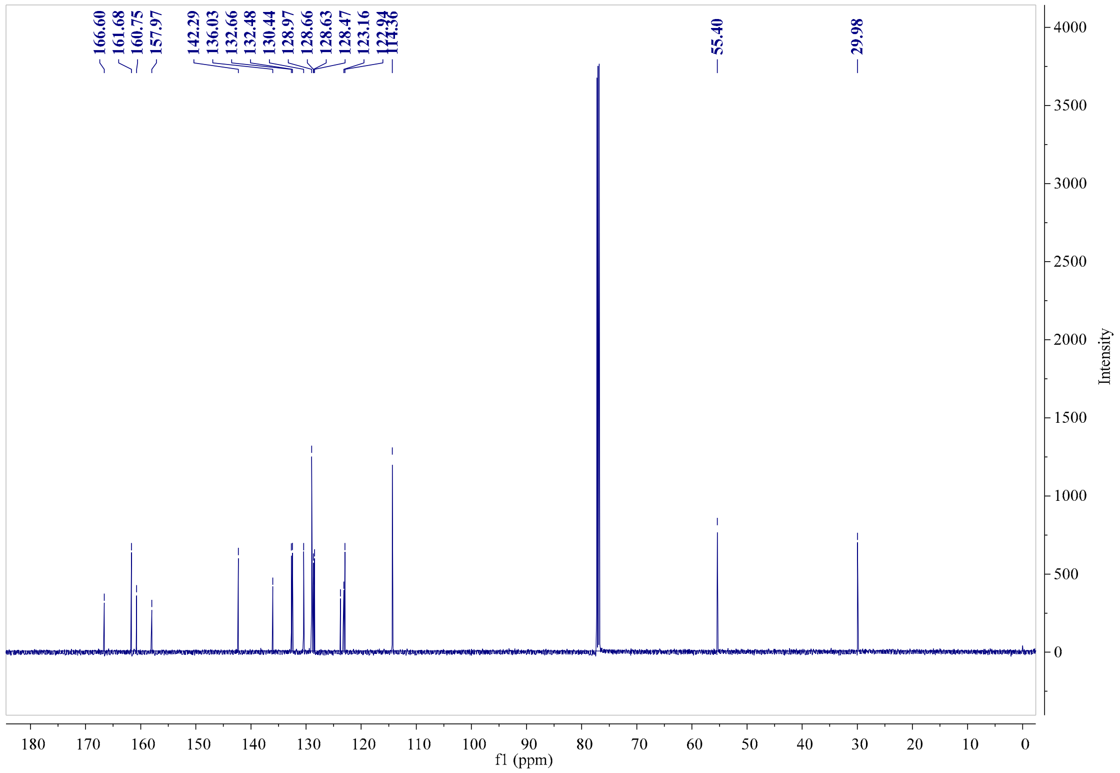

Figure S59. ^{13}C-NMR spectrum of compound **5n**.

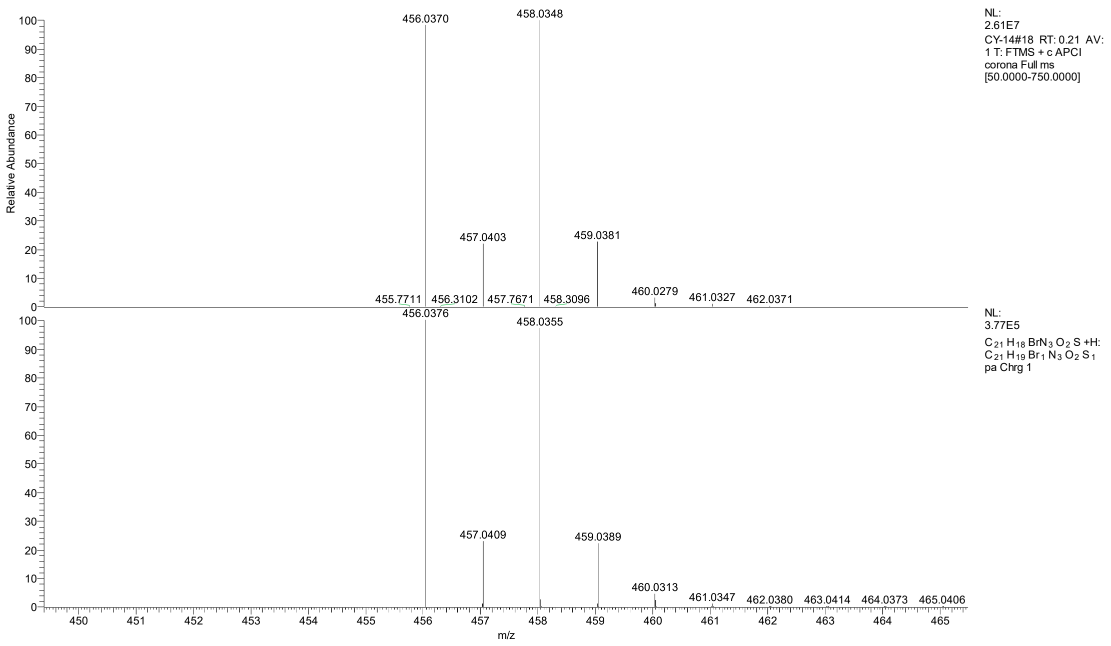

Figure S60. HRMS spectrum of compound **5n**.

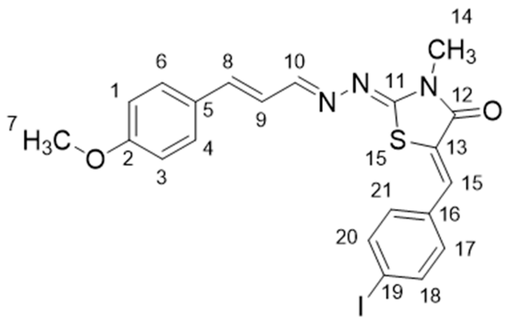

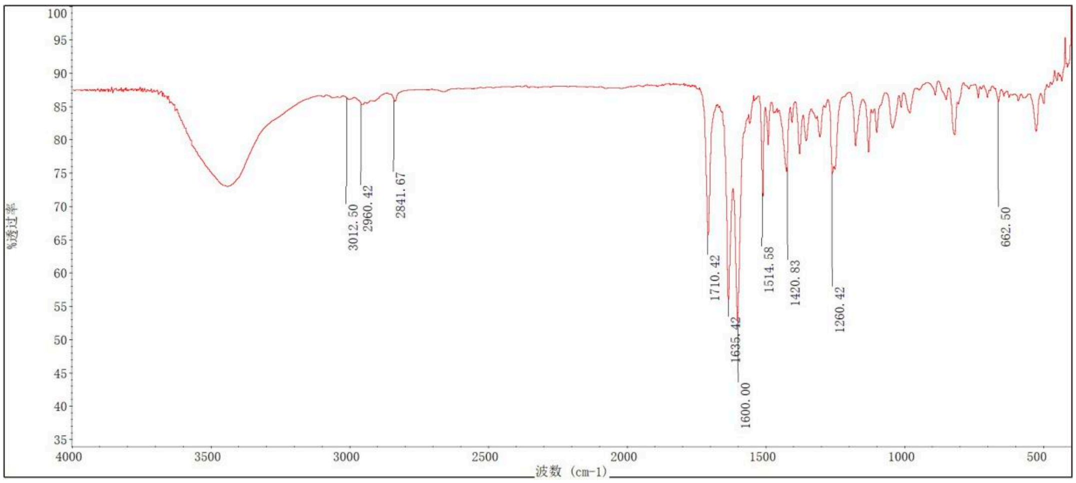

Figure S61. FT-IR spectrum of compound **5o**.

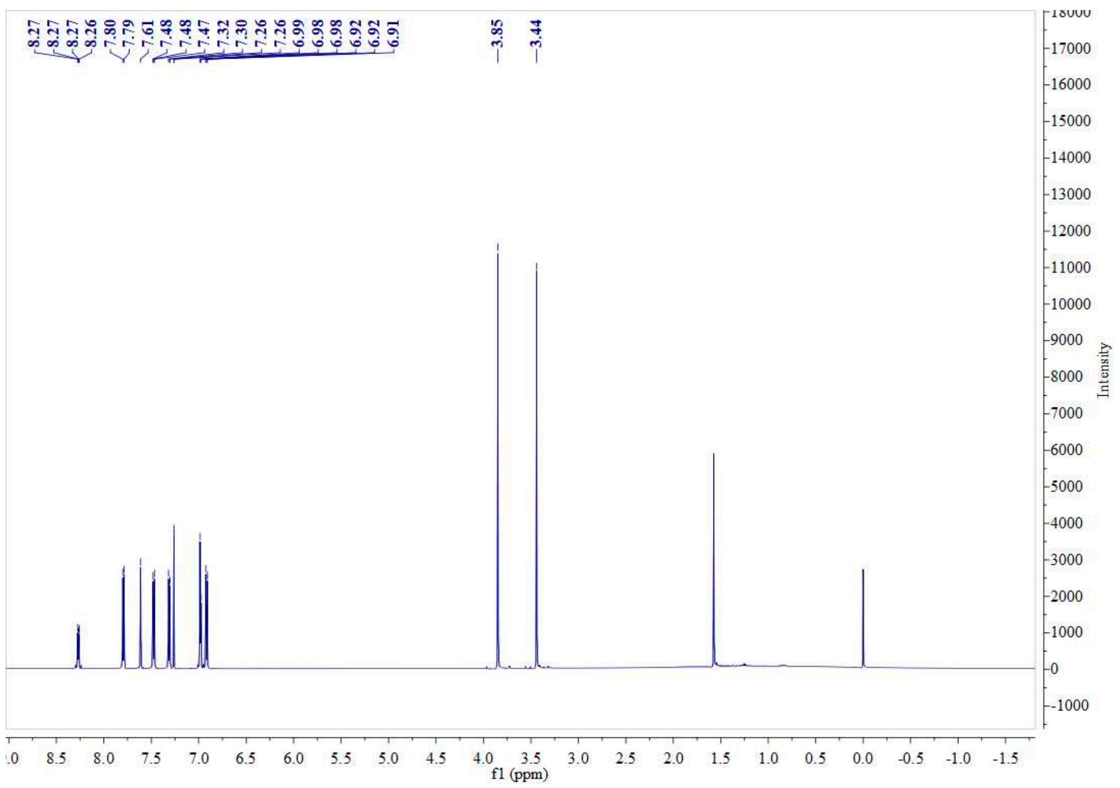

Figure S62. ^{1}H-NMR spectrum of compound **5o**.

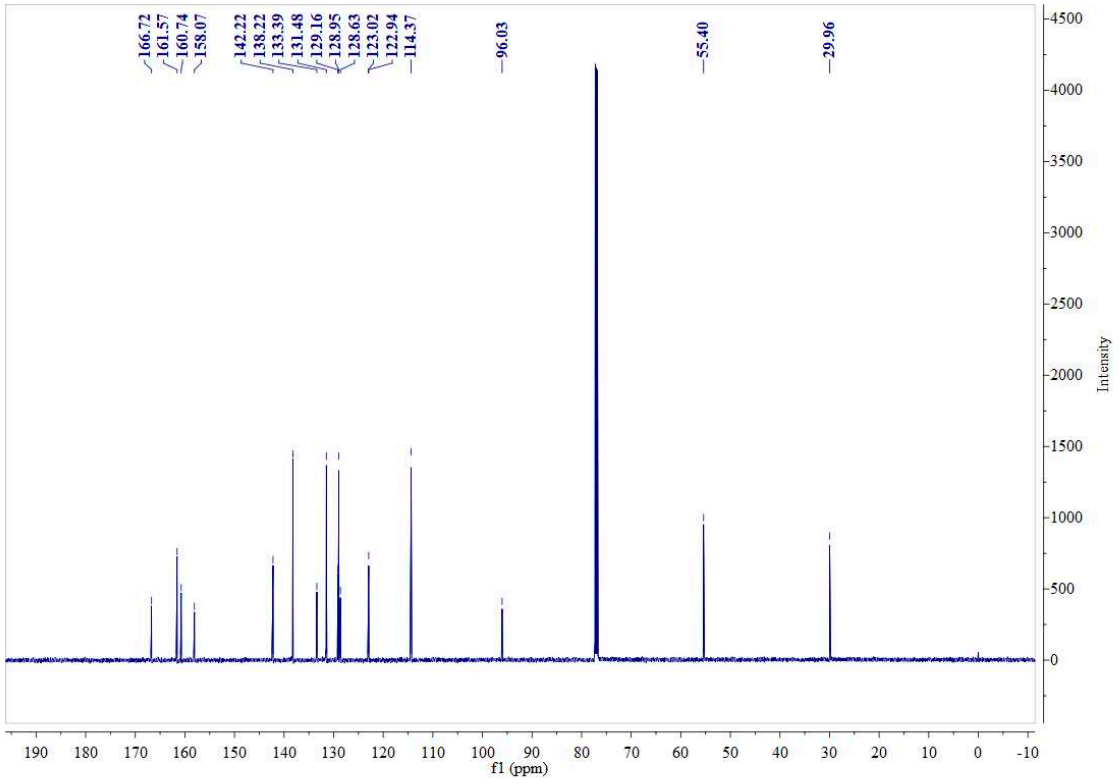

Figure S63. ^{13}C-NMR spectrum of compound **5o**.

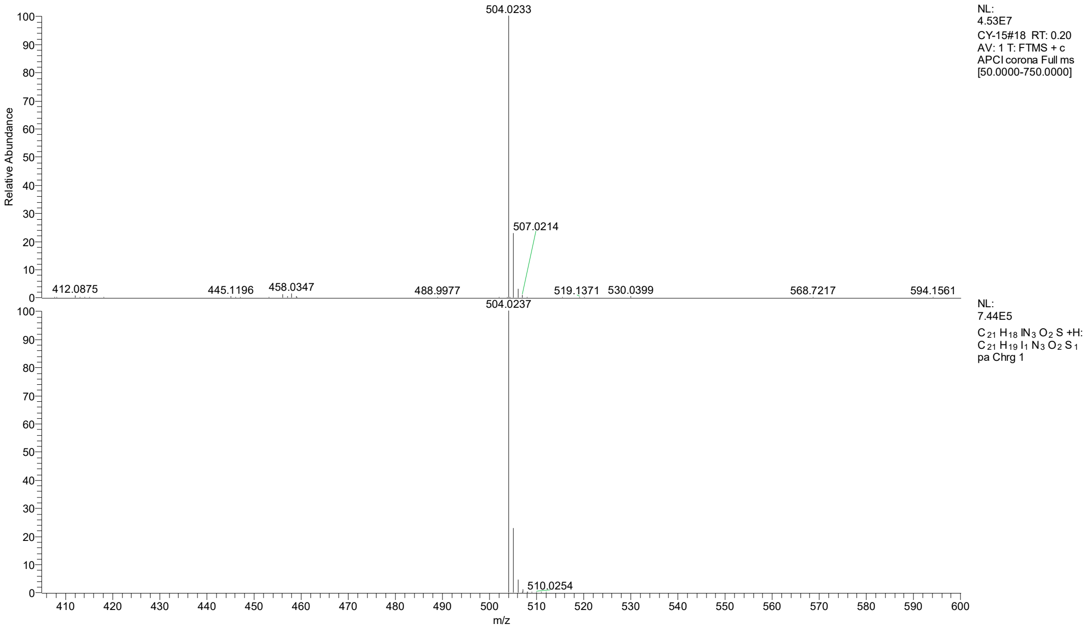

Figure S64. HRMS spectrum of compound **5o**.

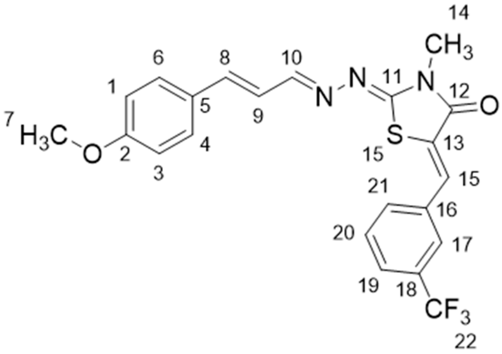

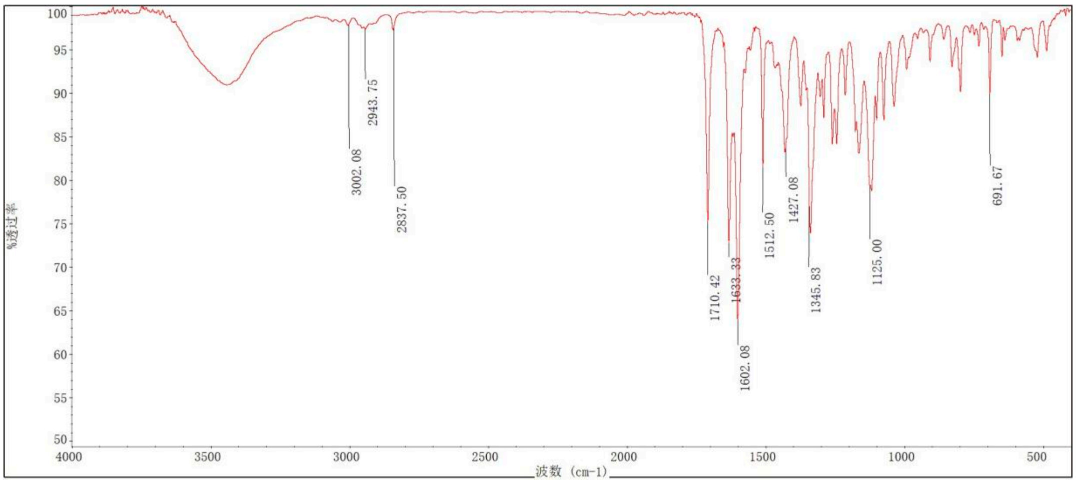

Figure S65. FT-IR spectrum of compound **5p**.

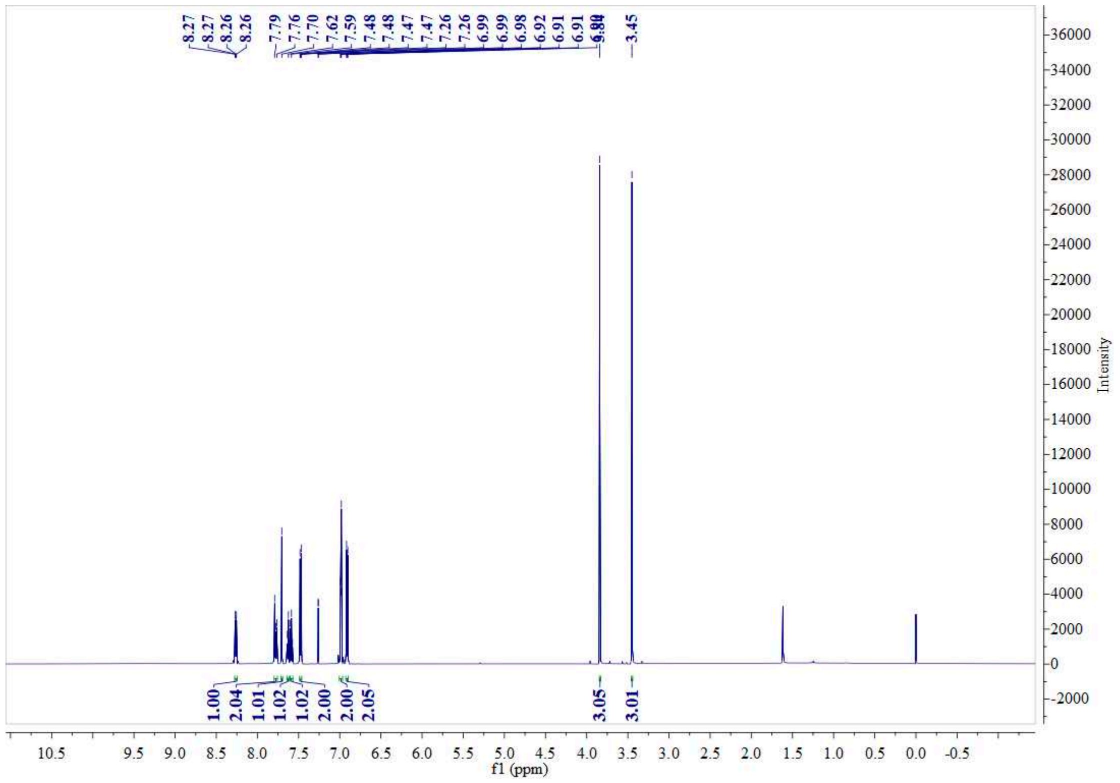

Figure S66. ^{1}H-NMR spectrum of compound **5p**.

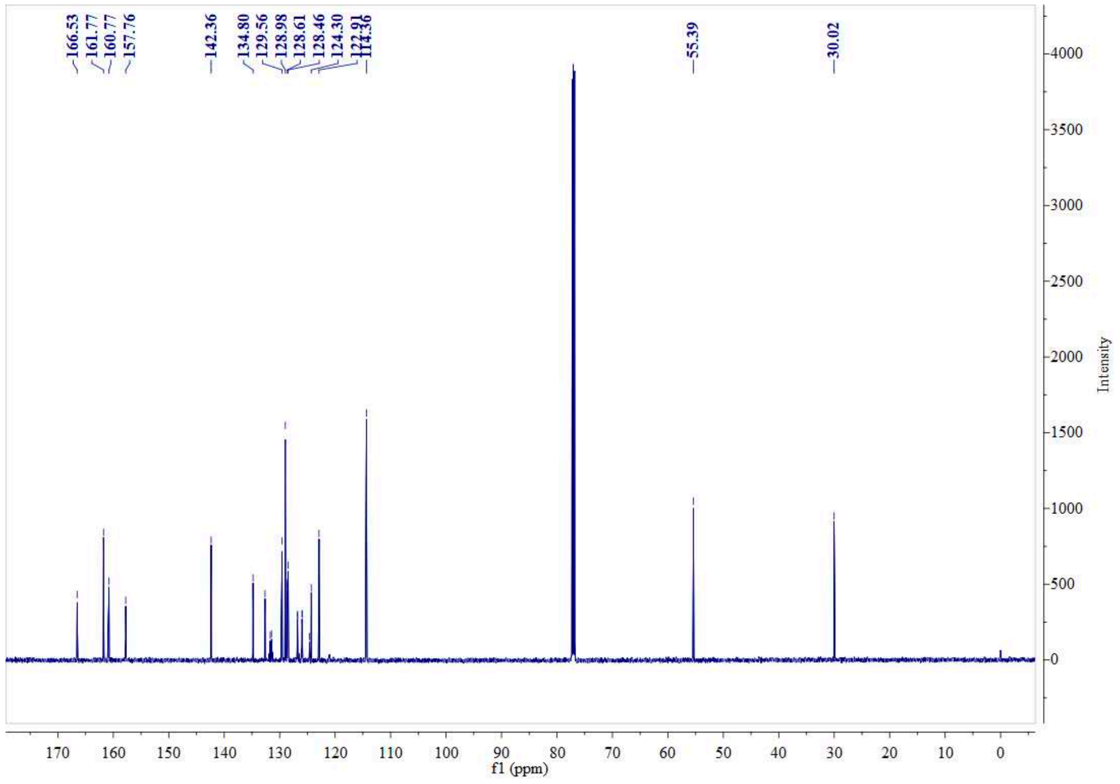

Figure S67. ^{13}C-NMR spectrum of compound **5p**.

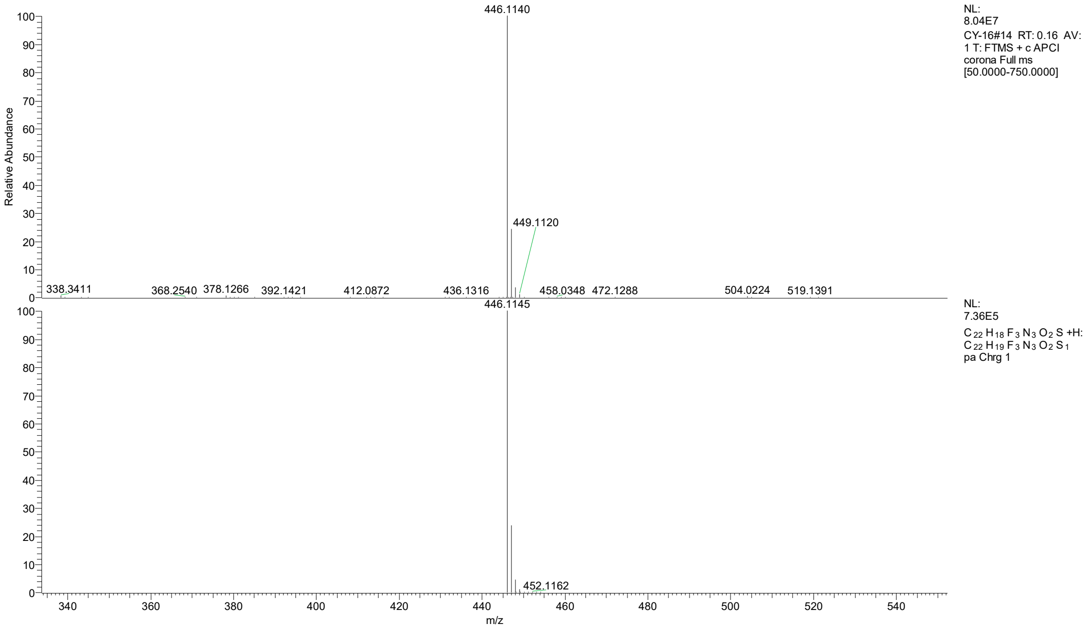

Figure S68. HRMS spectrum of compound **5p**.

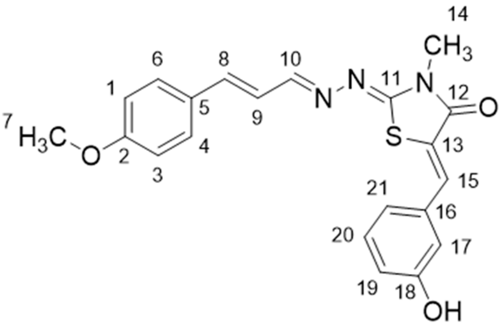

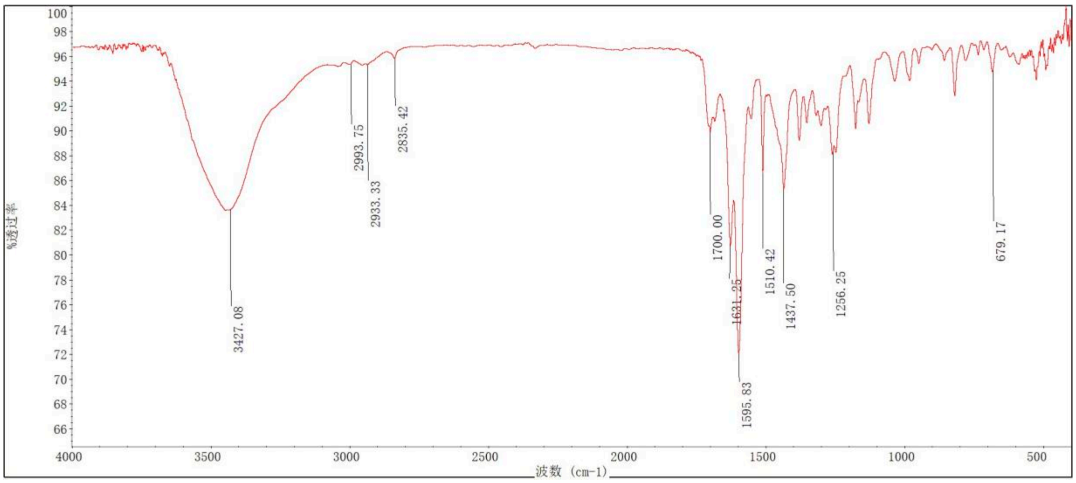

Figure S69. FT-IR spectrum of compound **5q**.

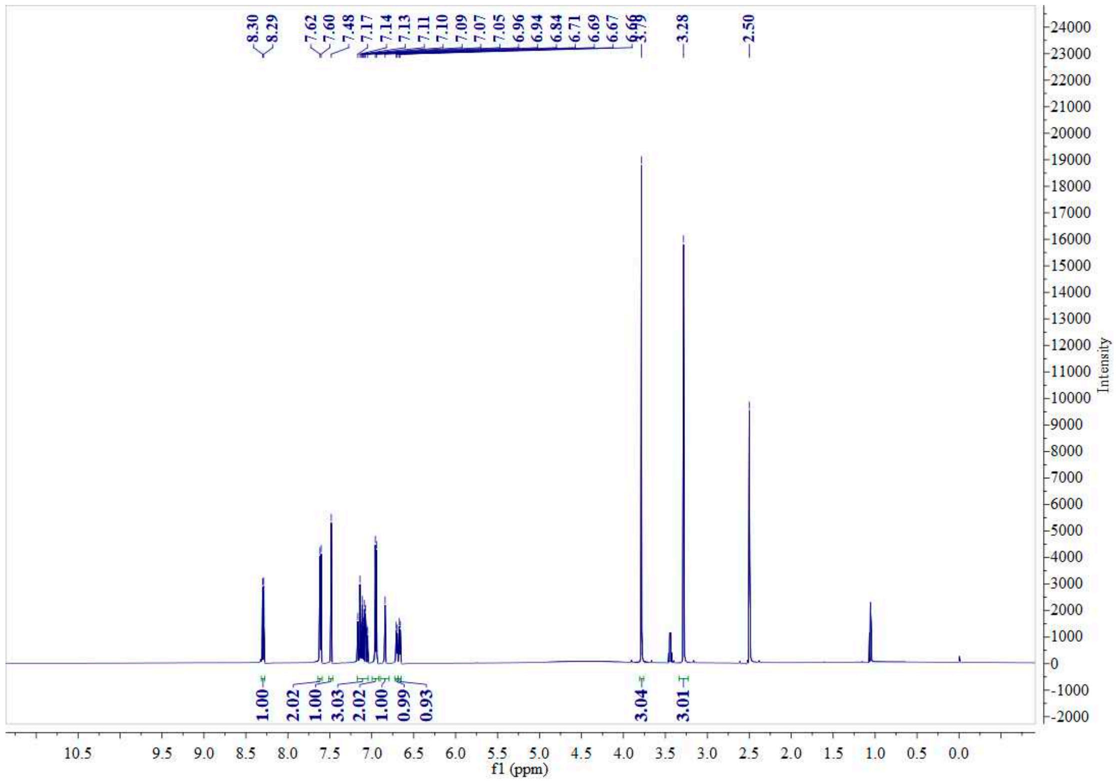

Figure S70. ^{1}H-NMR spectrum of compound **5q**.

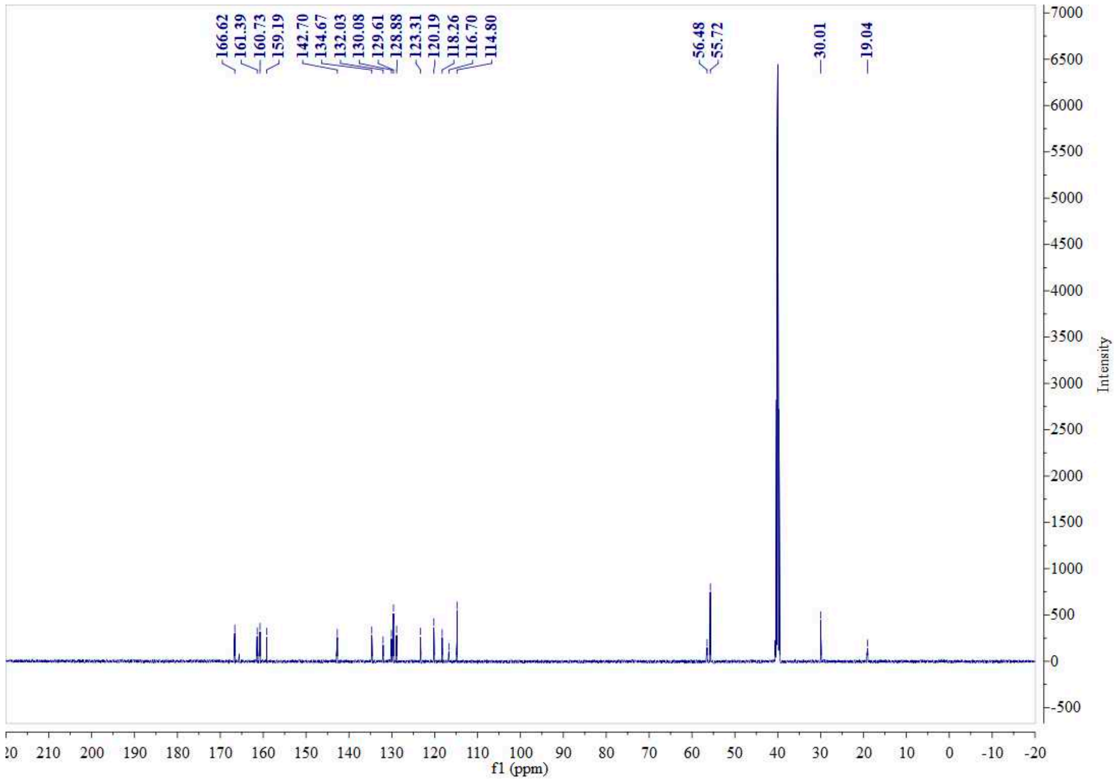

Figure S71. ^{13}C-NMR spectrum of compound **5q**.

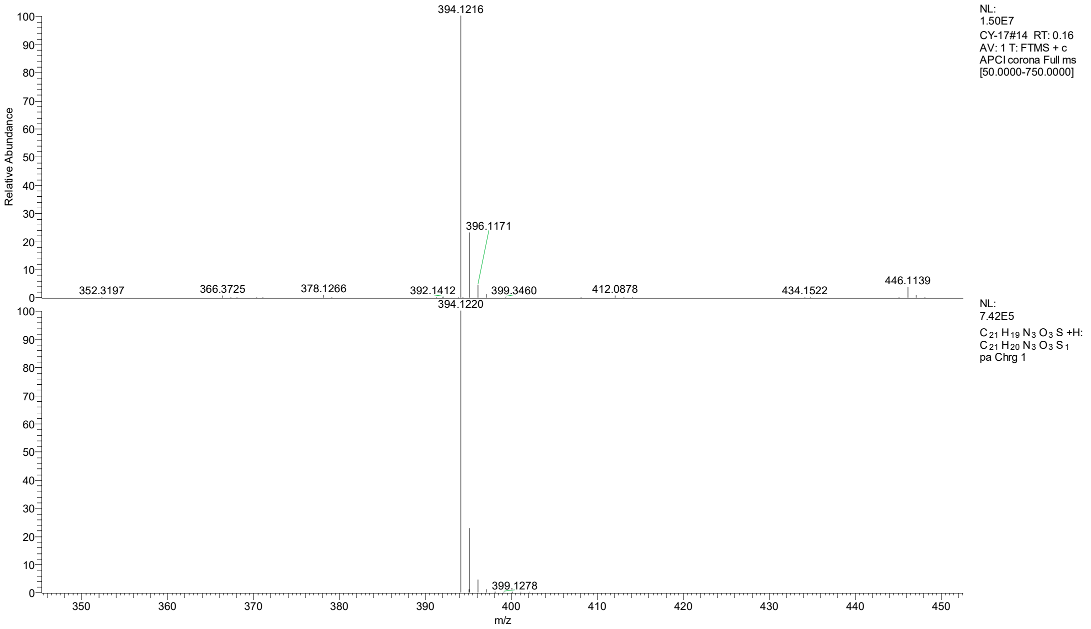

Figure S72. HRMS spectrum of compound **5q**.

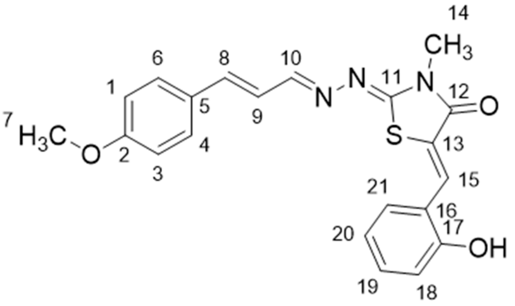

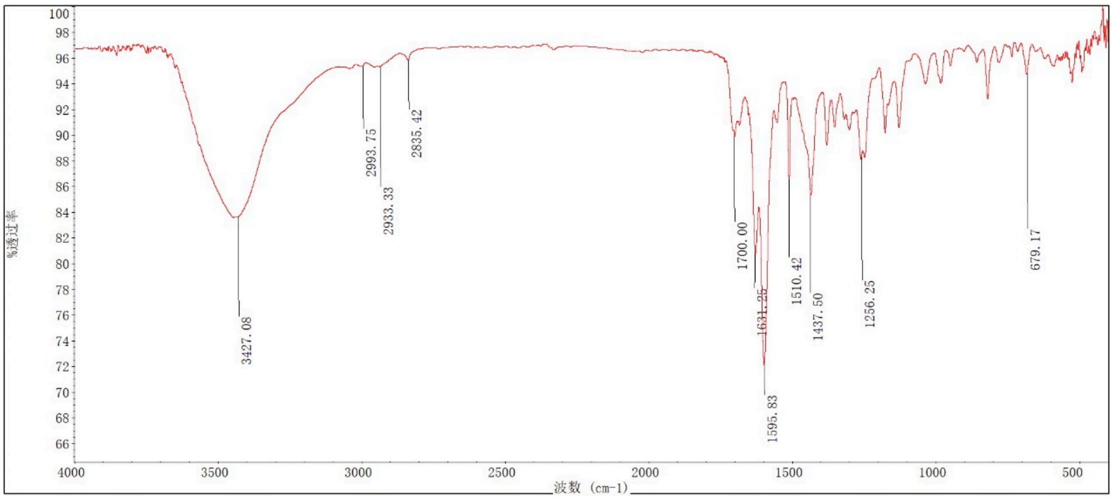

Figure S73. FT-IR spectrum of compound **5r**.

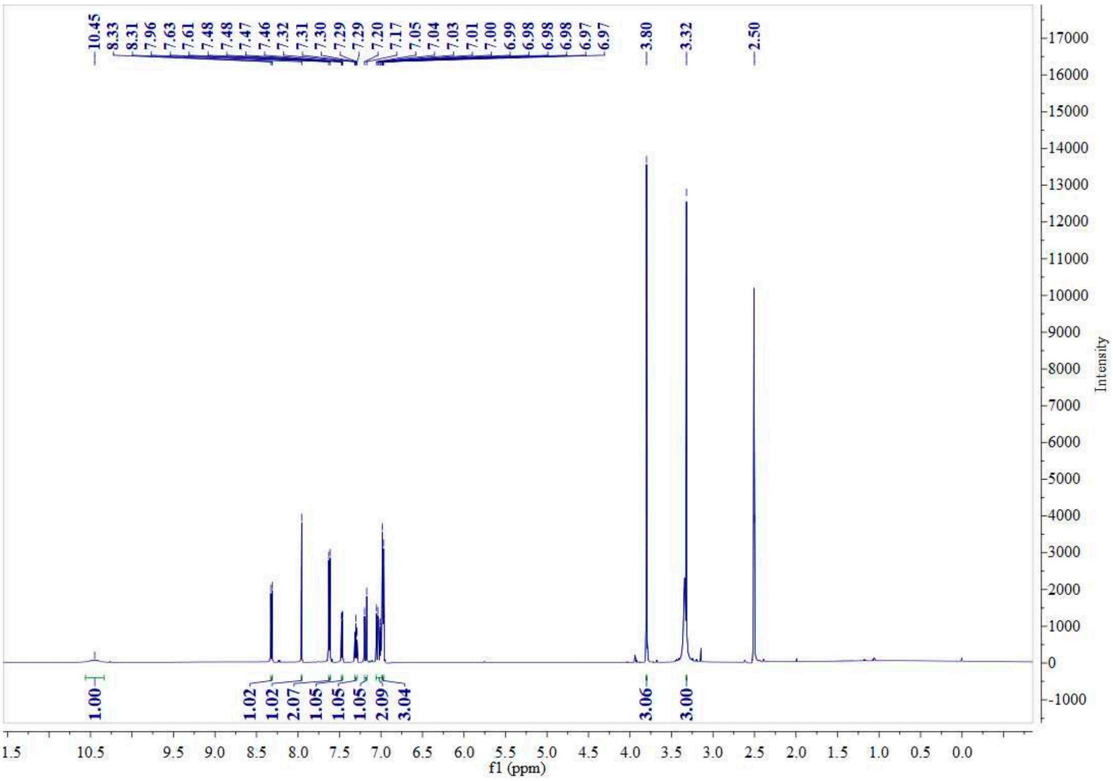

Figure S74. ^{1}H-NMR spectrum of compound **5r**.

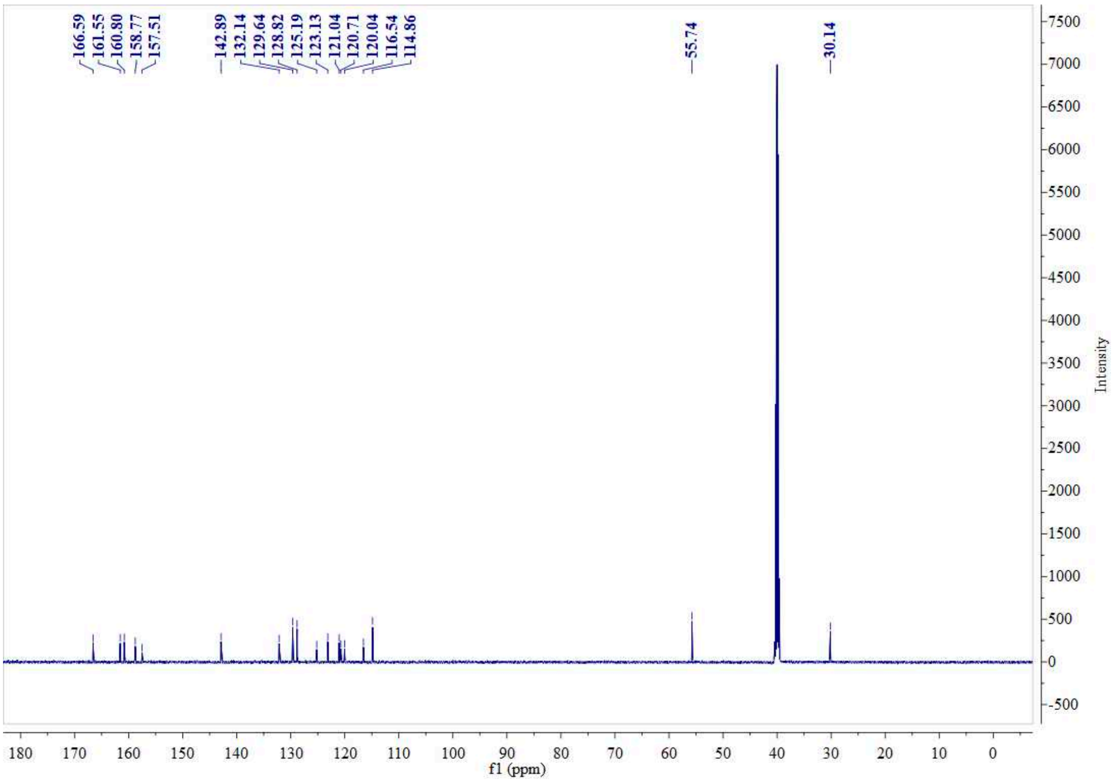

Figure S75. ^{13}C-NMR spectrum of compound **5r**.

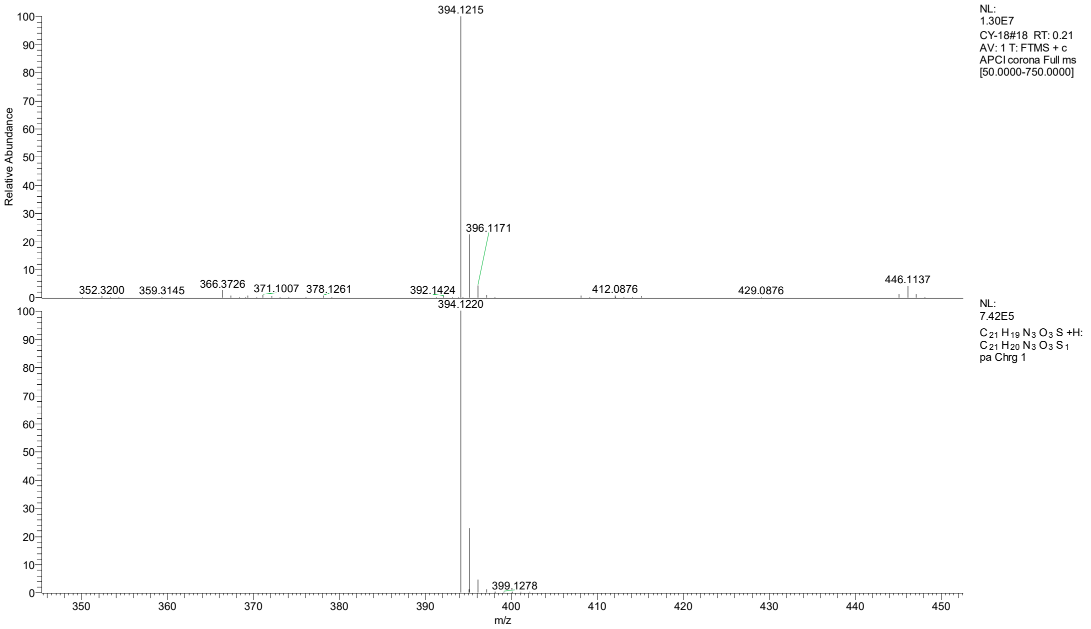

Figure S76. HRMS spectrum of compound **5r**.

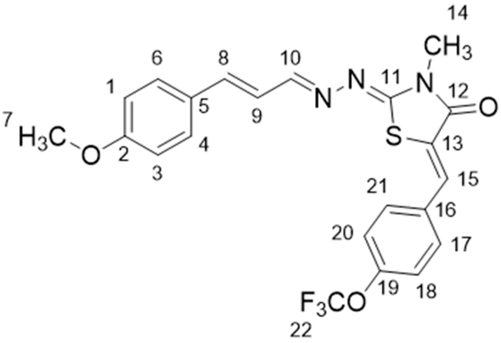

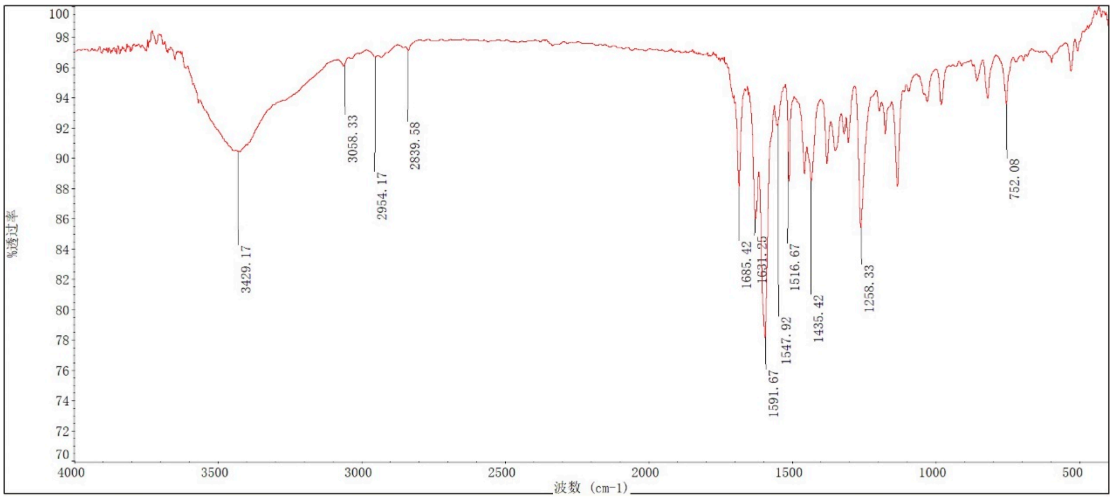

Figure S77. FT-IR spectrum of compound **5s**.

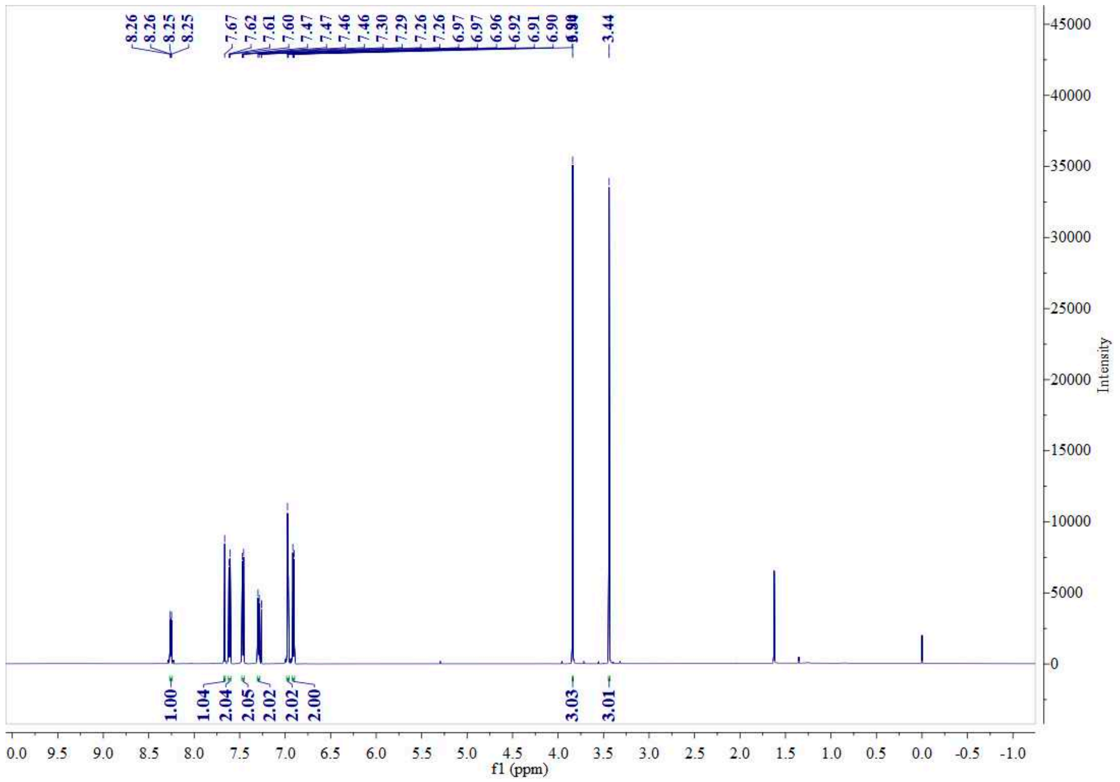

Figure S78. ^{1}H-NMR spectrum of compound **5s**.

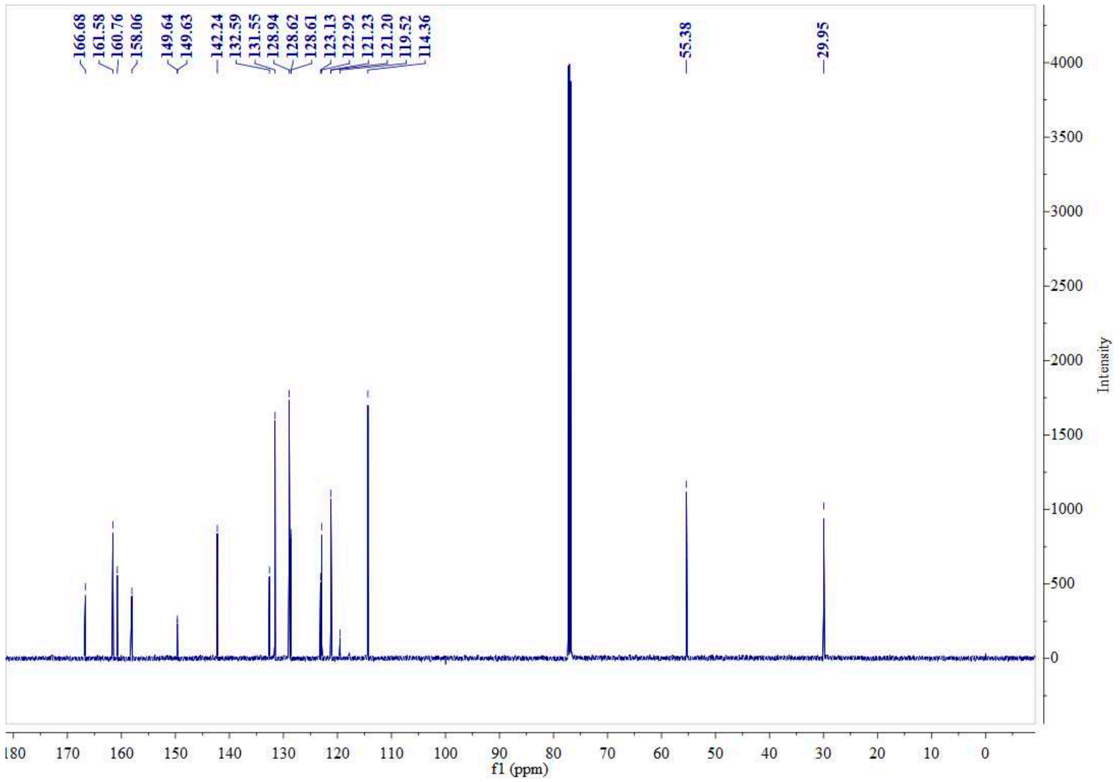

Figure S79. ^{13}C-NMR spectrum of compound **5s**.

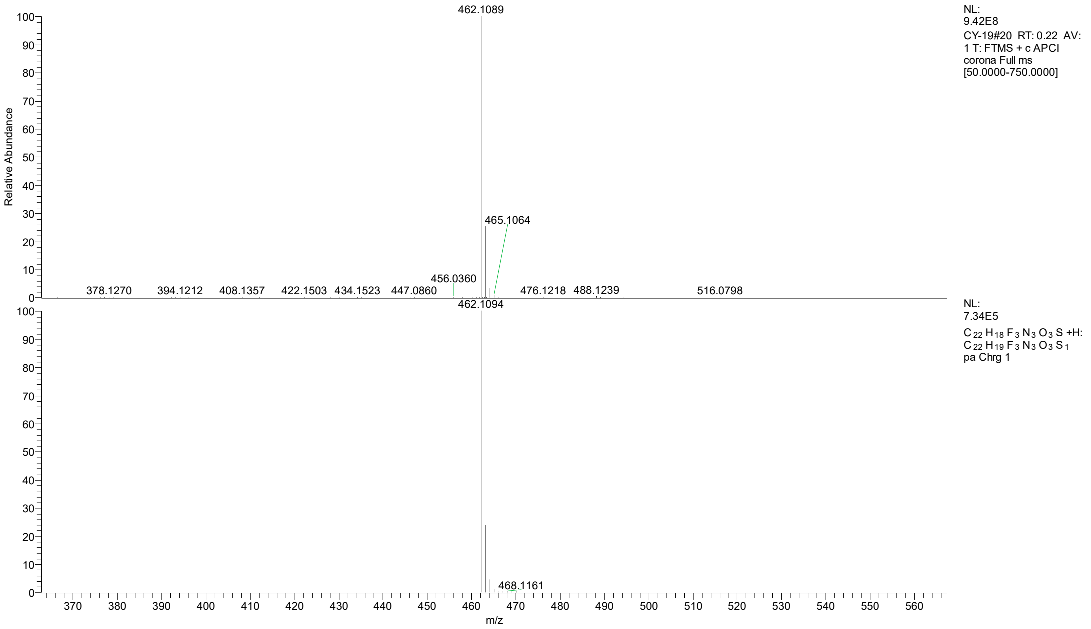

Figure S80. HRMS spectrum of compound **5s**.

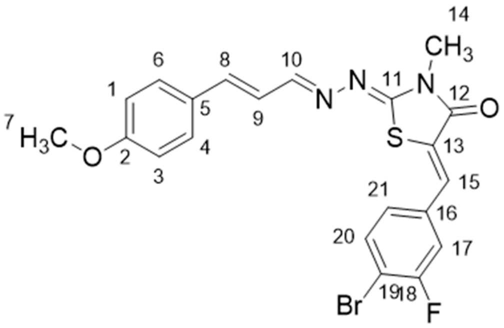

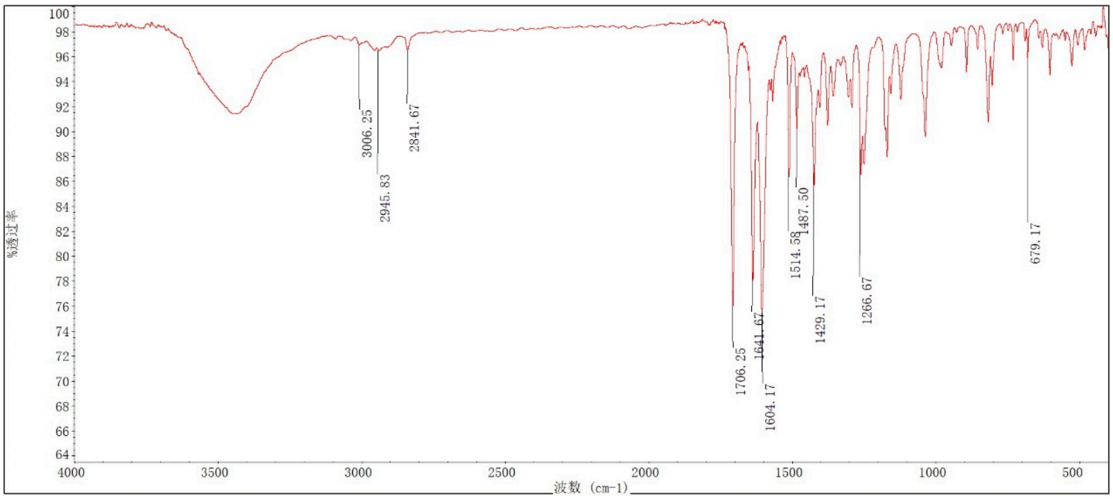

Figure S81. FT-IR spectrum of compound **5t.**

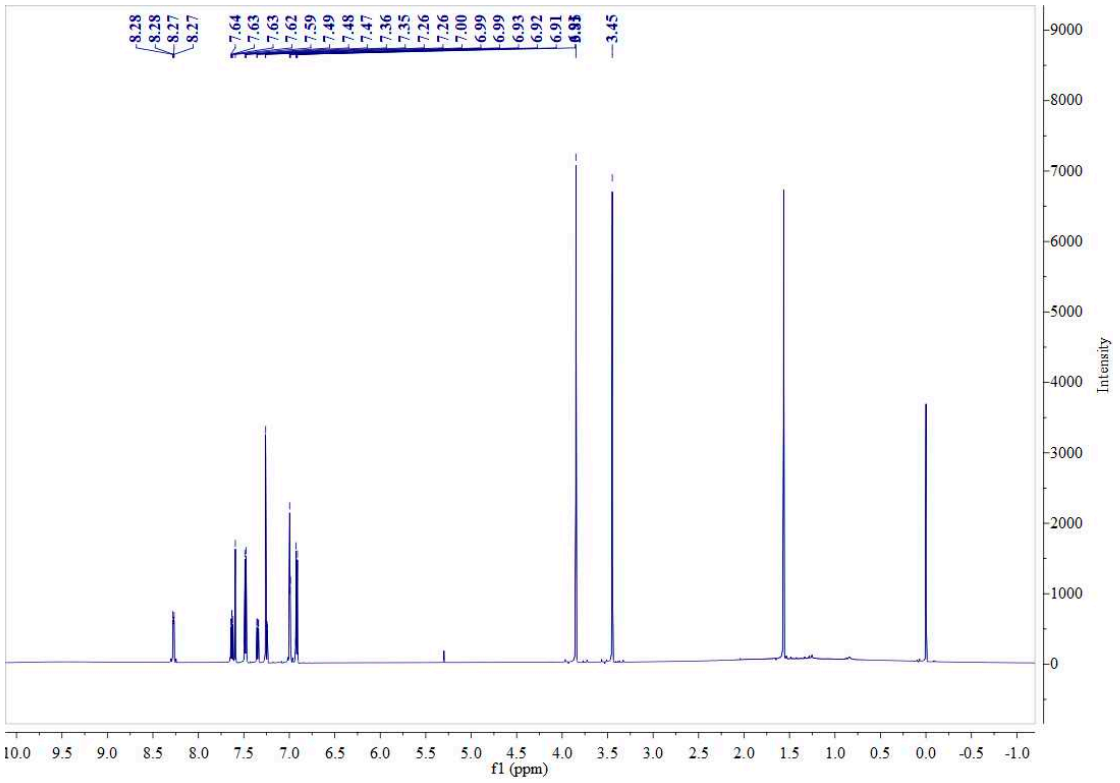

Figure S82. ^{1}H-NMR spectrum of compound **5t**.

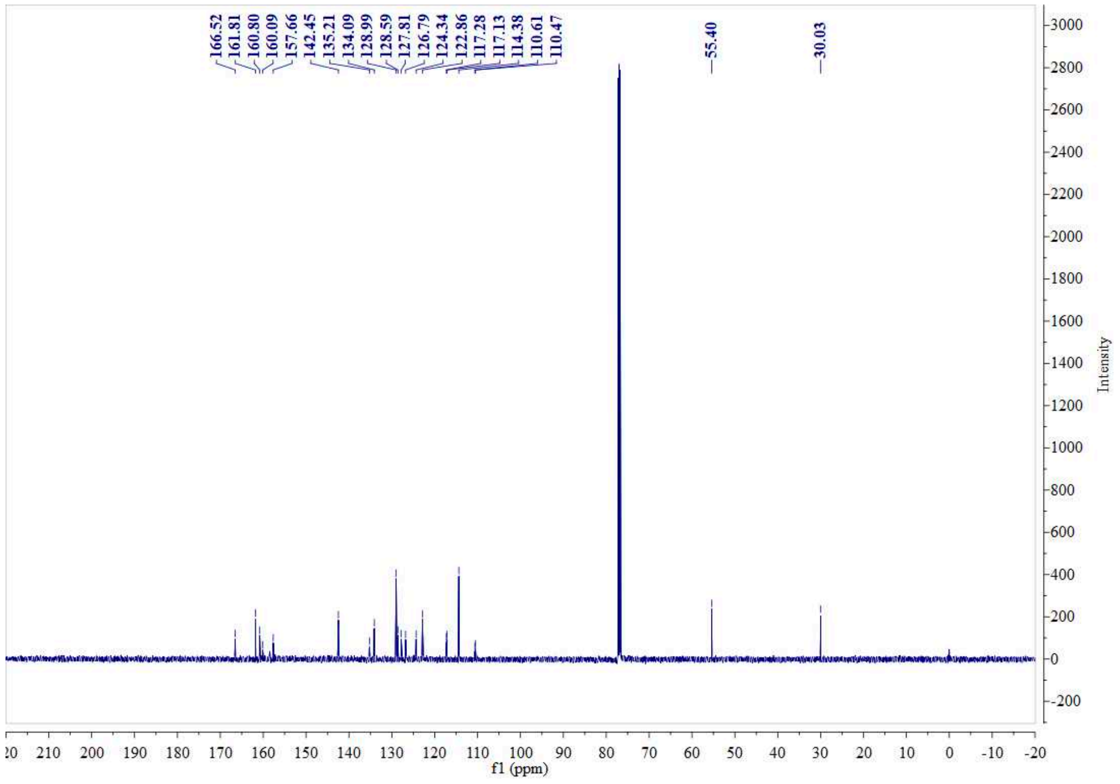

Figure S83. ^{13}C-NMR spectrum of compound **5t**.

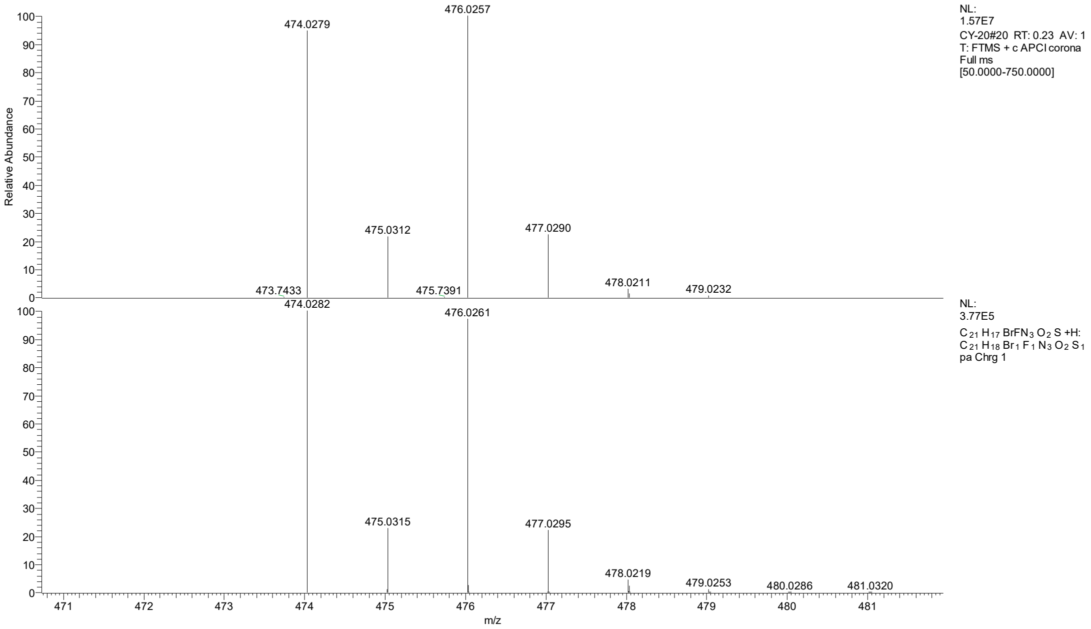

Figure S84. HRMS spectrum of compound **5t**.

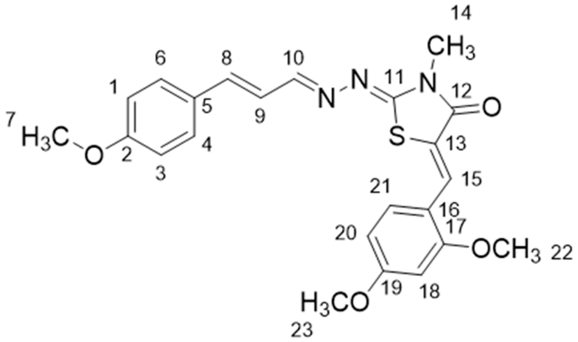

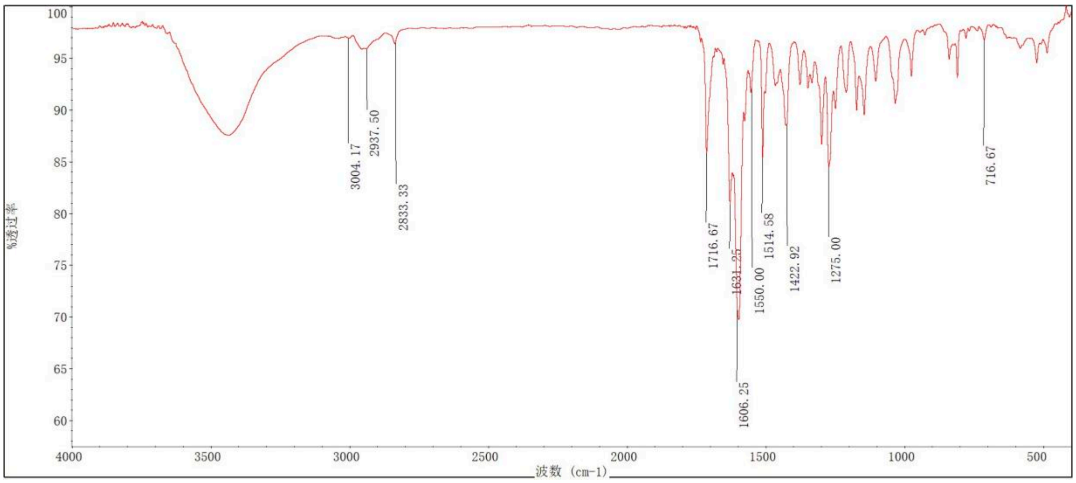

Figure S85. FT-IR spectrum of compound **5u**.

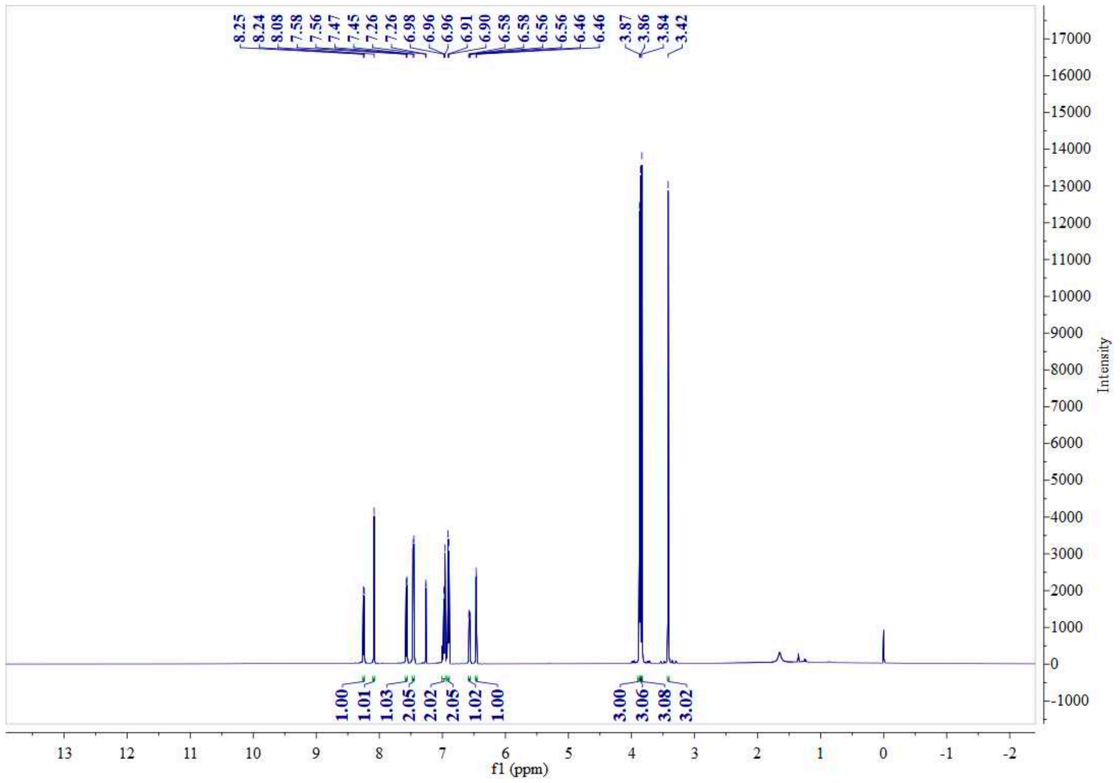

Figure S86. ^{1}H-NMR spectrum of compound **5u**.

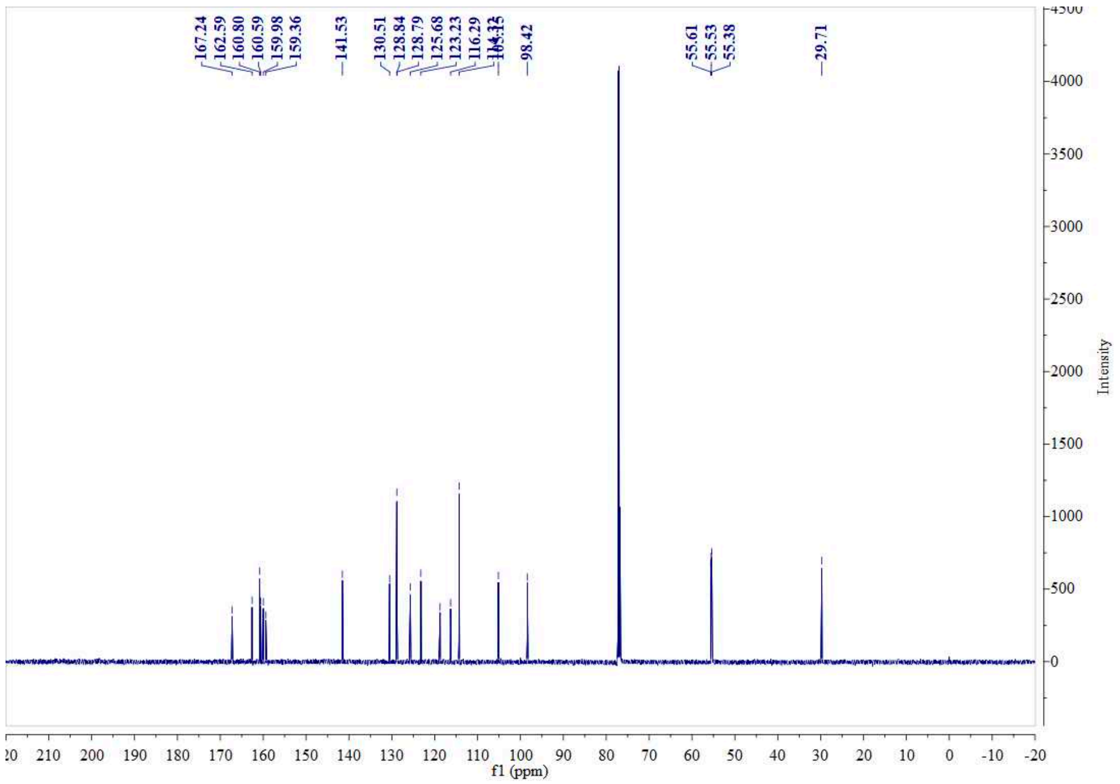

Figure S87. ^{13}C-NMR spectrum of compound **5u**.

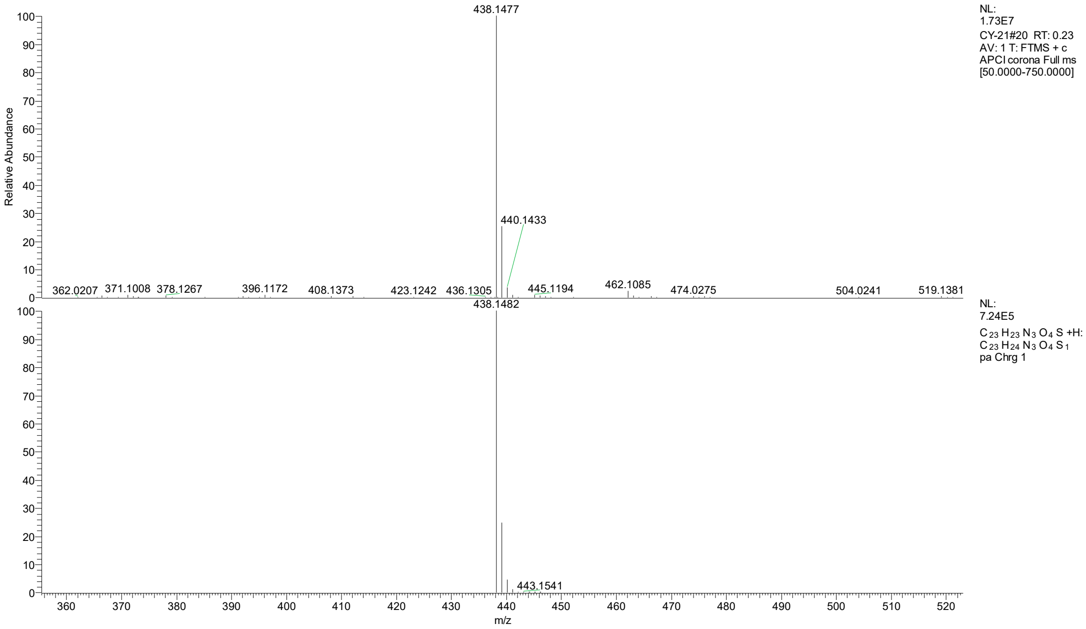

Figure S88. HRMS spectrum of compound **5u**.

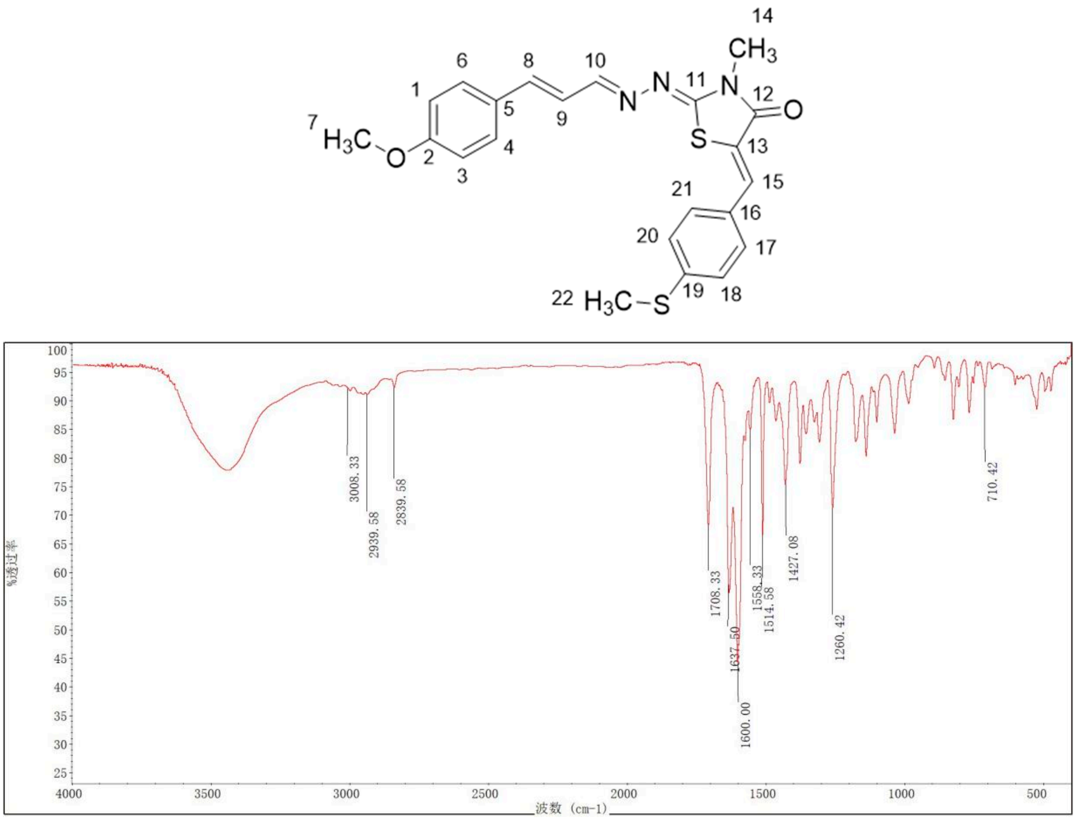

Figure S89. FT-IR spectrum of compound **5v**.

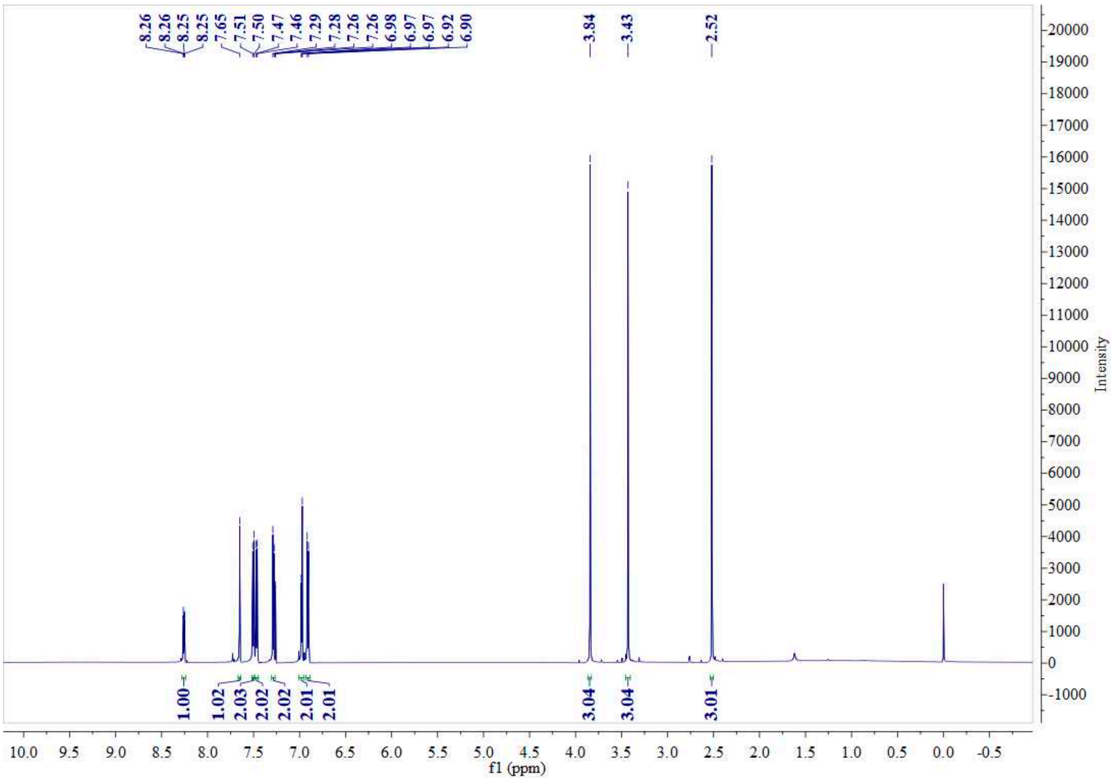

Figure S90. ^{1}H-NMR spectrum of compound **5v**.

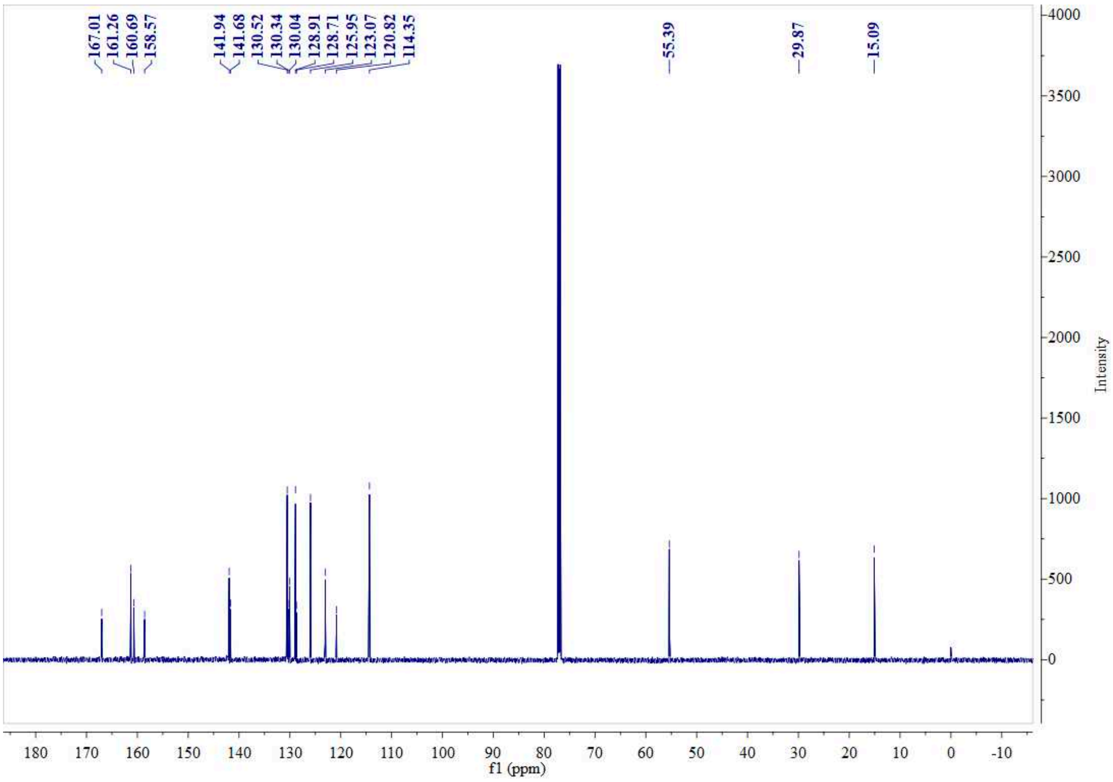

Figure S91. ^{13}C-NMR spectrum of compound **5v**.

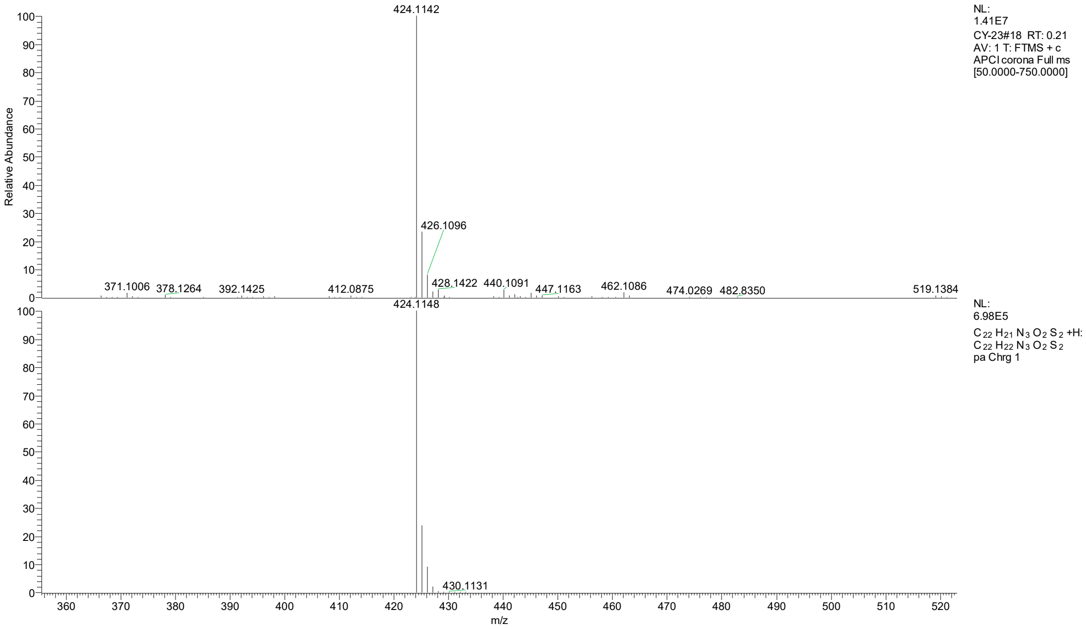

Figure S92. HRMS spectrum of compound **5v**.

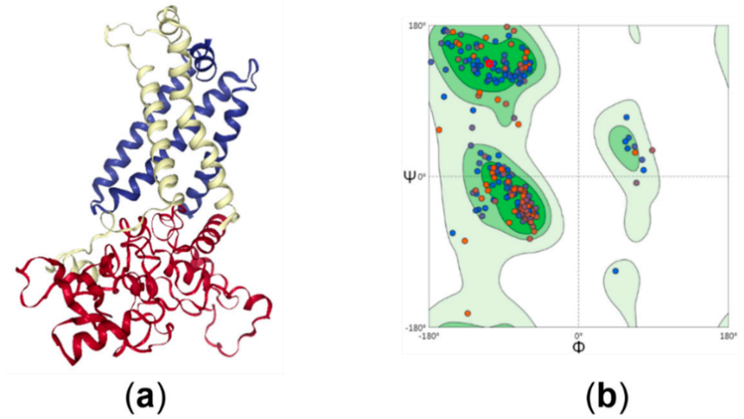

Figure S93 3D structure (a) and Ramachandran plot (b) [PDB ID: 2WQY (Remodelling of carboxin binding to the Q-site of avian respiratory complex II), homology modeling on SWISS-MODEL web]

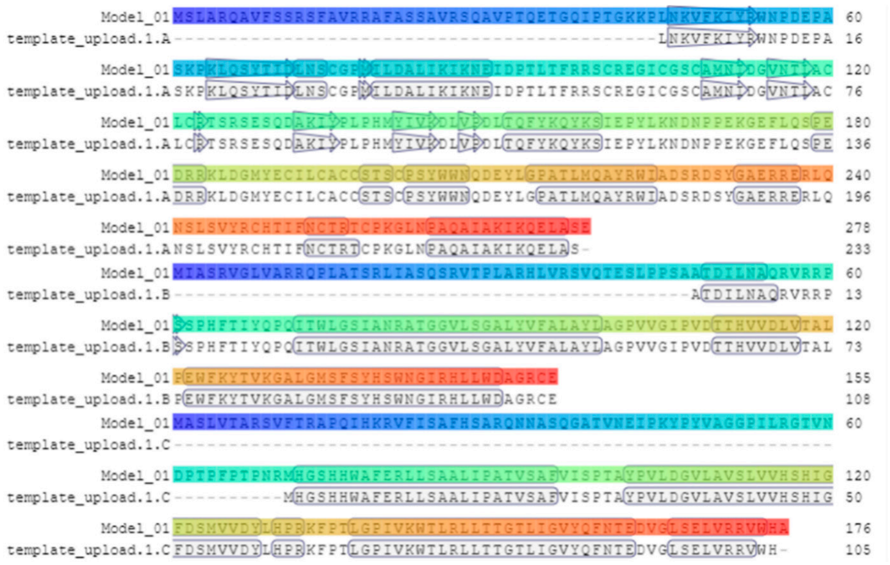

Figure S94 The model of sequence alignments based on 2WQY

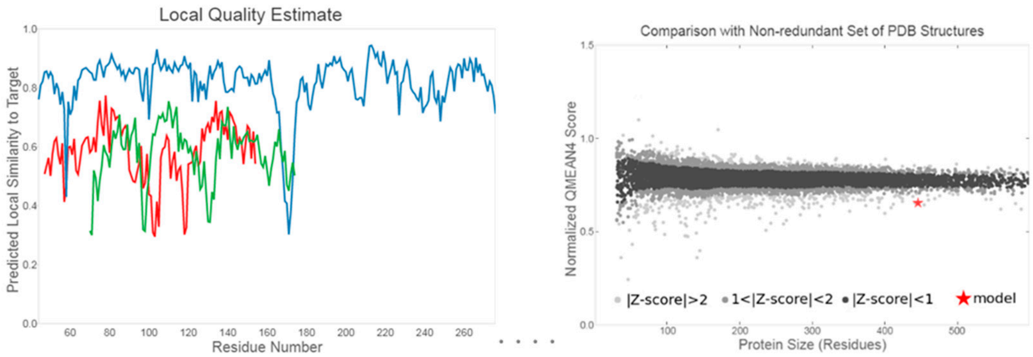

Figure S95 The model quality estimate of homology modeling
